# Tetraethylammonium Salts as Solid, Easy to Handle Ethylene Precursors and Their Application in Mizoroki–Heck Coupling

**DOI:** 10.1021/acs.joc.3c02867

**Published:** 2024-03-11

**Authors:** Eleni Papaplioura, Maëva Mercier, Michael E. Muratore, Tobias Biberger, Soufyan Jerhaoui, Michael Schnürch

**Affiliations:** † Institute of Applied Synthetic Chemistry, TU Wien, Getreidemarkt 9/163, 1060 Wien, Austria; ‡ Discovery Science, Discovery Chemistry BE, Janssen Pharmaceutica N.V., Turnhoutseweg 30, 2340 Beerse, Belgium; § Boehringer Ingelheim RCV GmbH & Co. KG, A-1121 Vienna, Austria

## Abstract

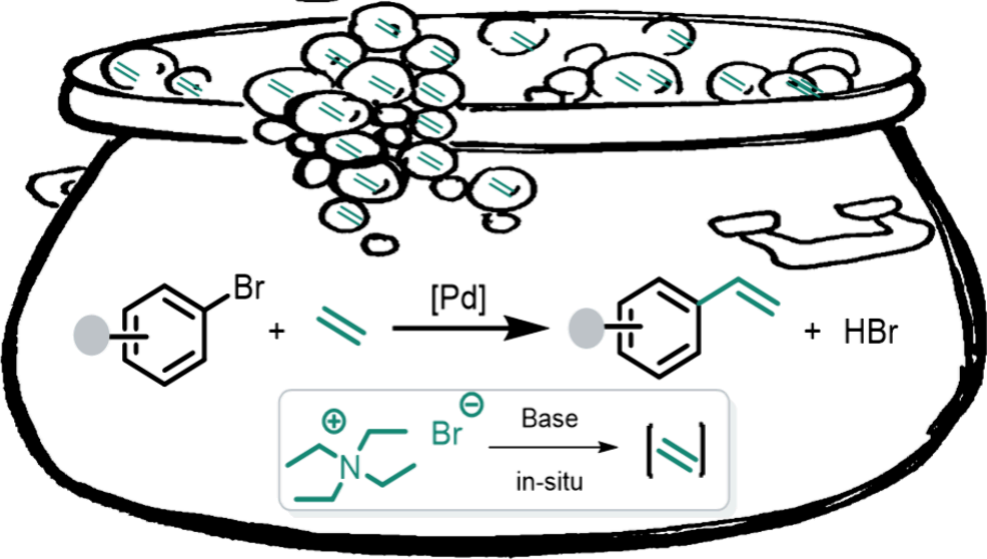

In this study, we introduce a convenient Heck vinylation protocol that eliminates the requirement for ethylene gas as a coupling partner. In contrast to traditional methodologies, quaternary ammonium salts can serve as solid olefin precursors under ambient atmosphere conditions. The practicality of this method, distinguished by its convenience and safety in a one-pot reaction, renders it appealing for applications in research and discovery context.

Arylethenes are powerful precursors in organic synthesis, used for the preparation of a broad variety of compounds with widespread pharmaceutical^1^ applications and synthetic uses.^2^ The versatility of the alkene moiety makes them excellent substrates for constructing more complex molecules. Notable transformations utilizing olefins include the Mizoroki–Heck reaction,^3−6^ the Fujiwara–Moritani reaction,^7,8^ and direct alkylation reactions through C–H functionalization.^9−11^ However, in this type of transformation, ethylene and other gaseous olefins are often avoided, primarily for practical and safety reasons. Although Heck reported on the use of ethylene as a coupling partner as early as 1968, its application has not been significantly developed since then.^4^ Conventional methodologies usually require high pressure of the olefin,^12−19^ and thus elaborate high-pressure equipment such as autoclaves or flow-chemistry reactors are needed (Figure 1). However, flow chemistry is not considered to be a standard laboratory technique and still requires ethylene pressures of >15 bar.^20,21^ To the best of our knowledge, only two papers have reported the use of ethylene at atmospheric or low pressure, but these intriguing results were not further explored.^22,23^ In a continuing research program dedicated to substituting gaseous or volatile reagents with easy-to-handle solids, we demonstrated that quaternary ammonium salts can be used as alkyl sources in direct C–H functionalization reactions.^24,25^ Herein, we describe our efforts to convert these reagents into convenient surrogates for ethylene in vinylation reactions. Substituting gaseous precursors with solid precursors not only simplifies and makes experimental setups safer but also improves the practicality and applicability of these reactions in small-scale experiments, typically encountered in academic research and medicinal chemistry programs.

**Figure 1:**
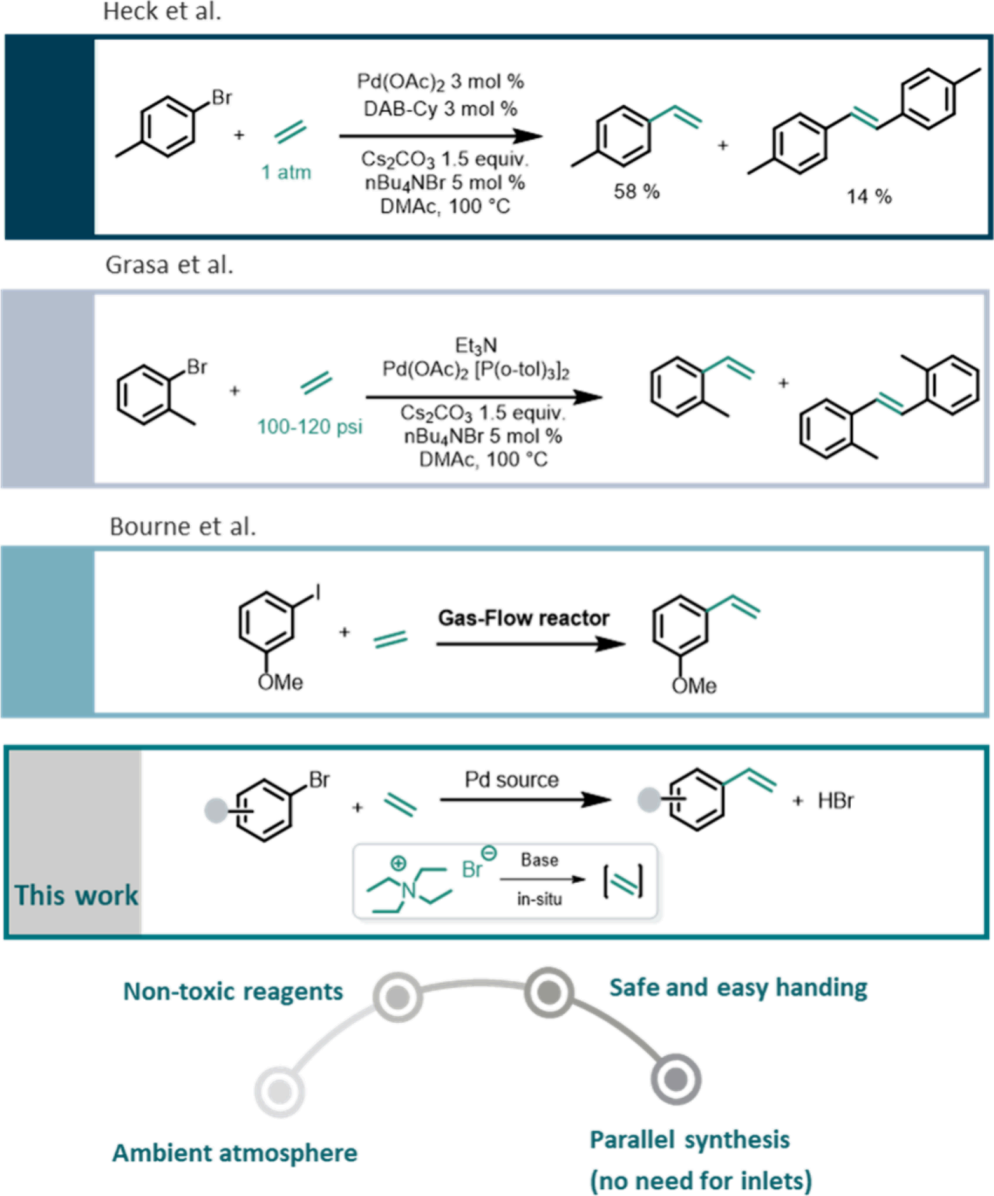
Strategies for Mizoroki–Heck coupling.

We initiated our investigations with the vinylation of 2-bromo-6-methoxynapthalene **1**, a key precursor for the synthesis of naproxen^26^ and nabumeton^27^ through the Heck reaction. Initially, we employed Et_4_NBr as the olefin surrogate, KO*t*Bu as the base, and a Pd/phosphine ligand complex as the catalytic system in toluene as solvent. Pd(OAc)_2_/(*o*-Tol)_3_P is the most commonly used catalytic system for such reactions, as originally described by Heck in 1978.^28^ Under these conditions, only a minimal amount of the corresponding styrene **2** was produced after 18 h at 100 °C (Table 1, entry 1). In the subsequent step, it was explored whether the presence of 1 wt % H_2_O at a higher temperature of 120 °C would enhance the desired transformation, as described in the literature.^29^ Indeed, compound **2** was obtained in an improved yield of 31% (entry 2). Other Pd(0) and Pd(II) sources showed lower conversions (entries 3 and 4). By employing PdXPhos G4, a Buchwald precatalyst, we achieved a substantial improvement in yield, reaching 54% (entry 5). Additionally, we examined the impact of various quaternary ammonium salts as ethylene surrogates in the reaction. We tested Et_4_NPF_6_, Et_4_NBF_4_, Et_4_NCl, and Et_4_NI, all of which resulted in moderate yields ranging from 40% to 50%, whereas PhEt_3_NI proved to be ineffective. Therefore, Et_4_NBr was chosen as the preferred olefin source for further investigation (see the complete quaternary ammonium salt screening list in the Supporting Information (SI)). Next, we conducted a screen of various bases. Although hydroxide bases are known to facilitate Hofmann elimination,^24^ when subjected to our reaction conditions, KOH only led to low conversion (entry 6), and no product was formed when employing an organic base such as DBU (entry 7) (see complete base screening list in the SI). Since styrene is prone to polymerization at temperatures exceeding 100 °C,^30^ lower temperatures were tested. This can have a second beneficial effect: the effective increase of the ethylene concentration due to its higher solubility at lower temperatures.^31^ Reducing the temperature from 120 to 100 °C significantly improved the reaction performance (entry 8). Lowering the temperature is known to be detrimental to the yield of the Hofmann elimination and prevented us from further decreasing the temperature in this methodology. Furthermore, additional precatalysts were tested at the optimal temperature of 100 °C. PdPePPSI, an NHC-type precatalyst (entry 9), formed the desired product in only 20% yield. Finally, when PdRuPhos G3 and PdJosiphos SL-J009-1 G3 were used, product **2** was obtained with a yield of 82% and 79%, respectively (entries 10 and 11) (see complete catalyst screening list in the SI). Due to the higher cost of the latter, we found the optimal conditions to be Et_4_NBr (8 equiv), KO*t*Bu (8 equiv), PdRuPhos G3 (10 mol %), in toluene (0.2 M) at 100 °C for 18 h. To address environmental concerns, we explored greener solvent alternatives, such as cyclopentylmethyl ether (CPME) and 2-methyl-THF (Me-THF). To our delight, CPME demonstrated efficiency comparable to toluene in the specific reaction, albeit with only about ∼7% lower yield. For the investigation of a scope with volatile substrates, we decided, for practical reasons, to retain toluene as the solvent.

**Table 1:**
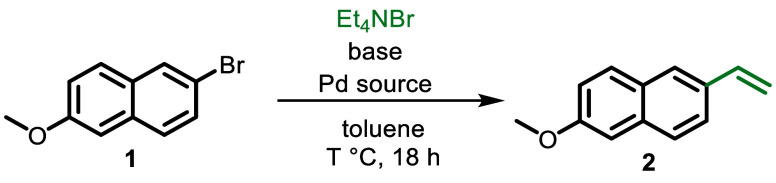
Optimization of the Reaction Conditions^a^

Entry	Base	Pd source	Temp (°C)	Yield (%)^b^
1	KO*t*Bu	Pd(OAc)_2_ + (*o*-Tol)_3_P	100	trace
2	KO*t*Bu	Pd(OAc)_2_ + 1 wt % H_2_O	120	31
3	KO*t*Bu	Pd_2_(dba)_3_	120	25
4	KO*t*Bu	Pd(PPh_3_)_4_	120	28
5	KO*t*Bu	PdXPhos G4	120	54
6	KOH	PdXPhos G4	120	20
7	DBU	PdXPhos G4	120	-
8	KO*t*Bu	PdXPhos G4	100	77
9	KO*t*Bu	PdPEPPSI	100	20
10	KO*t*Bu	PdRuPhos G3	100	82
11	KO*t*Bu	PdJosiphos SL-J009-1 G3	100	79

a Reactions were performed on a 0.2 mmol scale, with 8.0 equiv of ammonium salt, 8.0 equiv of base, and 10 mol % catalyst; reaction time: 18 h.

b Yields were determined by ^1^H NMR spectroscopy of the crude reaction mixtures using 1,3,5-trimethoxybenzene as internal standard.

With the optimized conditions established, we investigated the vinylation of various substrates, including compounds bearing electron-withdrawing or electron-donating groups, as well as heterocycles often encountered in bioactive compounds, and dihalogenated substrates (Scheme 1). Due to the highly volatile nature of the products, the yields reported below were determined by quantitative ^1^H NMR spectroscopy, unless otherwise specified. Aryl bromides bearing a trifluoromethyl group at different positions of the aryl ring were all tolerated, with the *meta*-derivative yielding 71% of the corresponding product **4**, while a notably lower yield was observed for the *ortho*-analogue **6** likely due to steric hindrance.^32^ Interestingly, the carboxylic acid moiety was also tolerated in the vinylation reaction to give product **7**, but to our surprise, that was not the case for the corresponding methyl- and *tert*-butyl- ester analogue that did not undergo the reaction. Additionally, nitro, nitrile, and aldehyde moieties were not tolerated. The reaction was also applicable to substrates bearing substituents with electron-donating properties. We systematically screened aryl bromides with methoxy functionalities (products **8**, **9**, and **10**), yielding 50% for the *meta*- and *para*-analogues and 31% for the *ortho* analogue, again presumably due to steric hindrance. Similarly, compounds with ethyl substituents (products **12**, **13**, and **14**) afforded yields of up to 60% for the ortho derivative. The 4-vinyl *Boc*-protected aniline **15** was also formed with a moderate yield of 40%. The 4-vinyl dimethylaniline **11** and the 4-vinylphenol **16** were both obtained, albeit in lower yields.

**Scheme 1:**
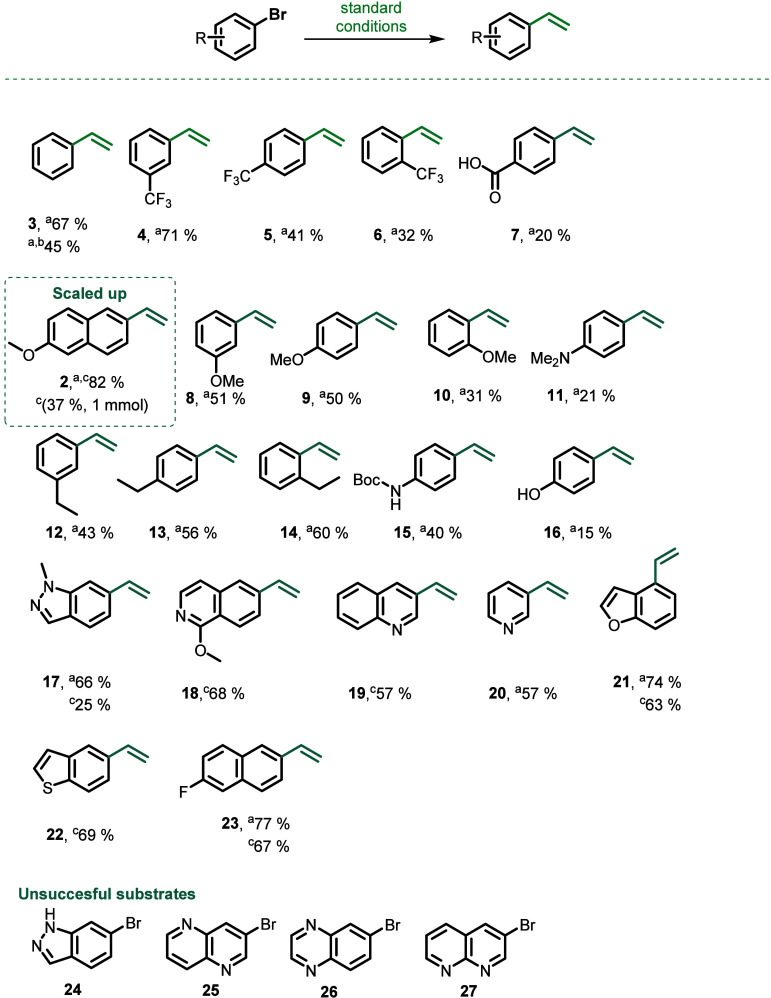
Scope of Vinylation Couplings Reactions were performed on a 0.2 mmol scale, Et_4_NBr (8 equiv), KO*t*Bu (8 equiv), PdRuPhos G3 (10 mol %) toluene (0.2 M), 100 °C for 18–40 h. Yields were determined by ^1^H NMR spectroscopy of the crude reaction mixtures using 1,3,5-trimethoxybenzene as internal standard. Iodobenzene was used as starting material instead of bromobenzene. Isolated yields.

It is worth noting that we observed complete substrate consumption in all cases and there was no formation of the corresponding stilbene side product, which may have formed by a subsequent Heck reaction with the desired product. However, we did not achieve quantitative yields in any case. Polystyrene-derivatives are most likely formed,^32^ but could not be detected *via* GC-MS or NMR analysis possibly due to their (expected) low solubility in common organic solvents. Additionally, we explored heteroaromatic compounds related to bioactive molecules, producing the corresponding vinylated products in moderate to high yields. It should be noted that the lower isolated yields in contrast to the ^1^H NMR yields were attributed to the volatility of the respective compounds upon purification. We were delighted to find that various nitrogen-containing heterocycles, compounds with diverse chemical properties, and vast applications, particularly in medicinal chemistry, were compatible with our methodology. Compounds including pyridine, indazole, or (iso)quinoline motifs, were tested using our novel protocol. 1-Methyl-6-vinyl-1*H*-indazole **17** and 1-methoxy-6-vinylisoquinoline **18** were obtained with a 66% and 68% yield respectively, while 3-vinylquinoline **19** was formed in 57% yield. An extended reaction time of up to 40 h was required for the complete conversion of 3-bromopyridine into desired product **20**. Similar reactivity was observed for heteroarenes such as 4-bromobenzofuran and 5-bromobenzothiophene yielding 74% and 70% of the corresponding products **21** and **22**, respectively.

Furthermore, 2-vinyl-6-fluoronapthalene **23** was also tolerated and formed in a yield of 77%. The presence of the free NH group in 6-bromo-1*H*-indazole **24** appeared to be problematic for the reaction to proceed, whereas 3-bromo-1,5-naphthyridine **25**, 6-bromoquinoxaline **26**, and 3-bromo-1,8-naphthyridine **27** surprisingly did not undergo the transformation (for the complete heterocycle scope screening, see the SI). Finally, the selected substrate for optimizing this protocol, 2-bromo-6-methoxynapthalene, gave the respective vinylated product **2** with an isolated yield of 82%. Increasing the scale in this example from 0.2 mmol to intermediate scales of 0.4 and 0.6 mmol and eventually to 1 mmol scale led to a constant decrease in yield as follows: 71%, 65%, and 37%, respectively.

In conclusion, we have presented a novel protocol for the facile vinylation of aryl bromides, utilizing tetraethylammonium salts as surrogates for gaseous olefins in a one-pot reaction conducted under an ambient atmosphere. We have successfully demonstrated this methodology with a diverse set of 25 examples, ranging from simple aryl bromides to heterocycles, providing vinyl(hetero)arenes in chemical yields ranging from 10% to 80%. It is important to note that this represents just the initial phase toward achieving a convenient Heck reaction. Further efforts are in progress to identify conditions that will enable a broader scope. Moreover, the applicability and practicality of this transformation make it well-suited for use in a discovery medicinal chemistry context where operationally complex reaction setups, such as the manipulation of gases, are not common practice.

## Experimental Section

Unless noted otherwise, the reactants and reagents were purchased from commercial suppliers and used without further purification. The 4 mL brown-glass vials were sealed with Wheaton screw caps containing a PTFE faced 14B styrene-butadiene rubber liner. All reactions were magnetically stirred and heated in a metallic reaction block. All reaction temperatures refer to external temperatures.

**NMR** spectra were recorded in CDCl_3_ or *d*_*8*_-toluene on a Bruker Avance UltraShield (400 MHz) or Avance III HD 600 (600 MHz) spectrometer, and chemical shifts (δ) are reported in ppm, using Me_4_Si as internal standard. Coupling constants (*J*) are given in hertz (Hz), and multiplicities are assigned by the following abbreviations = singlet, d = doublet, t = triplet, q = quartet, and m = multiplet.

**Thin Layer Chromatography (TLC)** analysis was performed on precoated aluminum-backed unmodified plates (Silica gel 60 F_254_, Merck). Compounds were visualized under UV light.

**Flash column chromatography** was performed using either Merck silica gel 60 (40 μm–63 μm) by hand column, or purification was performed using a TELEDYNE ISCO COMBI FLASH COMPANION NextGen300+ flash chromatography system with a UV–vis detector (diode array) with Macherey-Nagel Chromabond RS silica gel columns.

**GC-MS** analysis was performed on a Thermo Finnigan Focus GC/DSQ II with a standard capillary column RXi-5Sil MS column (30 m, 0.25 mm ID, 0.25 μm df) using the following standardized temperature programs: **Method A:** 2 min at 100 °C, 35 °C/min until 300 °C, 4 min at 300 °C and **Method B:** 2.5 min at 40 °C, 12 °C/min until 220 °C, 2.5 min at 220 °C.

**HR-MS** data were recorded using a Thermo Scientific Orbitrap Elite hybrid ion trap/orbitrap spectrometer system with an Ultimate 3000 series LPG-3400XRS pump system. Mass calibration was performed using the Pierce LTQ Velos ESI positive ion calibration solution from Thermo Scientific (lot PF200011, product no. 88323).

Reaction Optimization Screening. The optimization of reaction conditions was carried out following general procedure A (see the SI for details). Yields were determined by ^1^H NMR using 1,3,5-trimethoxybenzene as an internal standard.

### General Procedure A

To a 4 mL brown vial equipped with a magnetic stirring bar and a Wheaton screw cap were added 2-bromo-6-methoxynapthalene (**1**) (48.4 mg, 0.2 mmol, 1 equiv), the respective ammonium salt (8 equiv), the base (8 equiv), and the catalyst (amount as specified in the optimization table). Subsequently, solvent (0.2 M) was added via syringe, and the reaction mixture was heated to 120 °C, or to 100 and 80 °C, depending on the corresponding solvent in a metallic block for 18–36 h respectively. For the sample preparation for ^1^H NMR quantification, the reaction mixture was cooled to room temperature and filtered over Celite in a syringe filter. The filter cake was washed with EtOAc, and the volatiles were removed in vacuo. 1,3,5-Trimethoxybenzene (0.2 mmol, 1 equiv) was added, and the crude mixture was dissolved in 0.5 mL of CDCl_3_ and transferred to an NMR tube. The recorded spectra were processed by MestReNova v14 software.

### General Procedure B

To a 4 mL brown vial equipped with a magnetic stirring bar and a Wheaton screw cap were added the starting material (0.2 mmol, 1 equiv), Et_4_NBr (8 equiv), KO*t*Bu (8 equiv), and PdRuPhos G3 (10 mol %). Subsequently, *d*_*8*_-toluene (0.2 M) was added *via* syringe, and the reaction mixture was heated to 100 °C in a metallic block for 18–36 h. The reaction mixture was cooled down to room temperature and filtered in a syringe filter. The filter cake was washed with *d*_*8*_-toluene, and 1,3,5-trimethoxybenzene (0.2 mmol, 1 equiv) was added to the crude reaction mixture. To an NMR tube was transferred 0.5 mL of the reaction solution, and the recorded spectra were processed by MestReNova v14.

### General Procedure C

To a 4 mL brown vial equipped with a magnetic stirring bar and a Wheaton screw cap were added the starting material (0.2 mmol, 1 equiv), Et_4_NBr (8 equiv), KO*t*Bu (8 equiv), and PdRuPhos G3 (10 mol %). Subsequently, toluene (0.2 M) was added *via* a syringe, and the reaction mixture was heated to 100 °C, in a metallic block for 18–36 h. Workup Procedure 1: The reaction mixture was cooled down to room temperature and filtered in a syringe filter. The filter cake was washed with EtOAc, and the volatiles were removed in *vacuo*. Workup Procedure 2: The reaction mixture was cooled down to room temperature and filtered in a syringe filter, and toluene was removed in *vacuo*. The crude mixture was redissolved in DCM, and water was added (HCl 1 M was added for neutralization). The aqueous phase was extracted with DCM (5×), the combined organic extracts were dried over Na_2_SO_4_, and the volatiles were removed in *vacuo*. 1,3,5-Trimethoxybenzene (0.2 mmol, 1 equiv) was added, and the crude mixture was dissolved in 0.5 mL of CDCl_3_ and transferred to an NMR tube. The recorded spectra were processed by MestReNova v14 software. The crude residue was purified either *via* manual column chromatography using unmodified silica or by using an automated purification system.

### General Procedure D

To a 4 mL brown vial equipped with a magnetic stirring bar and a Wheaton screw cap were added the starting material (0.2 mmol, 1 equiv), Et_4_NBr (8 equiv), KO*t*Bu (8 equiv), and PdXPhos G (10 mol %). Subsequently, toluene (0.2 M) was added *via* syringe, and the reaction mixture was heated to 100 °C in a metallic block for 18–20 h. Workup Procedure 1: The reaction mixture was cooled down to room temperature and filtered in a syringe filter. The filter cake was washed with EtOAc, and the volatiles were removed in *vacuo*. 1,3,5-Trimethoxybenzene (0.2 mmol, 1 equiv) was added, and the crude mixture was dissolved in 0.5 mL of CDCl_3_ and transferred to an NMR tube. The recorded spectra were processed by MestReNova v14 software. The crude residue was purified either *via* manual column chromatography using unmodified silica or using an automated purification system.

### General Procedure E

To a 4 mL brown vial equipped with a magnetic stirring bar and a Wheaton screw cap were added the starting material (0.2 mmol, 1 equiv), Et_4_NBr (8 equiv), KO*t*Bu (8 equiv), and PdXPhos G4 (10 mol %). Subsequently, *d*_*8*_-toluene (0.2 M) was added *via* syringe, and the reaction mixture was heated to 100 °C in a metallic block for 18 h. The reaction mixture was cooled down to room temperature and filtered in a syringe filter. The filter cake was washed with *d*_*8*_-toluene, and 1,3,5-trimethoxybenzene (0.2 mmol, 1 equiv) was added to the crude reaction mixture. To an NMR tube was transferred 0.5 mL of the reaction solution, and the recorded spectra were processed by MestReNova v14.

### 2-Vinyl-6-methoxynaphthalene^33^ (2)

The title compound was prepared according to general procedure C from commercially available starting material with a reaction time of 20 h following Workup Procedure 1. The crude residue was filtered over a short plug of silica using pentane to give the product as a white solid. ^1^H NMR yield (based on the integration of peaks at 6.09 and 5.80 ppm): 82%. Isolated yield: (30 mg, 81%). ^1^H NMR (400 MHz, CDCl_3_) δ 7.77–7.67 (m, 3H), 7.60 (dd, *J* = 8.8, 1.6 Hz, 1H), 7.19–7.09 (m, 2H), 6.85 (dd, *J* = 17.6, 10.8 Hz, 1H), 5.82 (dd, *J* = 17.6, 0.9 Hz, 1H), 5.28 (dd, *J* = 10.8, 0.9 Hz, 1H), 3.92 (s, 3H). ^13^C{1H} NMR (101 MHz, CDCl_3_) δ: 157.9, 137.1, 134.5, 133.1, 129.7, 129.1, 127.1, 126.3, 123.9, 119.1, 113.2, 106.0, 55.5. Compound **2** was also prepared on a 0.4 mmol scale as described above: ^1^H NMR yield (based on the integration of peaks at 6.09 and 5.80 ppm): 71%, on a 0.6 mmol scale: ^1^H NMR yield (based on the integration of peaks at 6.09 and 5.80 ppm): 65%, and on a 1 mmol scale (1 equiv) using Et_4_NBr (8 equiv), KO*t*Bu (8 equiv), and PdRuPhos G3 (10 mol %). Isolated yield: (68 mg, 37%, 0.148 mmol).

### Styrene^34^ (3)

The title compound was prepared according to general procedure B from commercially available starting materials with a reaction time of 20 h. From bromobenzene [CAS 108-86-1]: ^1^H NMR yield (based on the integration of peaks at 6.10 and 5.58 ppm): 67%, and from iodobenzene [CAS 591-50-4]: ^1^H NMR yield (based on the integration of peaks at 6.10 and 5.59 ppm): 45%.

### 1-(Trifluoromethyl)-3-vinylbenzene^35^ (4)

The title compound was prepared according to general procedure B from commercially available starting material with a reaction time of 18 h. ^1^H NMR yield (based on the integration of peaks at 6.19 and 5.49 ppm): 71%.

### 1-(Trifluoromethyl)-4-vinylbenzene^35^ (5)

The title compound was prepared according to general procedure B from commercially available starting material with a reaction time of 18 h. ^1^H NMR yield (based on the integration of peaks at 6.13 and 5.47 ppm): 41%. The title compound was also prepared according to general procedure E from commercially available starting material with a reaction time of 18 h. ^1^H NMR yield (based on the integration of peaks at 6.13 and 5.47 ppm): 9%.

### 1-(Trifluoromethyl)-2-vinylbenzene^36^ (6)

The title compound was prepared according to general procedure B from commercially available starting material with a reaction time of 40 h. ^1^H NMR yield (based on the integration of peaks at 6.13 and 5.48 ppm): 32%.

### 4-Vinylbenzoic Acid^37^ (7)

The title compound was prepared according to general procedure B from commercially available starting material with a reaction time of 18 h. ^1^H NMR yield (based on the integration of peaks at 6.13 and 5.57 ppm): 20%. The title compound was also prepared according to general procedure E from commercially available starting material with a reaction time of 18 h. ^1^H NMR yield (based on the integration of peaks at 6.13 and 5.57 ppm): 31%.

### 1-Methoxy-3-vinylbenzene^38^ (8)

The title compound was prepared according to general procedure B from commercially available starting material with a reaction time of 18 h. ^1^H NMR yield (based on the integration of peaks at 6.13 and 5.78 ppm): 51%. The title compound was prepared according to general procedure E from commercially available starting material with a reaction time of 18 h. ^1^H NMR yield (based on the integration of peaks at 6.13 and 5.78 ppm): 51%.

### 1-Methoxy-4-vinylbenzene^39^ (9)

The title compound was prepared according to general procedure B from commercially available starting material with a reaction time of 40 h. ^1^H NMR yield (based on the integration of peaks at 6.13 and 5.54 ppm): 50%.

### 1-Methoxy-2-vinylbenzene^38^ (10)

The title compound was prepared according to general procedure B from commercially available starting material with a reaction time of 40 h. ^1^H NMR yield (based on the integration of peaks at 6.13 and 5.71 ppm): 31%.

### *N*,*N*-Dimethyl-4-vinylaniline^40^ (11)

The title compound was prepared according to general procedure B from commercially available starting material with a reaction time of 18 h. ^1^H NMR yield (based on the integration of peaks at 6.13 and 5.57 ppm): 21%.

### 1-Ethyl-3-vinylbenzene (12)

The title compound was prepared according to general procedure B from commercially available starting material with a reaction time of 18 h. ^1^H NMR yield (based on the integration of peaks at 6.13 and 5.67 ppm): 43%.

### 1-Ethyl-4-vinylbenzene^35^ (13)

The title compound was prepared according to general procedure B from commercially available starting material with a reaction time of 18 h. ^1^H NMR yield (based on the integration of peaks at 6.13 and 5.63 ppm): 56%.

### 1-Ethyl-2-vinylbenzene^41^ (14)

The title compound was prepared according to general procedure B from commercially available starting material with a reaction time of 18 h. ^1^H NMR yield (based on the integration of peaks at 6.13 and 5.63 ppm): 60%.

### *tert*-Butyl (4-Vinylphenyl)carbamate^42^ (15)

The title compound was prepared according to general procedure B from commercially available starting material with a reaction time of 20 h. ^1^H NMR yield (based on the integration of peaks at 6.13 and 5.68 ppm): 40%.

### 4-Vinylphenol^43^ (16)

The title compound was prepared according to general procedure B from a commercially available starting material with a reaction time of 18 h. ^1^H NMR yield (based on the integration of peaks at 6.15 and 5.61 ppm): 15%.

### 1-Methyl-6-vinyl-1*H*-indazole (17)

The title compound was prepared according to general procedure C from commercially available starting material with a reaction time of 20 h, following Workup Procedure 1. The crude residue was filtered over a short plug of silica using pentane to give the product as a white solid. ^1^H NMR yield (based on the integration of peaks at 6.09 and 5.86 ppm): 66%. Isolated yield: (8 mg, 25%). Decomposition observed over time, and upon applying high vacuum. The title compound was also prepared according to general procedure D from commercially available starting material with a reaction time of 20 h, following Workup Procedure 1. ^1^H NMR yield (based on the integration of peaks at 6.09 and 5.84 ppm): 56%. ^1^H NMR (400 MHz, CDCl_3_) δ 7.93 (d, *J* = 1.0 Hz, 1H), 7.66 (dt, *J* = 8.4, 0.6 Hz, 1H), 7.35–7.27 (m, 2H), 6.91–6.80 (m, 1H), 5.86 (dd, *J* = 17.5, 0.8 Hz, 1H), 5.34 (dd, *J* = 10.9, 0.8 Hz, 1H), 4.07 (s, 3H). ^13^C{1H} NMR (101 MHz, CDCl_3_) δ 140.5, 137.3, 136.1, 132.8, 123.8, 121.1, 118.9, 114.7,107.1, 35.6. HRMS (ESI/q-TOF): *m*/*z* [M + H]^+^ calcd. for C_10_H_10_N_2_+H^+^: 159.0922; found: 159.0915.

### 1-Methoxy-6-vinylisoquinoline (18)

The title compound was prepared according to general procedure C from commercially available starting material with a reaction time of 20 h, following Workup Procedure 1. The crude residue was filtered over a short plug of silica using pentane to give the product as a colorless oil. ^1^H NMR yield (based on the integration of peaks at 6.09 and 5.92 ppm): 68%. Isolated yield: (25 mg, 68%, 91% purity). ^1^H NMR (400 MHz, CDCl_3_) δ 8.21–8.14 (m, 1H), 7.98 (d, *J* = 5.9 Hz, 1H), 7.64 (d, *J* = 7.2 Hz, 2H), 7.17 (dd, *J* = 5.9, 0.9 Hz, 1H), 6.86 (dd, *J* = 17.6, 10.9 Hz, 1H), 5.93 (dd, *J* = 17.6, 0.8 Hz, 1H), 5.42 (dd, *J* = 10.9, 0.8 Hz, 1H), 4.13 (s, 3H). ^13^C{1H} NMR (150 MHz, CDCl3) δ 161.0, 140.3, 139.5, 138.3, 136.5, 124.5, 124.3, 124.2, 119.3, 116.4, 115.1, 53.8. HRMS (ESI/Orbitrap): *m*/*z* [M + H]^+^ calcd. for C_12_H_11_NO+H^+^: 186.0919; found: 186.0913.

### 3-Vinylquinoline^44^ (19)

The title compound was prepared according to general procedure C from commercially available starting material with a reaction time of 20 h, following Workup Procedure 1. The crude residue was filtered over a short plug of silica using pentane to give the product as pale-yellow oil. Isolated yield: (18 mg, 57%). ^1^H NMR (400 MHz, CDCl_3_) δ 9.03 (d, *J* = 2.2 Hz, 1H), 8.11–8.05 (m, 2H), 7.81 (dd, *J* = 8.2, 1.4 Hz, 1H), 7.68 (ddd, *J* = 8.5, 6.9, 1.5 Hz, 1H), 7.54 (ddd, *J* = 8.0, 6.8, 1.2 Hz, 1H), 6.88 (dd, *J* = 17.7, 11.0 Hz, 1H), 5.99 (dd, *J* = 17.8, 0.6 Hz, 1H), 5.47 (dd, *J* = 11.0, 0.6 Hz, 1H). ^13^C{1H} NMR (101 MHz, CDCl_3_) δ 149.3, 147.8, 133.9, 132.6, 130.5, 129.4, 129.4, 128.1, 128.0, 127.1, 116.5.

### 3-Vinylpyridine^39^ (20)

The title compound was prepared according to general procedure B from a commercially available starting material with a reaction time of 40 h. ^1^H NMR yield (based on the integration of peaks at 6.09 and 5.49 ppm): 57%.

### 4-Vinylbenzofuran (21)

The title compound was prepared according to general procedure C from commercially available starting material with a reaction time of 20 h, following Workup Procedure 1. The crude residue was filtered over a short plug of silica by using pentane to give the product as a colorless oil. ^1^H NMR yield (based on the integration of peaks at 6.09 and 5.88 ppm): 74%. Isolated yield: (18 mg, 63%). ^1^H NMR (400 MHz, CDCl_3_) δ 7.66 (d, *J* = 2.3 Hz, 1H), 7.43 (dt, *J* = 8.1, 1.0 Hz, 1H), 7.35 (dt, *J* = 7.5, 0.8 Hz, 1H), 7.31–7.24 (m, 1H), 7.07–6.99 (m, 1H), 6.97 (dd, *J* = 2.2, 1.0 Hz, 1H), 5.88 (dd, *J* = 17.7, 1.1 Hz, 1H), 5.42 (dd, *J* = 11.1, 1.1 Hz, 1H). ^13^C{1H} NMR (101 MHz, CDCl_3_) δ: 155.4, 145.3, 134.6, 131.2, 125.7, 124.4, 120.2, 115.8, 110.8, 105.3. HRMS (ESI/Orbitrap): *m*/*z* [M + H]^+^ calcd. for C_10_H_8_O+H^+^: 145.0653; found: 145.0647.

### 5-Vinylbenzo[*b*]thiophene^39^ (22)

The title compound was prepared according to general procedure C from commercially available starting material with a reaction time of 20 h, following Workup Procedure 1. The crude residue was filtered over a short plug of silica using pentane to give the product as a yellow solid. ^1^H NMR yield (based on the integration of peaks at 6.09 and 5.80 ppm): 70%. Isolated yield: (22 mg, 69%).^1^H NMR (400 MHz, CDCl_3_) δ 7.86–7.78 (m, 2H), 7.52–7.41 (m, 2H), 7.32 (dd, *J* = 5.4, 0.8 Hz, 1H), 6.84 (dd, *J* = 17.6, 10.9 Hz, 1H), 5.81 (dd, *J* = 17.6, 0.9 Hz, 1H), 5.28 (dd, *J* = 10.9, 0.9 Hz, 1H). ^13^C{1H} NMR (101 MHz, CDCl_3_) δ: 140.1, 139.3, 137.1, 134.2, 127.0, 124.1, 122.6, 122.3, 121.8, 113.6.

### 2-Vinyl-6-fluoronaphthalene (23)

The title compound was prepared according to general procedure C from a commercially available starting material with a reaction time of 18 h, following Workup Procedure 1. The crude residue was filtered over a short plug of silica using pentane to give the product as a colorless oil. ^1^H NMR yield (based on the integration of peaks at 6.09 and 5.86 ppm): 77%. Isolated yield: (23 mg, 67%).^1^H NMR (400 MHz, CDCl_3_) δ 7.78 (dd, *J* = 9.0, 5.6 Hz, 1H), 7.73 (dd, *J* = 5.0, 3.5 Hz, 2H), 7.65 (dd, *J* = 8.6, 1.7 Hz, 1H), 7.41 (dd, *J* = 9.8, 2.6 Hz, 1H),7.29–7.19 (m, 1H), 6.86 (dd, *J* = 17.6, 10.9 Hz, 1H), 5.85 (d, *J* = 17.6 Hz, 1H), 5.33 (d, *J* = 10.6 Hz, 1H). ^13^C{1H} NMR (151 MHz, CDCl_3_) δ 161.6, 160.0, 136.7, 134.5, 133.9, 130.5 (d, *J* = 9.2 Hz), 127.6 (d, *J* = 5.3 Hz), 126.3, 124.4, 116.8 (d, *J* = 25.4 Hz), 114.3, 111.1 (d, *J* = 20.5 Hz). ^19^F-NMR (376 MHz, CDCl_3_) δ −114.6.

## Data Availability

The data underlying this study are available in the published article and its Supporting Information.

## Supporting Information Available

Experimental details, characterization data and spectra of novel compounds as well as spectra for NMR-quantification of literature known volatile compounds. (PDF)
