# Supplementary material for: Tetraethylammonium Salts as Solid, Easy to Handle Ethylene Precursors and Their Application in Mizoroki–Heck Coupling
Source: J Org Chem. 2024 Mar 11;89(7):5126–33. doi: 10.1021/acs.joc.3c02867 (PMC11002924; doi:10.1021/acs.joc.3c02867)
Supplement: Supplementary file 1 — jo3c02867_si_001.pdf [file jo3c02867_si_001.pdf]

Supporting Information

Tetraethylammonium salts as solid, easy to handle ethylene precursors and their application in Mizoroki-Heck coupling.

Eleni Papaplioura [a], Maëva Mercier [b], Michael E. Muratore [c], Tobias Biberger [c], Soufyan Jerhaoui [b]*, Michael Schnürch [a]*

[a]Institute of Applied Synthetic Chemistry, TU Wien, Getreidemarkt 9/163, 1060 Wien, Austria, [b] Discovery Science, Discovery Chemistry BE, Janssen Pharmaceutica N.V., Turnhoutseweg 30, 2340 Beerse, Belgium [c]Boehringer Ingelheim RCV GmbH & Co. KG, A-1121 Vienna.[a]

E-mail: michael.schnuerch@tuwien.ac.at, SJerhao1@its.jnj.com

Contents

List of Abrreviations S4
General Experimental Details S4
General Experimental Procedures S5
Reaction Optimization Screening S5
Substrate Scope Screening S6
Characterization data for all synthetic compounds S13
Substrate Scope S13
Unsuccessful Substrates S20
NMR-Spectra of isolated compounds S27
NMR-Spectra of volatile compounds for quantification S36
References S53

List of Tables

Table S1: Base screening: S7
Table S2: Quaternary ammonium salt screening: S8
Table S3: Catalyst screening, temperature, reaction times and loadings: S9
Table S4: Solvent screening: S10
Table S5: Polymerization inhibitors screening: S10
Table S6: Catalyst equivalents screening: S10
Table S7: Control experiments screening: S10
Table S8: NMR quantification calculations of volatile products 3-16 & 20: S11

List of Figures

Figure S1: ^{1}H-NMR used as an example for quantification. S5
Figure S2: ^{1}H-NMR (400 MHz, $CDCl_3$) and ^{13}C{1H} NMR (101 MHz, $CDCl_3$) of 2-vInyl-6-methoxynapththalene (2). S28
Figure S3: ^{1}H-NMR (400 MHz, $CDCl_3$) and ^{13}C{1H} NMR (101 MHz, $CDCl_3$) of 1-methyl-6-vinyl-1H-indazole (17) S29
Figure S4: ^{1}H-NMR (400 MHz, $CDCl_3$) and ^{13}C{1H} NMR (101 MHz, $CDCl_3$) of 1-methoxy-6-vinylquinoline (18) S30
Figure S5: ^{1}H-NMR (400 MHz, $CDCl_3$) and ^{13}C{1H} NMR (101 MHz, $CDCl_3$) of 3-vinylquinoline (19). S31
Figure S6: ^{1}H-NMR (400 MHz, $CDCl_3$) and ^{13}C{1H} NMR (101 MHz, $CDCl_3$) of 4-vinylbenzofuran (21) S32
Figure S7: ^{1}H-NMR (400 MHz, $CDCl_3$) and ^{13}C{1H} NMR (101 MHz, $CDCl_3$) of 5-vinyl-benzo[b]thiophene (22) S33
Figure S8: ^{1}H-NMR (400 MHz, $CDCl_3$) ^{13}C{1H} NMR (101 MHz, $CDCl_3$) and ^{19}F-NMR (376 MHz, $CDCl_3$) of 2-vinyl-6-fluoronapthalene (23). S35

Figure S9: ^{1}H-NMR (400 MHz, C_7D_8) used for quantification of styrene (3) when X=Br. S37
Figure S10: ^{1}H-NMR (400 MHz, C_7D_8) used for quantification of styrene (3) when X=I. S38
Figure S11: ^{1}H-NMR (400 MHz, C_7D_8) used for quantification of 1-(trifluoromethyl)-3-vinylbenzene (4). S39
Figure S12: ^{1}H-NMR (400 MHz, C_7D_8) used for quantification of 1-(trifluoromethyl)-4-vinylbenzene (5). S40
Figure S13: ^{1}H-NMR (400 MHz, C_7D_8) used for quantification of 1-(trifluoromethyl)-2-vinylbenzene (6). S41
Figure S14: ^{1}H-NMR (400 MHz, C_7D_8) used for quantification of 4-vinylbenzoic acid (7). S42
Figure S15: ^{1}H-NMR (400 MHz, C_7D_8) used for quantification of 1-methoxy-3-vinylbenzene (8). S43
Figure S16: ^{1}H-NMR (400 MHz, C_7D_8) used for quantification of 1-methoxy-4-vinylbenzene (9). S44
Figure S17: ^{1}H-NMR (400 MHz, C_7D_8) used for quantification of 1-methoxy-2-vinylbenzene (10). S45
Figure S18: ^{1}H-NMR (400 MHz, C_7D_8) used for quantification of N,N-dimethyl-4-vinylaniline (11). S46
Figure S19: ^{1}H-NMR (400 MHz, C_7D_8) used for quantification of 1-ethyl-3-vinylbenzene (12). S47
Figure S20: ^{1}H-NMR (400 MHz, C_7D_8) used for quantification of 1-ethyl-4-vinylbenzene (13). S48
Figure S21: ^{1}H-NMR (400 MHz, C_7D_8) used for quantification of 1-ethyl-2-vinylbenzene (14). S49
Figure S22: ^{1}H-NMR (400 MHz, C_7D_8) used for quantification of tert-butyl (4-vinylphenyl)carbamate (15). S50
Figure S23: ^{1}H-NMR (400 MHz, C_7D_8) used for quantification of 4-vinylphenol (16). S51
Figure S24: ^{1}H-NMR (400 MHz, C_7D_8) used for quantification of 3-vinylpyridine (20). S52

List of Abrreviations

Abbreviation	Definition
TEA	Triethylamine
TMB	1,3,5-Trimethoxybenzene
SM	Starting material
IS	Internal standard

General Experimental Details

Unless noted otherwise, reactants and reagents were purchased from commercial suppliers and used without further purification. The 4 mL brown-glass vials were sealed with Wheaton® screw caps containing a PTFE faced 14B styrene-butadiene rubber liner. All reactions were magnetically stirred and heated in a metallic reaction block. All reaction temperatures refer to external temperatures.

NMR spectra were recorded in $CDCl_3$ or d_8-toluene on a Brucker Avance UltraShield (400 MHz) or Avance III HD 600 (600 MHz) spectrometer and chemical shifts (δ) are reported in ppm, using Me_4Si as internal standard. Coupling constants (*J*) are given in Hertz (Hz) and multiplicities are assigned by the following abbreviations = singlet, d = doublet, t = triplet, q = quartet, and m = multiplet.

Thin Layer Chromatography (TLC) analysis was performed on precoated aluminum-backed unmodified plates (Silica gel 60 F_{254}, Merck). Compounds were visualized under UV light.

Flash column chromatography was performed using either Merck silica gel 60 (40 µm – 63 µm) by hand column or purification was performed using a TELEDYNE ISCO COMBI FLASH® COMPANION® NextGen300+ flash chromatography system with UV-Vis detector (diode array) with Macherey-Nagel Chromabond RS silica gel columns.

GC-MS analysis was performed on Thermo Finnigan Focus GC/DSQ II with a standard capillary column RXi-5Sil MS column (30 m, 0.25 mm ID, 0.25 µm df) using the following standardized temperature programs: **Method A:** 2 min at 100 °C, 35 °C/min until 300 °C, 4 min at 300 °C and **Method B:** 2.5 min at 40 °C, 12 °C/min until 220 °C, 2.5 min at 220 °C.

HR-MS data were recorded using a Thermo Scientific Orbitrap Elite hybrid ion trap/orbitrap spectrometer system with an Ultimate 3000 series LPG-3400XRS pump system. Mass calibration was performed using the Pierce LTQ Velos ESI positive ion calibration solution from Thermo Scientific (lot PF200011, product no. 88323).

General Experimental Procedures

Reaction Optimization Screening

General Procedure A

In a 4-mL brown vial equipped with a magnetic stirring bar and a Wheaton® screw cap, were added 2-bromo-6-methoxynapthalene (**1**) (48.4 mg, 0.2 mmol, 1 equiv.), the respective ammonium salt (8 equiv.), the base (8 equiv.), and the catalyst (x mol %). Subsequently, solvent (0.2 M) was added *via* syringe, and the reaction mixture was heated to 120 °C, or to 100 °C and 80 °C, depending on the corresponding solvent in a metallic block for 18-36 h respectively.

<u>Sample preparation for ^{1}H-NMR quantification:</u>

The reaction mixture was cooled down to room temperature and filtered over celite in a syringe filter. The filter cake was washed with EtOAc and the volatiles removed in *vacuo*. 1,3,5-Trimethoxybenzene (0.2 mmol, 1 equiv.) was added, and the crude mixture was dissolved in 0.5 mL $CDCl_3$ and transferred to an NMR tube. The recorded spectra were processed by MestReNova v14 software.

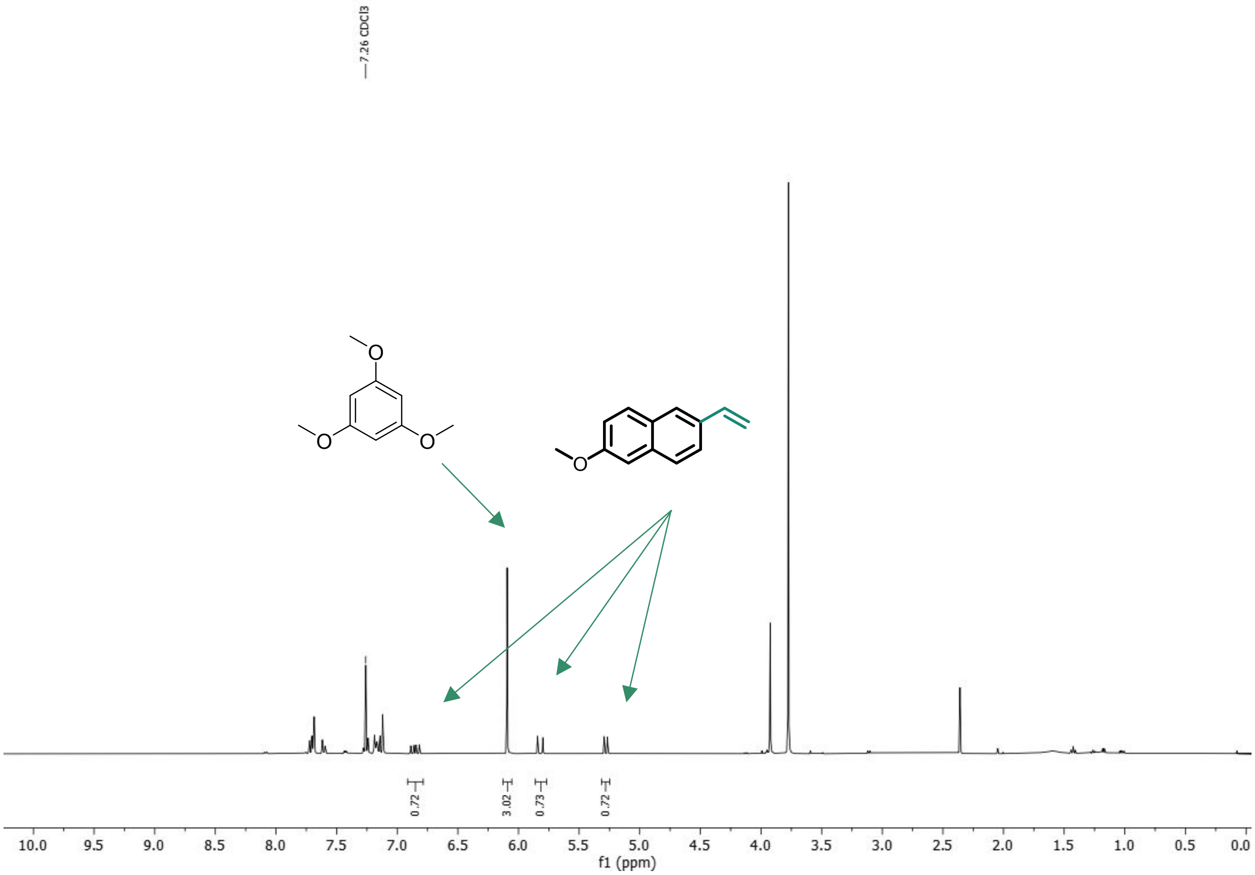

Figure S1: ^{1}H-NMR used as an example for quantification.

Substrate Scope Screening

General Procedure B

In a 4-mL brown vial equipped with a magnetic stirring bar and a Wheaton® screw cap, were added the starting material (0.2 mmol, 1 equiv.), Et_4NBr (8 equiv.), KO*t*Bu (8 equiv.), and PdRuPhos G3 (10 mol %). Subsequently, d_8-toluene (0.2 M) was added *via* syringe, and the reaction mixture was heated to 100 °C, in a metallic block for 18-36 h.

Sample preparation for ^{1}H-NMR quantification:

The reaction mixture was cooled down to room temperature and filtered in a syringe filter. The filter cake was washed with d_8-toluene and 1,3,5-trimethoxybenzene (0.2 mmol, 1 equiv.) was added to the crude reaction mixture. In an NMR tube were transferred 0.5 mL of the reaction solution and the recorded spectra were processed by MestReNova v14.

General Procedure C

In a 4-mL brown vial equipped with a magnetic stirring bar and a Wheaton® screw cap, were added the starting material (0.2 mmol, 1 equiv.), Et_4NBr (8 equiv.), KO*t*Bu (8 equiv.), and PdRuPhos G3 (10 mol %). Subsequently, toluene (0.2 M) was added *via* syringe, and the reaction mixture was heated to 100 °C, in a metallic block for 18-36 h.

- Workup Procedure 1: The reaction mixture was cooled down to room temperature and filtered in a syringe filter. The filter cake was washed with EtOAc and the volatiles removed in *vacuo*.
- Workup Procedure 2: The reaction mixture was cooled down to room temperature and filtered in a syringe filter and toluene was removed in *vacuo*. The crude mixture was redissolved in DCM and water was added (HCl 1M was added for neutralization). The aqueous phase was extracted with DCM (x 5), the combined organic extracts dried over Na_2SO_4 and the volatiles removed in *vacuo*.

Sample preparation for ^{1}H-NMR quantification:

1,3,5-Trimethoxybenzene (0.2 mmol, 1 equiv.) was added, and the crude mixture was dissolved in 0.5 mL $CDCl_3$ and transferred to an NMR tube. The recorded spectra were processed by MestReNova v14 software. The crude residue was purified either *via* manual column chromatography using unmodified silica or using an automated purification system.

General Procedure D

In a 4-mL brown vial equipped with a magnetic stirring bar and a Wheaton® screw cap, were added the starting material (0.2 mmol, 1 equiv.), Et_4NBr (8 equiv.), KO*t*Bu (8 equiv.), and PdXPhos G (10 mol %). Subsequently, toluene (0.2 M) was added *via* syringe, and the reaction mixture was heated to 100 °C, in a metallic block for 18-20 h.

- Workup Procedure 1: The reaction mixture was cooled down to room temperature and filtered in a syringe filter. The filter cake was washed with EtOAc and the volatiles removed in *vacuo*.

Sample preparation for ^{1}H-NMR quantification:

1,3,5-Trimethoxybenzene (0.2 mmol, 1 equiv.) was added, and the crude mixture was dissolved in 0.5 mL $CDCl_3$ and transferred to an NMR tube. The recorded spectra were processed by MestReNova v14

software. The crude residue purified either *via* manual column chromatography using unmodified silica or using an automated purification system.

General Procedure E

In a 4-mL brown vial equipped with a magnetic stirring bar and a Wheaton® screw cap, were added the starting material (0.2 mmol, 1 equiv.), Et_4NBr (8 equiv.), KO*t*Bu (8 equiv.), and PdXPhos G4 (10 mol %). Subsequently, d_8-toluene (0.2 M) was added *via* syringe, and the reaction mixture was heated to 100 °C, in a metallic block for 18 h.

<u>Sample preparation for ^{1}H-NMR quantification:</u>

The reaction mixture was cooled down to room temperature and filtered in a syringe filter. The filter cake was washed with d_8-toluene and 1,3,5-trimethoxybenzene (0.2 mmol, 1 equiv.) was added to the crude reaction mixture. In an NMR tube were transferred 0.5 mL of the reaction solution and the recorded spectra were processed by MestReNova v14.

Optimization Screening

The reaction below was chosen as the model reaction and yields were determined by quant. ^{1}H-NMR spectroscopy for the crude reaction mixtures using 1,3,5- trimethoxybenzene as internal standard. We examined the following parameters: quaternary ammonium salt, base, Pd source/ loading, solvent, temperature, reaction time.

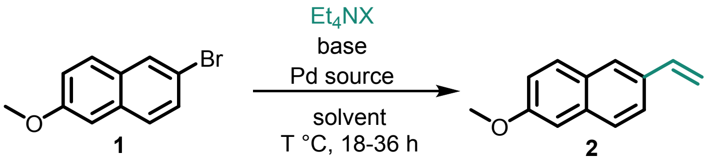

Reactions were performed according to the general procedure A using Et_4NBr (1.6 mmol, 8 equiv.) as the olefin surrogate, the respective base (1.6 mmol, 8 equiv.), PdXPhos G4 (5 or 10 mol %) as the catalyst and toluene (1 mL, 0.2 M) at 120 °C for 18 h.

Table S1: Base screening:

Entry	Ammonium salt	Solvent	Base	Pd source	Yield (%)
1	Et_4NBr	Toluene	KOH	PdXPhos G4 (10 mol %)	20
2	Et_4NBr	Toluene	KOH	PdXPhos G4 (5 mol %)	31
3	Et_4NBr	Toluene	DBU	PdXPhos G4 (10 mol %)	-
4	Et_4NBr	Toluene	KO*t*Bu	PdXPhos G4 (5 mol %)	44
5	Et_4NBr	Toluene	KO*t*Bu	PdXPhos G4 (10 mol %)	54
6	Et_4NBr	Toluene	KOTMS	PdXPhos G4 (10 mol %)	48

Reactions were performed according to the general procedure A using the respective ammonium salt (1.6 mmol, 8 equiv.) as the olefin surrogate, KO*t*Bu as the base (1.6 mmol, 8 equiv.), PdXPhos G4 (10 mol %) as the catalyst and toluene (1 mL, 0.2 M) at 120 °C for 18 h.

Table S2: Quaternary ammonium salt screening:

Entry	Ammonium salt	Solvent	Base	Pd source	Yield (%)
1	Et_4NCl	Toluene	KO*t*Bu	PdXPhos G4	53
2	Et_4NI	Toluene	KO*t*Bu	PdXPhos G4	49
3	Et_4NPF_6	Toluene	KO*t*Bu	PdXPhos G4	48
4	Et_4NBF_4	Toluene	KO*t*Bu	PdXPhos G4	44
5	$PhEt_3NI$	Toluene	KO*t*Bu	PdXPhos G4	8

Reactions were performed according to the general procedure A using Et_4NBr (1.6 mmol, 8 equiv.) as the olefin surrogate, KO*t*Bu as the base (1.6 mmol, 8 equiv.), the respective catalyst (10 mol %) and toluene (1 mL, 0.2 M) at 120 °C.

Table S3: Catalyst screening, temperature, reaction times and loadings:

Entry	Ammonium salt	Solvent	Base	Pd source	Temp. °C	Time	Yield (%)
1	Et_4NBr	Toluene	KO*t*Bu	$PdOAc_2$ /(o-Tol)$_3$P (40 mol %)	100	18	trace
2	Et_4NBr	Toluene	KO*t*Bu	$Pd(OAc)_2$ /1 wt % H_2O	120	20	31
3	Et_4NBr	Toluene	KO*t*Bu	$Pd(OAc)_2$ /1 wt % H_2O	120	72	47
4	Et_4NBr	Toluene	KO*t*Bu	$Pd(OAc)_2$/ PPh_3 (20 mol %)	120	20	29
5	Et_4NBr	Toluene	KO*t*Bu	$Pd(OAc)_2$/ PCy_3 (20 mol %)	120	20	25
6	Et_4NBr	Toluene	KO*t*Bu	$PdCl_2$	120	20	0
7	Et_4NBr	Toluene	KO*t*Bu	$PdCl_2$ / PPh_3 (20 mol %)	120	20	43
8	Et_4NBr	Toluene	KO*t*Bu	$PdCl_2(PPh_3)_2$	120	20	18
9	Et_4NBr	Toluene	KO*t*Bu	$Pd_2(dba)_3$	120	20	25
10	Et_4NBr	Toluene	KO*t*Bu	$Pd(PPh_3)_4$	120	20	28
11	Et_4NBr	Toluene	KO*t*Bu	PdXPhos G4	120	18	54
12	Et_4NBr	Toluene	KO*t*Bu	PdXPhos G4	100	18	77
13	Et_4NBr	Toluene	KO*t*Bu	PdXPhos G4/ 1 wt % H_2O	120	20	44
14	Et_4NBr	Toluene	KO*t*Bu	PdSPhos G3	100	18	73
15	Et_4NBr	Toluene	KO*t*Bu	PdBINAP G4	100	18	27
16	Et_4NBr	Toluene	KO*t*Bu	Pd CataCXium A G3	100	18	53
17	Et_4NBr	Toluene	KO*t*Bu	PdBrettPhos G4	100	18	26
18	Et_4NBr	Toluene	KO*t*Bu	PdCPhos G3	100	18	53
19	Et_4NBr	Toluene	KO*t*Bu	$Pd(dppf)Cl_2$	100	18	13
20	Et_4NBr	Toluene	KO*t*Bu	$(Ataphos)_2PdCl_2$	100	18	-
21	Et_4NBr	Toluene	KO*t*Bu	PdXanpthos G3	100	18	27
22	Et_4NBr	Toluene	KO*t*Bu	PdPEPPSI	100	18	20
23	Et_4NBr	Toluene	KO*t*Bu	PdRuPhos G3	100	18	82
24	Et_4NBr	Toluene	KO*t*Bu	PdJosiphos SL-J009-1 G3	100	18	79
25	Et_4NBr	Toluene	KO*t*Bu	PdXPhos G4 (10 mol %)	120	6	50
26	Et_4NBr	Toluene	KO*t*Bu	PdXPhos G4 (5 mol %)	120	9	48

Reactions were performed according to the general procedure A using Et_4NBr (1.6 mmol, 8 equiv.) as the olefin surrogate, KO*t*Bu as the base (1.6 mmol, 8 equiv.), PdXPhos G4 (10 mol %) as the catalyst, and the respective solvent (1 mL, 0.2 M) for 20-48 h.

Table S4: Solvent screening:

Entry	Ammonium salt	Solvent	Base	Pd source	Temp. °C	Time	Yield (%)
1	Et_4NBr	Me-THF	KO*t*Bu	PdXPhos G4	80	20	-
2	Et_4NBr	Me-THF	KO*t*Bu	PdXPhos G4	80	48	-
3	Et_4NBr	CPME	KO*t*Bu	PdXPhos G4	100	20	70
4	Et_4NBr	CPME	KO*t*Bu	PdXPhos G4	100	48	71

Reactions were performed according to the general procedure A using Et_4NBr (1.6 mmol, 8 equiv.) as the olefin surrogate, KO*t*Bu as the base (1.6 mmol, 8 equiv.), PdXPhos G4 (10 mol %) as the catalyst, the respective polymerization inhibitor and toluene as the solvent (1 mL, 0.2 M) at 100-120 °C for 20 h.

Table S5: Polymerization inhibitors screening:

Entry	Ammonium salt	Solvent	Base	Pd source	Temp. °C	Yield (%)
1	Et_4NBr	Toluene	KO*t*Bu	PdXPhos G4/10 mol % cod	120	55
2	Et_4NBr	Toluene	KO*t*Bu	PdXPhos G4/BHT (1 equiv.)	100	75
3	Et_4NBr	Toluene	KO*t*Bu	PdXPhos G4/ 1 wt % 4-*tert*-butylcatechol	100	75

Reactions were performed according to the general procedure A using Et_4NBr (1.6 mmol, 8 equiv.) as the olefin surrogate, KO*t*Bu as the base (1.6 mmol, 8 equiv.), PdXPhos G4 (x mol %) as the catalyst, and toluene as the solvent (1 mL, 0.2 M) at 120 °C for 20 h.

Table S6: Catalyst equivalents screening:

Entry	Ammonium salt	Solvent	Base	Pd source	Yield (%)
1	Et_4NBr	Toluene	KO*t*Bu	PdXPhos G4 (5 mol %)	44
2	Et_4NBr	Toluene	KO*t*Bu	PdXPhos G4 (15 mol %)	46
3	Et_4NBr	Toluene	KO*t*Bu	PdXPhos G4 (20 mol %)	43

Table S7: Control experiments screening:

Entry	Ammonium salt	Solvent	Base	Pd source	Additive	Yield (%)
1	Et_4NBr	Toluene	KO*t*Bu	PdRuPhos G3	styrene (1 equiv.)	80
2	Et_4NBr	Toluene	KO*t*Bu	PdXPhos G4	styrene (1 equiv.)	44
3	Et_4NBr	Toluene	KO*t*Bu	PdRuPhos G3 (10 mol %)	Et_3N	83

Table S8: NMR quantification calculations of volatile products 3-16 & 20:

Entry	Product	SM (mmol)	IS (mmol)	Integral of one vinylic proton (product)	Integral of aromatic protons IS (three protons)	Yield (%)
1	**3** (X=Br)	0.2	0.2	0.67	3.00	67
2	**3** (X=I)	0.2	0.2	0.45	3.00	45
3	**4**	0.2	0.190	0.75	3.00	71
4	**5**	0.2	0.193	0.43	3.00	41
5	**6**	0.2	0.2	0.32	3.00	32
6	**7**	0.2	0.2	0.20	3.00	20
7	**8**	0.2	0.196	0.52	3.00	51
8	**9**	0.2	0.193	0.52	3.00	50
9	**10**	0.2	0.190	0.33	3.00	31
10	**11**	0.21	0.188	0.23	3.00	21
11	**12**	0.2	0.188	0.46	3.00	43
12	**13**	0.2	0.21	0.54	3.00	56
13	**14**	0.2	0.2	0.60	3.00	60
14	**15**	0.2	0.2	0.40	3.00	40
15	**16**	0.2	0.2	0.15	3.00	15
16	**20**	0.2	0.2	0.57	3.00	57

CO-Ware reactor

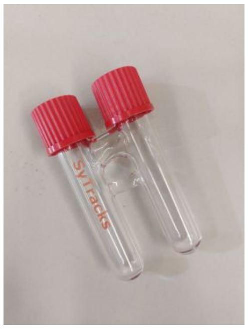

In a two-chamber reactor (CO-Ware) equipped with two stirring bars at each chamber were added: **Chamber 1**: Et_4NBr (8 equiv.), KOH (8 equiv.) and toluene (1 mL), **Chamber 2**: Starting material (0.2 mmol, 1 equiv.), KO*t*Bu (8 equiv.),PdXPhos G4 (5 mol%) and toluene (0.2 M, 1 mL), and the reaction mixture was heated to 120 °C in a metallic block for 20 h. No product formation was observed.

Characterization data for all synthetic compounds

All synthesized compounds are described in the literature, except for **18**, **19**, **22** and **25**. In the case of already known compounds, the spectra were in agreement with literature values.

Substrate Scope

Styrene (3)[1] [CAS 100-42-5]

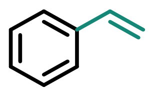

The title compound was prepared according to general procedure B from commercially available starting materials with a reaction time of 20 h. From bromobenzene **[CAS 108-86-1]:** ^{1}H-NMR yield (based on the integration of peaks at 6.10 and 5.59 ppm): 67 %, and from iodobenzene **[CAS 591-50-4]:** ^{1}H-NMR yield (based on the integration of peaks at 6.10 and 5.59 ppm): 45 %

2-Vinyl-6-methoxynaphthalene (2)[2] [CAS: 63444-51-9]

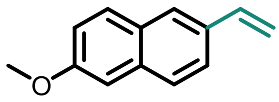

The title compound was prepared according to general procedure C from commercially available starting material with a reaction time of 20 h, following Workup Procedure 1. The crude residue was filtered over a short plug of silica using pentane to give the product as a white solid. ^{1}H-NMR yield (based on the integration of peaks at 6.09 and 5.80 ppm): 82 %. Isolated yield: (30 mg, 81 %).

^{1}H-NMR (400 MHz, $CDCl_3$) δ 7.77 – 7.67 (m, 3H), 7.60 (dd, *J* = 8.8, 1.6 Hz, 1H), 7.19 – 7.09 (m, 2H), 6.85 (dd, *J* = 17.6, 10.8 Hz, 1H), 5.82 (dd, *J* = 17.6, 0.9 Hz, 1H), 5.28 (dd, *J* = 10.8, 0.9 Hz, 1H), 3.92 (s, 3H).

^{13}C{1H} NMR (101 MHz, $CDCl_3$) δ 157.9, 137.1, 134.5, 133.1, 129.7, 129.1, 127.1, 126.3, 123.9, 119.1, 113.2, 106.0, 55.5.

Compound (**2**) was also prepared on a 0.4 mmol scale as described above: ^{1}H-NMR yield (based on the integration of peaks at 6.09 and 5.80 ppm): 71 %, on a 0.6 mmol scale: ^{1}H-NMR yield (based on the integration of peaks at 6.09 and 5.80 ppm): 65 %, and on a 1 mmol scale (1 equiv.) using Et_4NBr (8 equiv.), KO*t*Bu (8 equiv.), and PdRuPhos G3 (10 mol %).: Isolated yield: (68 mg, 37 %, 0.148 mmol).

1-(Trifluoromethyl)-3-vinylbenzene (4)[3] [CAS: 402-24-4]

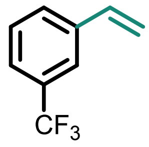

The title compound was prepared according to general procedure B from commercially available starting material with a reaction time of 18 h. ^{1}H-NMR yield (based on the integration of peaks at 6.10 and 5.49 ppm): 71 %.

1-(Trifluoromethyl)-4-vinylbenzene (5)[3] [CAS: 402-50-6]

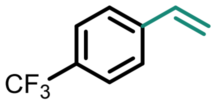

The title compound was prepared according to general procedure B from commercially available starting material with a reaction time of 18 h. ^{1}H-NMR yield (based on the integration of peaks at 6.13 and 5.47 ppm): 41 %.

The title compound was also prepared according to general procedure E from commercially available starting material with a reaction time of 18 h. ^{1}H-NMR yield (based on the integration of peaks at 6.13 and 5.47 ppm): 9 %.

1-(Trifluoromethyl)-2-vinylbenzene (6)[4] [CAS: 395-45-9]

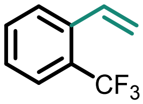

The title compound was prepared according to general procedure B from commercially available starting material with a reaction time of 40 h. ^{1}H-NMR yield (based on the integration of peaks at 6.13 and 5.48 ppm): 32 %.

4-Vinylbenzoic acid (7)[5] [CAS: 1075-49-6]

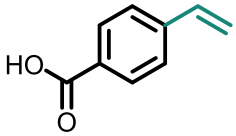

The title compound was prepared according to general procedure B from commercially available starting material with a reaction time of 18 h. ^{1}H-NMR yield (based on the integration of peaks at 6.13 and 5.57 ppm): 20 %.

The title compound was also prepared according to general procedure E from commercially available starting material with a reaction time of 18 h. ^{1}H-NMR yield (based on the integration of peaks at 6.13 and 5.57 ppm): 31 %.

1-Methoxy-3-vinylbenzene (8)[6] [CAS: 626-20-0]

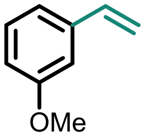

The title compound was prepared according to general procedure B from commercially available starting material with a reaction time of 18 h. ^{1}H-NMR yield (based on the integration of peaks at 6.13 and 5.78 ppm): 51 %.

The title compound was prepared according to general procedure E from commercially available starting material with a reaction time of 18 h. ^{1}H-NMR yield (based on the integration of peaks at 6.13 and 5.78 ppm): 51 %.

1-Methoxy-4-vinylbenzene (9)[7] [CAS: 637-69-4]

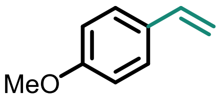

The title compound was prepared according to general procedure B from commercially available starting material with a reaction time of 40 h. ^{1}H-NMR yield (based on the integration of peaks at 6.13 and 5.54 ppm): 50 %.

1-Methoxy-2-vinylbenzene (10)[6] [CAS: 612-15-7]

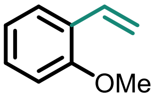

The title compound was prepared according to general procedure B from commercially available starting material with a reaction time of 40 h. ^{1}H-NMR yield (based on the integration of peaks at 6.13 and 5.71 ppm): 31 %.

***N*,*N*-Dimethyl-4-vinylaniline (11)[8] [CAS: 2039-80-7]**

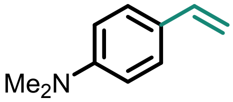

The title compound was prepared according to general procedure B from commercially available starting material with a reaction time of 18 h. ^{1}H-NMR yield (based on the integration of peaks at 6.13 and 5.57 ppm): 21 %.

1-Ethyl-3-vinylbenzene (12) [CAS: 7525-62-4] Spectral data were obtained from Enamine Ltd.

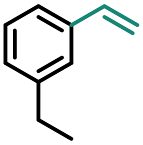

The title compound was prepared according to general procedure B from commercially available starting material with a reaction time of 18 h. ^{1}H-NMR yield (based on the integration of peaks at 6.13 and 5.67 ppm): 43 %.

1-Ethyl-4-vinylbenzene (13)[3] [CAS: 3454-07-7]

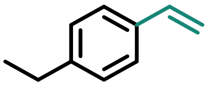

The title compound was prepared according to general procedure B from commercially available starting material with a reaction time of 18 h. ^{1}H-NMR yield (based on the integration of peaks at 6.13 and 5.63 ppm): 56 %.

1-Ethyl-2-vinylbenzene (14)[9] [CAS: 7564-63-8]

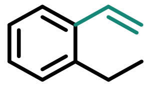

The title compound was prepared according to general procedure B from commercially available starting material with a reaction time of 18 h. ^{1}H-NMR yield (based on the integration of peaks at 6.13 and 5.54 ppm): 60 %.

***tert*-Butyl (4-vinylphenyl)carbamate (15)[10] [CAS: 57295-14-4]**

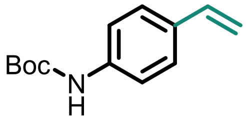

The title compound was prepared according to general procedure B from commercially available starting material with a reaction time of 20 h. ^{1}H-NMR yield (based on the integration of peaks at 6.13 and 5.68 ppm): 40 %.

4-Vinylphenol (16)[11] [CAS: 2628-17-3]

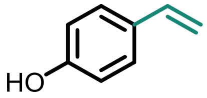

The title compound was prepared according to general procedure B from commercially available starting material with a reaction time of 18 h. ^{1}H-NMR yield (based on the integration of peaks at 6.15 and 5.61 ppm): 15 %.

1-Methyl-6-vinyl-1*H*-indazole (17)

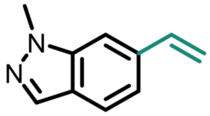

The title compound was prepared according to general procedure C from commercially available starting material with a reaction time of 20 h, following Workup Procedure 1. The crude residue was filtered over a short plug of silica using pentane to give the product as white solid. ^{1}H-NMR yield (based on the integration of peaks at 6.09 and 5.86 ppm): 66 %. Isolated yield: (8 mg, 25 %). Decomposition observed over time, and upon applying high vacuum.

The title compound was also prepared according to general procedure D from commercially available starting material with a reaction time of 20 h, following Workup Procedure 1. ^{1}H-NMR yield (based on the integration of peaks at 6.09 and 5.84 ppm): 56 %.

^{1}H-NMR (400 MHz, $CDCl_3$) δ 7.93 (d, J = 1.0 Hz, 1H), 7.66 (dt, J = 8.4, 0.6 Hz, 1H), 7.35 – 7.27 (m, 2H), 6.91 – 6.80 (m, 1H), 5.86 (dd, J = 17.5, 0.8 Hz, 1H), 5.34 (dd, J = 10.9, 0.8 Hz, 1H), 4.07 (s, 3H).

^{13}C{1H} NMR (101 MHz, $CDCl_3$) δ 140.5, 137.3, 136.1, 132.8, 123.8, 121.1, 118.9, 114.7,107.1, 35.6.

HRMS (ESI/q-TOF): *m/z* $[M+H]^+$ calcd. for $C_{10}H_{10}N_2+H^+$: 159.0922; found: 159.0915.

1-Methoxy-6-vinylisoquinoline (18)

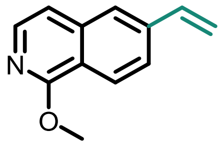

The title compound was prepared according to general procedure C from commercially available starting material with a reaction time of 20 h, following Workup Procedure 1. The crude residue was filtered over a short plug of silica using pentane to give the product as a colorless oil. ^{1}H-NMR yield (based on the integration of peaks at 6.09 and 5.92 ppm): 68 %. Isolated yield: (25 mg, 68 %, 91 % purity).

^{1}H-NMR (400 MHz, $CDCl_3$) δ 8.21 – 8.14 (m, 1H), 7.98 (d, *J* = 5.9 Hz, 1H), 7.64 (d, *J* = 7.2 Hz, 2H), 7.17 (dd, *J* = 5.9, 0.9 Hz, 1H), 6.86 (dd, *J* = 17.6, 10.9 Hz, 1H), 5.93 (dd, *J* = 17.6, 0.8 Hz, 1H), 5.42 (dd, *J* = 10.9, 0.8 Hz, 1H), 4.13 (s, 3H).

^{13}C{1H} NMR (150 MHz, CDCl3) δ 161.0, 140.3, 139.5, 138.3, 136.5, 124.5, 124.3, 124.2, 119.3, 116.4, 115.1, 53.8.

HRMS (ESI/Orbitrap): *m/z* $[M+H]^+$ calcd. for $C_{12}H_{11}NO+H^+$: 186.0919; found:186.0913.

3-Vinylquinoline (19)[12] [CAS: 67752-31-2]

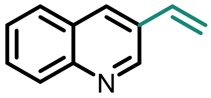

The title compound was prepared according to general procedure C from commercially available starting material with a reaction time of 20 h, following Workup Procedure 1. The crude residue was filtered over a short plug of silica using pentane to give the product as pale-yellow oil. Isolated yield: (18 mg, 57 %).

^{1}H-NMR (400 MHz, $CDCl_3$) δ 9.03 (d, *J* = 2.2 Hz, 1H), 8.11 – 8.05 (m, 2H), 7.81 (dd, *J* = 8.2, 1.4 Hz, 1H), 7.68 (ddd, *J* = 8.5, 6.9, 1.5 Hz, 1H), 7.54 (ddd, *J* = 8.0, 6.8, 1.2 Hz, 1H), 6.88 (dd, *J* = 17.7, 11.0 Hz, 1H), 5.99 (dd, *J* = 17.8, 0.6 Hz, 1H), 5.47 (dd, *J* = 11.0, 0.6 Hz, 1H).

^{13}C{1H} NMR (101 MHz, $CDCl_3$) δ 149.3, 147.8, 133.9, 132.6, 130.5, 129.4, 129.4, 128.1, 128.0, 127.1, 116.5.

3-Vinylpyridine (20)[7] [CAS: 1121-55-7]

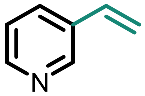

The title compound was prepared according to general procedure B from commercially available starting material with a reaction time of 40 h. ^{1}H-NMR yield (based on the integration of peaks at 6.13 and 5.53 ppm): 57 %.

4-Vinylbenzofuran (21) [CAS: 1337879-41-0]

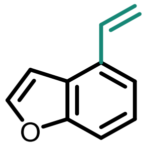

The title compound was prepared according to general procedure C from commercially available starting material with a reaction time of 20 h, following Workup Procedure 1. The crude residue was filtered over a short plug of silica using pentane to give the product as a colorless oil. ^{1}H-NMR yield (based on the integration of peaks at 6.09 and 5.88 ppm): 74 %. Isolated yield: (18 mg, 63 %).

^{1}H-NMR (400 MHz, $CDCl_3$) δ 7.66 (d, *J* = 2.3 Hz, 1H), 7.43 (dt, *J* = 8.1, 1.0 Hz, 1H), 7.35 (dt, *J* = 7.5, 0.8 Hz, 1H), 7.31 – 7.24 (m, 1H), 7.07 – 6.99 (m, 1H), 6.97 (dd, *J* = 2.2, 1.0 Hz, 1H), 5.88 (dd, *J* = 17.7, 1.1 Hz, 1H), 5.42 (dd, *J* = 11.1, 1.1 Hz, 1H).

^{13}C{1H} NMR (101 MHz, $CDCl_3$) δ 155.4, 145.3, 134.6, 131.2, 125.7, 124.4, 120.2, 115.8, 110.8, 105.3.

HRMS (ESI/Orbitrap): *m/z* $[M+H]^+$ calcd. for $C_{10}H_8O+H^+$: 145.0653; found: 145.0647.

5-Vinylbenzo[*b*]thiophene (22)[7] [CAS: 1033775-44-8]

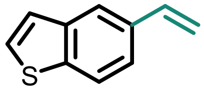

The title compound was prepared according to general procedure C from commercially available starting material with a reaction time of 20 h, following Workup Procedure 1. The crude residue was filtered over a short plug of silica using pentane to give the product as yellow solid. ^{1}H-NMR yield (based on the integration of peaks at 6.09 and 5.80 ppm): 70 %. Isolated yield: (22 mg, 69 %).

^{1}H-NMR (400 MHz, $CDCl_3$) δ 7.86 – 7.78 (m, 2H), 7.52 – 7.41 (m, 2H), 7.32 (dd, *J* = 5.4, 0.8 Hz, 1H), 6.84 (dd, *J* = 17.6, 10.9 Hz, 1H), 5.81 (dd, *J* = 17.6, 0.9 Hz, 1H), 5.28 (dd, *J* = 10.9, 0.9 Hz, 1H).

^{13}C{1H} NMR (101 MHz, $CDCl_3$) δ 140.1, 139.3, 137.1, 134.2, 127.0, 124.1, 122.6, 122.3, 121.8, 113.6.

2-Vinyl-6-fluoronaphthalene (23) [CAS: 1638155-81-3]

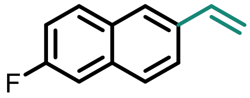

The title compound was prepared according to general procedure C from commercially available starting material with a reaction time of 18 h, following Workup Procedure 1. The crude residue was filtered over a short plug of silica using pentane to give the product as a colorless oil. ^{1}H-NMR yield (based on the integration of peaks at 6.09 and 5.86 ppm): 77 %. Isolated yield: (23 mg, 67 %).

---

^{1}H-NMR (400 MHz, $CDCl_3$) δ 7.78 (dd, *J* = 9.0, 5.6 Hz, 1H), 7.73 (dd, *J* = 5.0, 3.5 Hz, 2H), 7.65 (dd, *J* = 8.6, 1.7 Hz, 1H), 7.41 (dd, *J* = 9.8, 2.6 Hz, 1H),7.29 – 7.19 (m, 1H), 6.86 (dd, *J* = 17.6, 10.9 Hz, 1H), 5.85 (d, *J* = 17.6 Hz, 1H), 5.33 (d, *J* = 10.6 Hz, 1H).

^{13}C{1H} NMR (151 MHz, $CDCl_3$) δ 161.6, 160.0, 136.7, 134.5, 133.9, 130.5 (d, *J* = 9.2 Hz), 127.6 (d, *J* = 5.3 Hz), 126.3, 124.4, 116.8 (d, *J* = 25.4 Hz), 114.3, 111.1 (d, *J* = 20.5 Hz).

^{19}F-NMR (376 MHz, $CDCl_3$) δ -114.6.

2-Bromo-2'-vinyl-1,1'-binaphthalene (28)

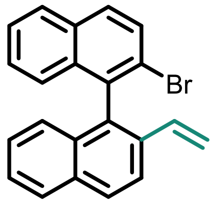

The title compound was prepared according to general procedure C from commercially available starting material with a reaction time of 20 h and 36 h, following Workup Procedure 1. ^{1}H-NMR yield (based on the integration of peaks at 6.09 and 5.83 ppm): for 20 h: 14 %, incomplete conversion; for 36 h: 20 %, incomplete conversion.

5-Vinyl-2-fluoropyridine (29)[11] [CAS 1133879-66-9]

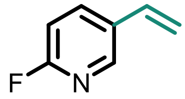

The title compound was prepared according to general procedure B from commercially available starting material with a reaction time of 20 h. ^{1}H-NMR yield (based on the integration of peaks at 6.09 and 5.39 ppm): for 20 h: 33 %, incomplete conversion; for 36 h: 12 %, incomplete conversion.

1-Methyl-5-vinyl-1*H*-benzo[*d*]imidazole (30)

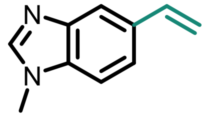

The title compound was prepared according to general procedure D from commercially available starting material, with a reaction time of 18 h following Workup Procedure 1. ^{1}H-NMR yield (based on the integration of peaks at 6.09 and 5.76 ppm): 30 %.

Unsuccessful Substrates

5-Bromo-1*H*-indazole [CAS: 53857-57-1]

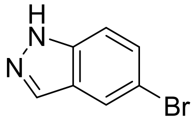

Following the general procedure C, the title compound was subjected to the reaction conditions with a reaction time of 18 h. After Workup Procedure 1, no product formed according to NMR analysis.

Following the general procedure D, the title compound was subjected to the reaction conditions with a reaction time of 18 h. After Workup Procedure 1, no product formed according to NMR analysis.

6-Bromo-1*H*-indazole [CAS: 79762-54-2]

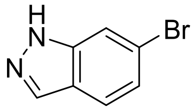

Following the general procedure C, the title compound was subjected to the reaction conditions with a reaction time of 18 h. After Workup Procedure 1, no product formed according to NMR analysis.

Following the general procedure D, the title compound was subjected to the reaction conditions with a reaction time of 18 h. After Workup Procedure 1, no product formed according to NMR analysis.

5-Bromo-1-methyl-1*H*-indazole [CAS: 465529-57-1]

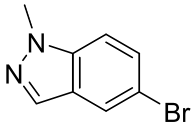

Following the general procedure C, the title compound was subjected to the reaction conditions with a reaction time of 20 h. After Workup Procedure 1, no product formed according to NMR analysis.

5-Bromo-1-methyl-1*H*-benzo[*d*]imidazole [CAS 53484-15-4]

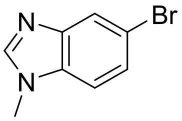

Following the general procedure C, the title compound was subjected to the reaction conditions with a reaction time of 18 h. After Workup Procedure 1, no product formed according to NMR analysis.

tert-Butyl 6-bromo-1H-indazole-1-carboxylate [CAS 147621-26-9]

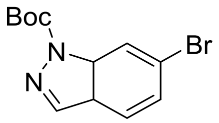

Following the general procedure C, the title compound was subjected to the reaction conditions with a reaction time of 20 h. After Workup Procedure 1, no product formed according to NMR analysis.

5-Bromobenzo[d]oxazole [CAS: 132244-31-6]

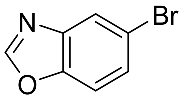

Following the general procedure C, the title compound was subjected to the reaction conditions with a reaction time of 18 h. After Workup Procedure 1, no product formed according to NMR analysis.

5-Bromo-2-methylbenzo[d]oxazole [CAS: 5676-56-2]

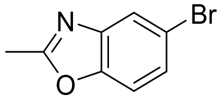

Following the general procedure C, the title compound was subjected to the reaction conditions with a reaction time of 18 h.

Workup procedure 1: No product formation according to NMR analysis.

Workup procedure 2: No product formation according to NMR analysis.

5-Bromofuro[2,3-b]pyridine [CAS 220957-39-1]

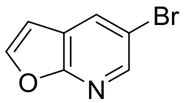

Following the general procedure C, the title compound was subjected to the reaction conditions with a reaction time of 18 h. After Workup Procedure 1, no product formed according to NMR analysis.

2-Bromoquinoline [CAS 2005-43-8]

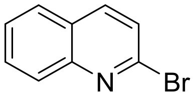

Following the general procedure B, the title compound was subjected to the reaction conditions with a reaction time of 18 h. After Workup Procedure 1, no product formed according to NMR analysis.

3-Bromo-1,8-naphthyridine [CAS 17965-78-5]

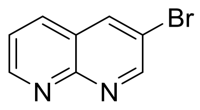

Following the general procedure C, the title compound was subjected to the reaction conditions with a reaction time of 18 h.

Workup procedure 1: No product formation according to NMR analysis.

Workup procedure 2: No product formation according to NMR analysis.

3-Bromo-1,5-naphthyridine [CAS 17965-71-8]

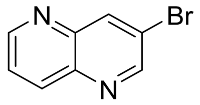

Following the general procedure C, the title compound was subjected to the reaction conditions with a reaction time of 18 h. After Workup Procedure 1, no product formed according to NMR analysis.

6-Bromoquinoxaline [CAS50998-17-9]

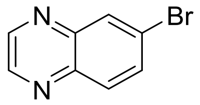

Following the general procedure C, the title compound was subjected to the reaction conditions with a reaction time of 18 h. After Workup Procedure 1, no product formed according to NMR analysis.

2-Bromo-3-methylpyridine [CAS 3430-17-9]

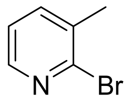

Following the general procedure B, the title compound was subjected to the reaction conditions with a reaction time of 18 h. No product formation according to NMR analysis.

Following the general procedure E, the title compound was subjected to the reaction conditions with a reaction time of 18 h. No product formation according to NMR analysis.

5-Bromo-2-phenylthiazole [CAS 53715-67-6]

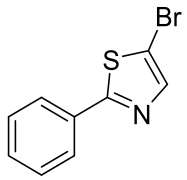

Following the general procedure C, the title compound was subjected to the reaction conditions with a reaction time of 18 h. After Workup Procedure 1, no product formed according to NMR analysis.

Following the general procedure D, the title compound was subjected to the reaction conditions with a reaction time of 18 h. After Workup Procedure 1, no product formed according to NMR analysis.

6-Bromopyrazolo[1,5-*a*]pyrimidine [CAS 705263-10-1]

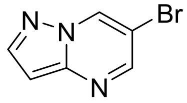

Following the general procedure C, the title compound was subjected to the reaction conditions with a reaction time of 18 h.

Workup procedure 1: No product formation according to NMR analysis.

Workup procedure 2: No product formation according to NMR analysis.

7-Bromo-[1,2,4]triazolo[1,5-*a*]pyridine [CAS 1053655-66-5]

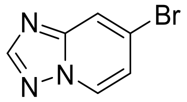

Following the general procedure C, the title compound was subjected to the reaction conditions with a reaction time of 18 h.

Workup procedure 1: No product formation according to NMR analysis.

Workup procedure 2: No product formation according to NMR analysis.

3-Bromobenzonitrile [CAS 6952-59-6]

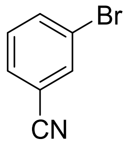

Following the general procedure B, the title compound was subjected to the reaction conditions with a reaction time of 18 h. No product formation according to NMR analysis.

4-Bromobenzonitrile [CAS 623-00-7]-886

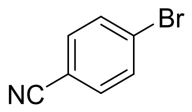

Following the general procedure B, the title compound was subjected to the reaction conditions with a reaction time of 18 h. No product formation according to NMR analysis.

1-Bromo-3-nitrobenzene [CAS 585-79-5]

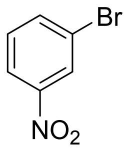

Following the general procedure B, the title compound was subjected to the reaction conditions with a reaction time of 18 h. No product formation according to NMR analysis.

1-Bromo-4-nitrobenzene [CAS 586-78-7]

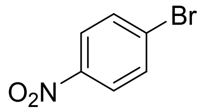

Following the general procedure B, the title compound was subjected to the reaction conditions with a reaction time of 18 h. No product formation according to NMR analysis.

4-Bromobenzamide [CAS698-67-9]

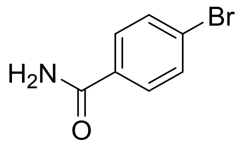

Following the general procedure B, the title compound was subjected to the reaction conditions with a reaction time of 18 h. No product formation according to NMR analysis.

3-Bromobenzaldehyde [CAS 3132-99-8]

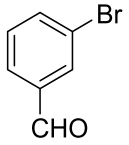

Following the general procedure B, the title compound was subjected to the reaction conditions with a reaction time of 18 h. No product formation according to NMR analysis.

Following the general procedure E, the title compound was subjected to the reaction conditions with a reaction time of 18 h. No product formation according to NMR analysis.

4-Bromopyridin-2(1*H*)-one [36953-37-4]

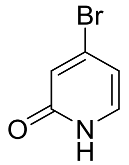

Following the general procedure B, the title compound was subjected to the reaction conditions with a reaction time of 18 h. No product formation according to NMR analysis.

5-Bromo-1-methylpyridin-2-one [CAS: 81971-39-3]

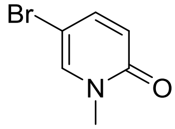

Following the general procedure B, the title compound was subjected to the reaction conditions with a reaction time of 18 h. No product formation according to NMR analysis.

Chlorobenzene [CAS 108-90-7]

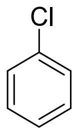

Following the general procedure B, the title compound was subjected to the reaction conditions with a reaction time of 18 h. No product formation according to NMR analysis.

Methyl phenyl sulfone [CAS 3112-85-4]

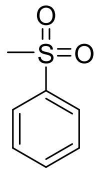

Following the general procedure B, the title compound was subjected to the reaction conditions with a reaction time of 18 h. No product formation according to NMR analysis.

4-Bromophenyl methyl sulfone [CAS 3466-32-8]

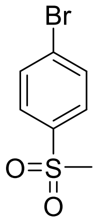

Following the general procedure B, the title compound was subjected to the reaction conditions with a reaction time of 18 h. No product formation according to NMR analysis.

3-Bromo-5-chloropyridine [CAS 5140-72-7]

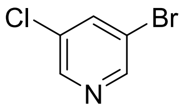

Following the general procedure B, the title compound was subjected to the reaction conditions with a reaction time of 18 h. No product formation according to NMR analysis.

NMR-Spectra of isolated compounds

2-Vinyl-6-methoxynaphthalene (2)

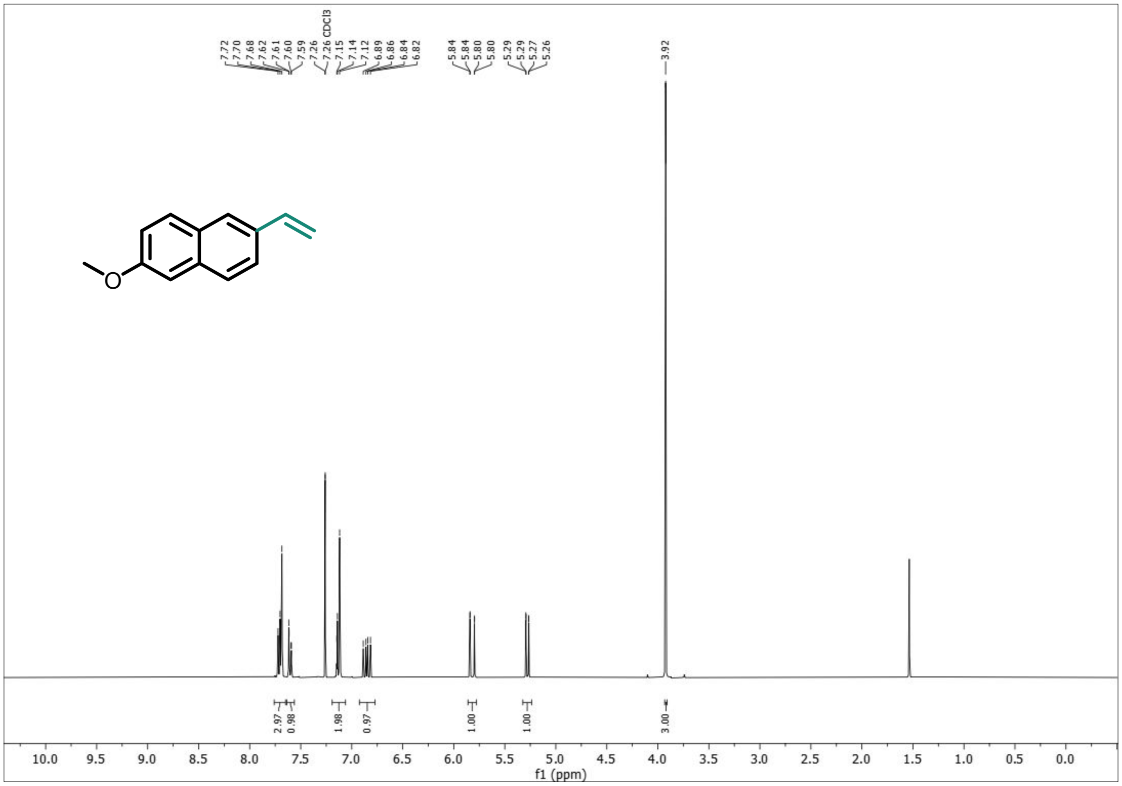

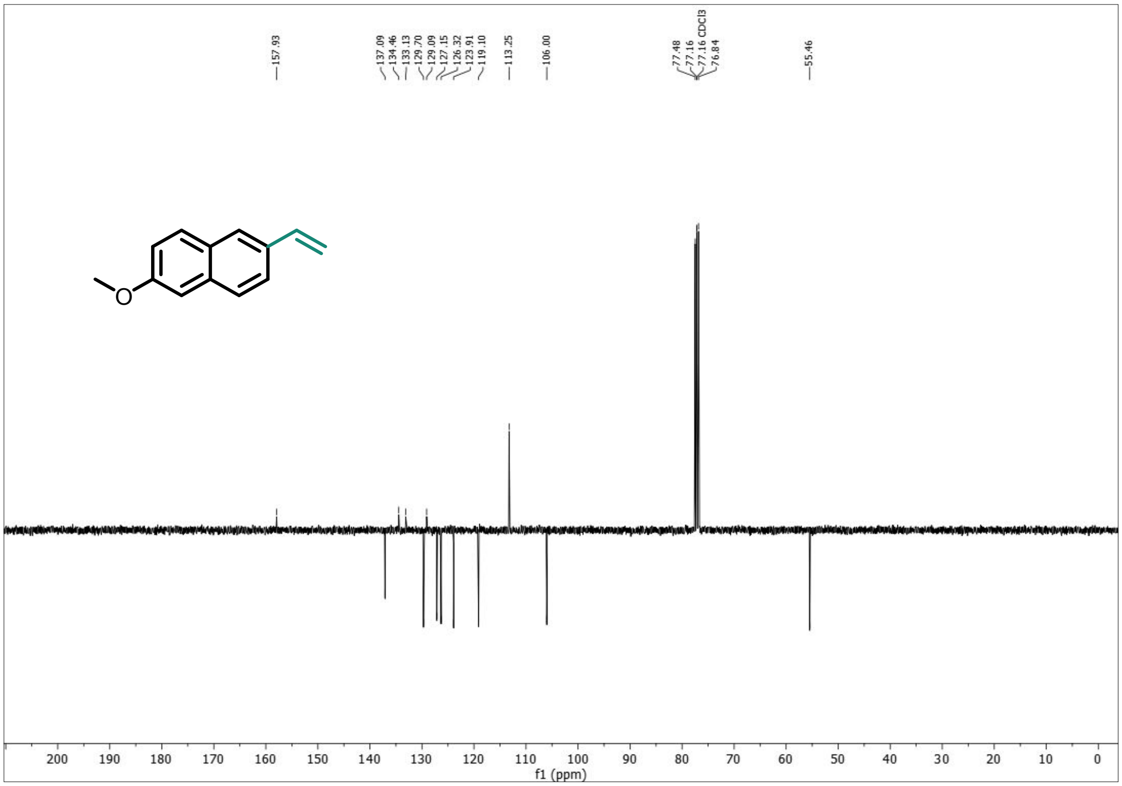

Figure S2: ^{1}H-NMR (400 MHz, $CDCl_3$) and ^{13}C{1H} NMR (101 MHz, $CDCl_3$) of 2-vInyl-6-methoxynapththalene (2).

1-Methyl-6-vinyl-1*H*-indazole (17)

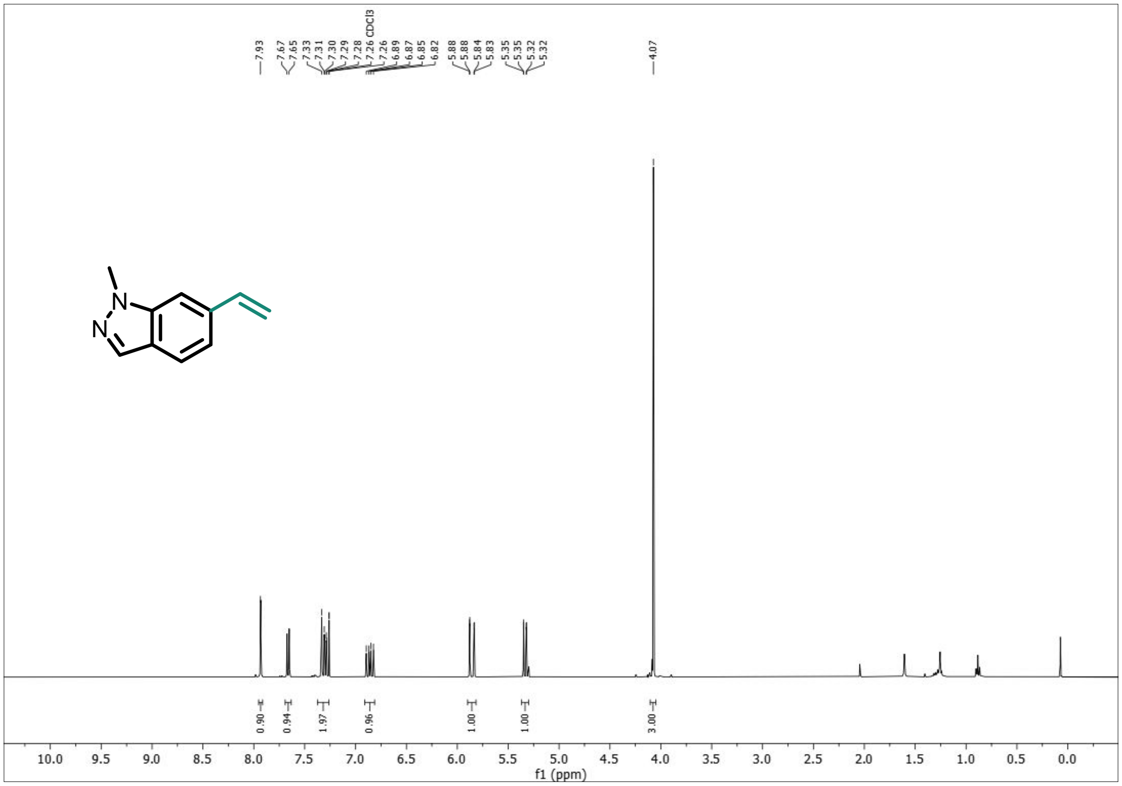

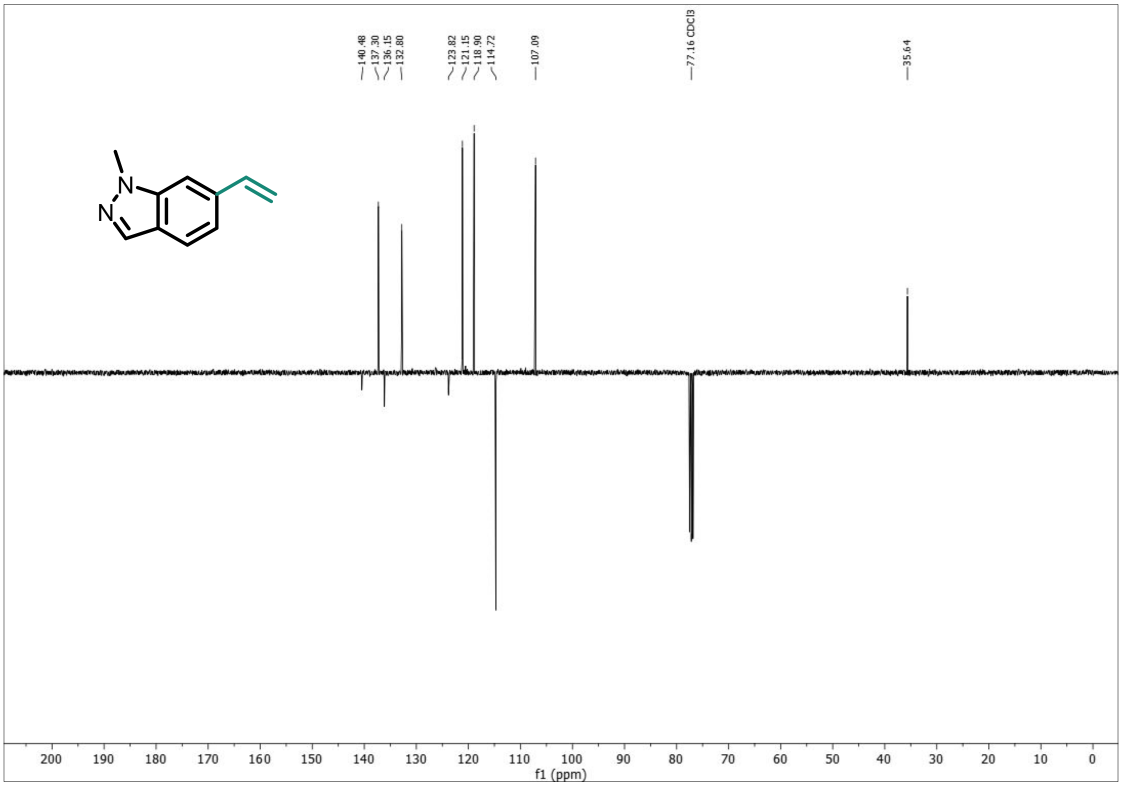

Figure S3: ^{1}H-NMR (400 MHz, $CDCl_3$) and $^{13}C\{1H\}$ NMR (101 MHz, $CDCl_3$) of 1-methyl-6-vinyl-1H-indazole (17).

1-Methoxy-6-vinylisoquinoline (18)

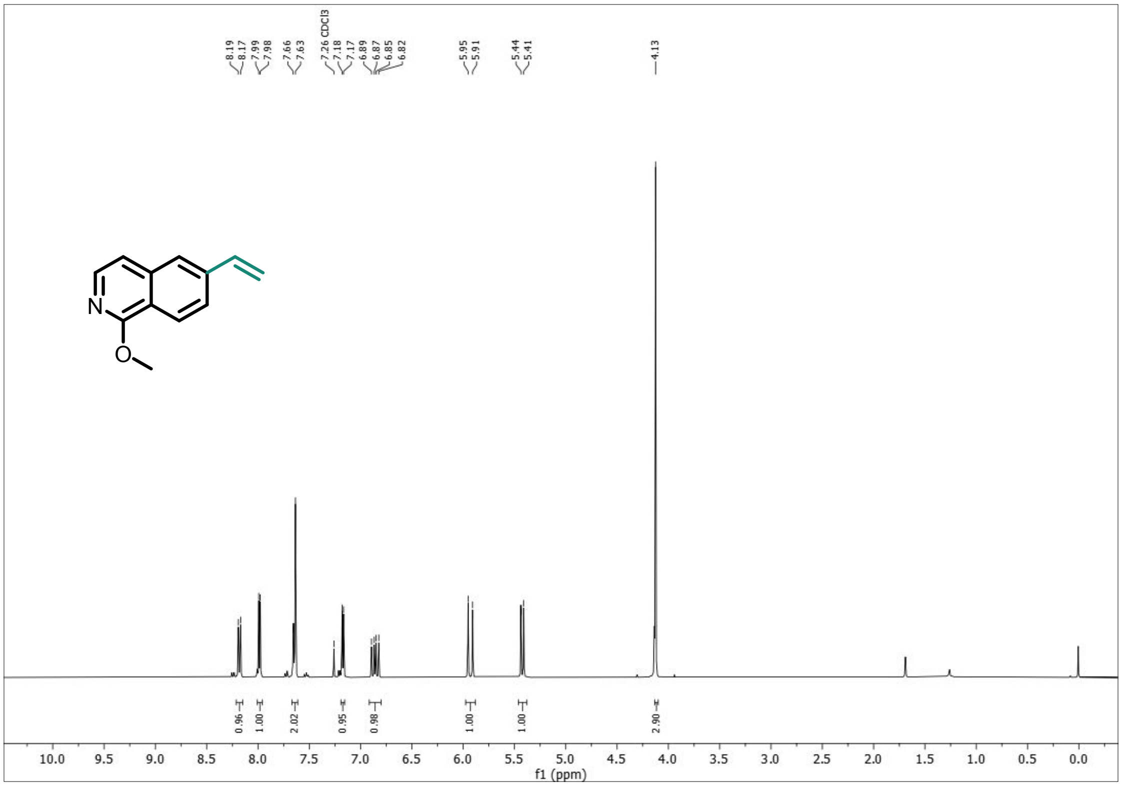

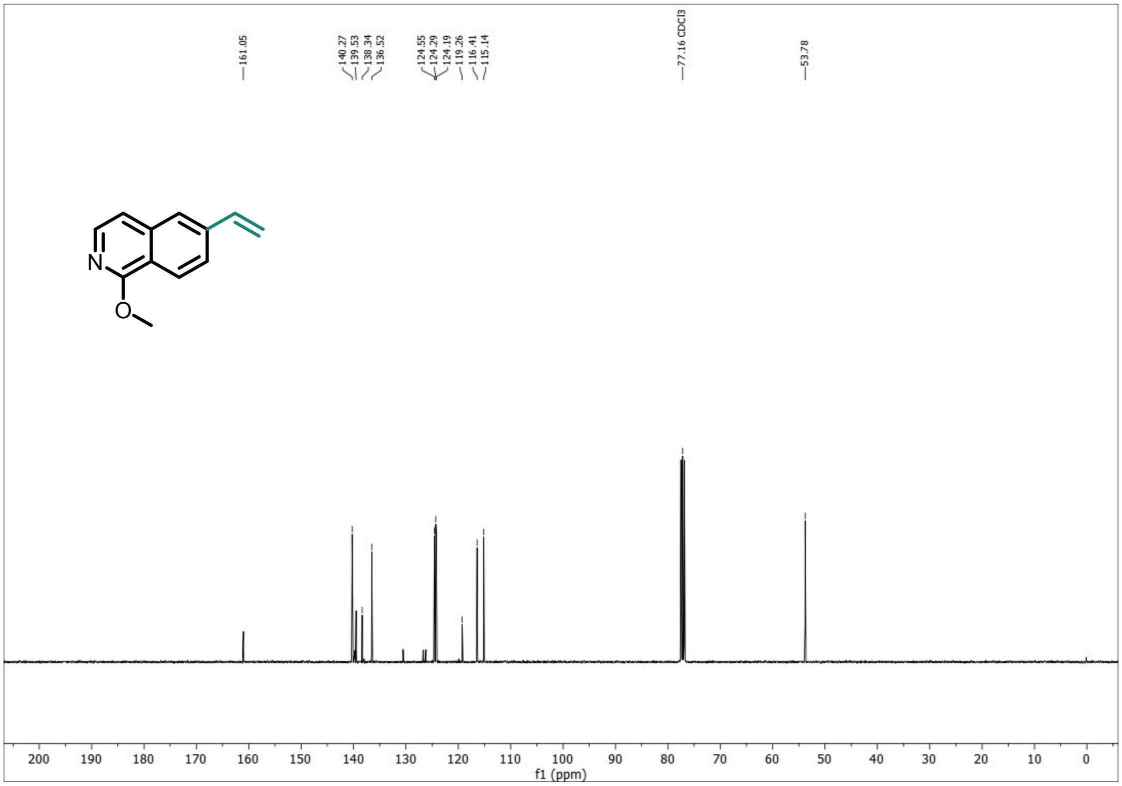

Figure S4: ^{1}H-NMR (400 MHz, $CDCl_3$) and ^{13}C{1H} NMR (101 MHz, $CDCl_3$) of 1-methoxy-6-vinylquinoline (18).

3-Vinylquinoline (19)

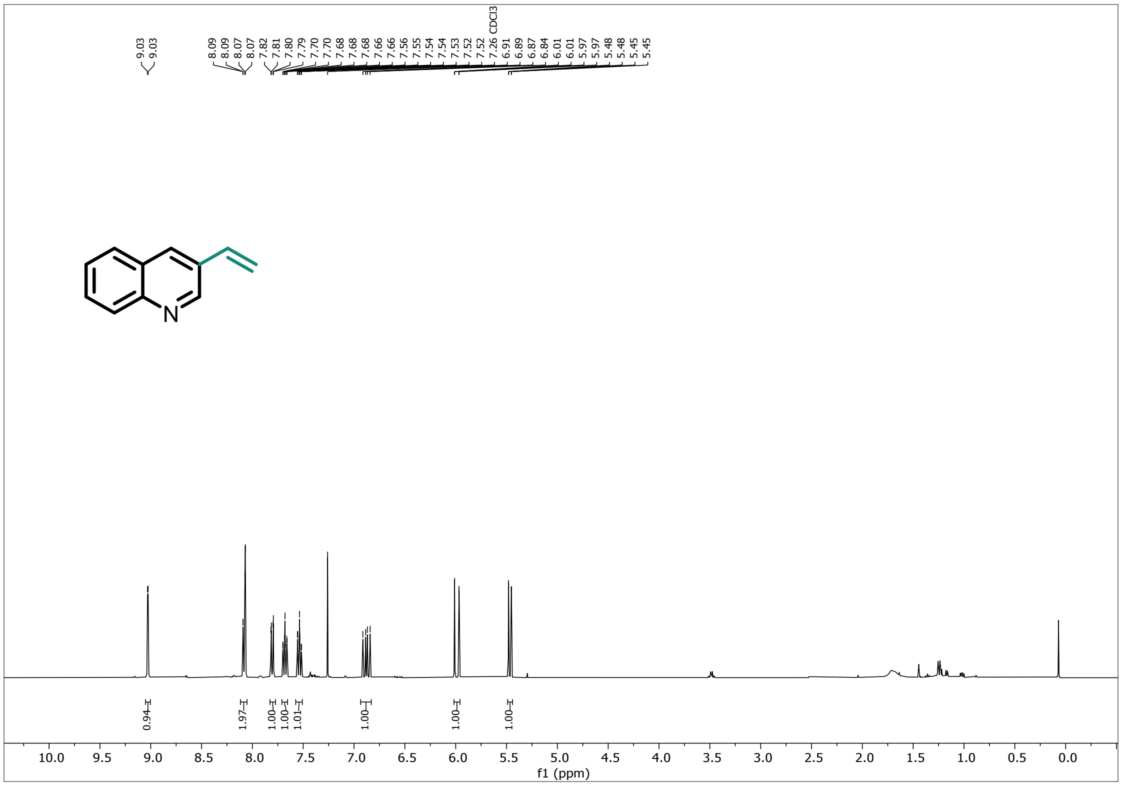

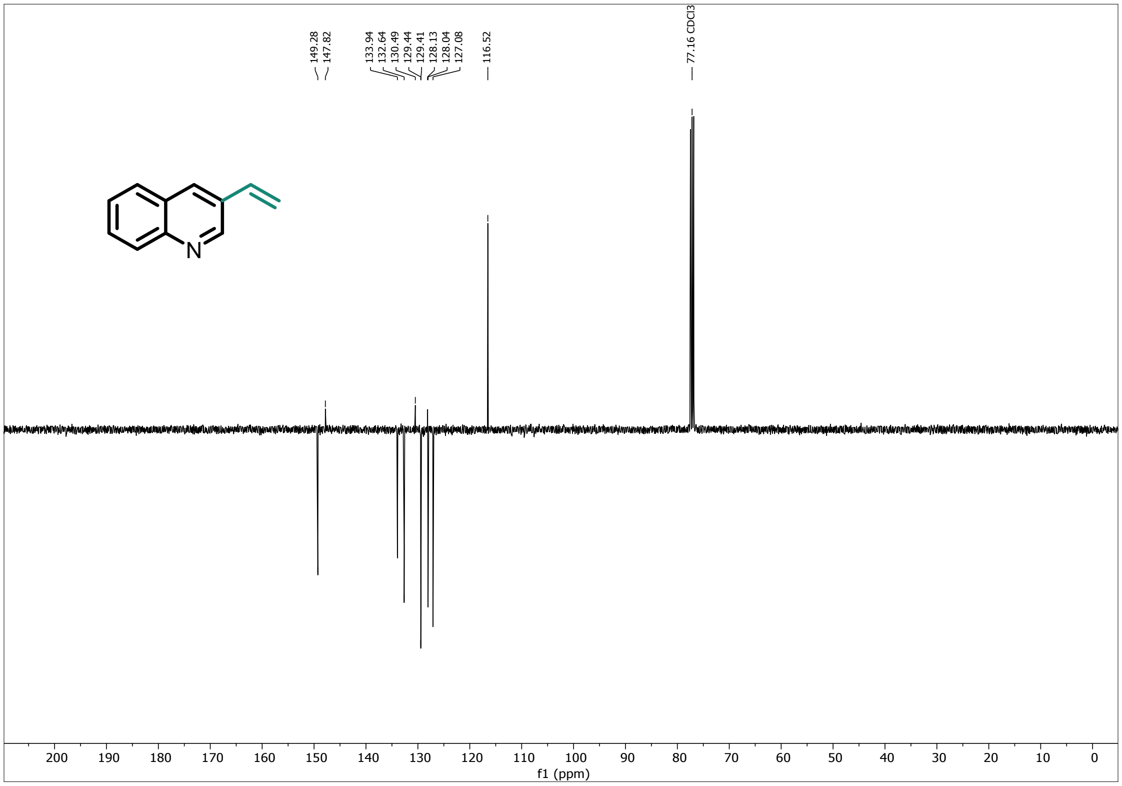

Figure S5: ^{1}H-NMR (400 MHz, $CDCl_3$) and $^{13}C\{1H\}$ NMR (101 MHz, $CDCl_3$) of 3-vinylquinoline (19).

4-Vinylbenzofuran (21)

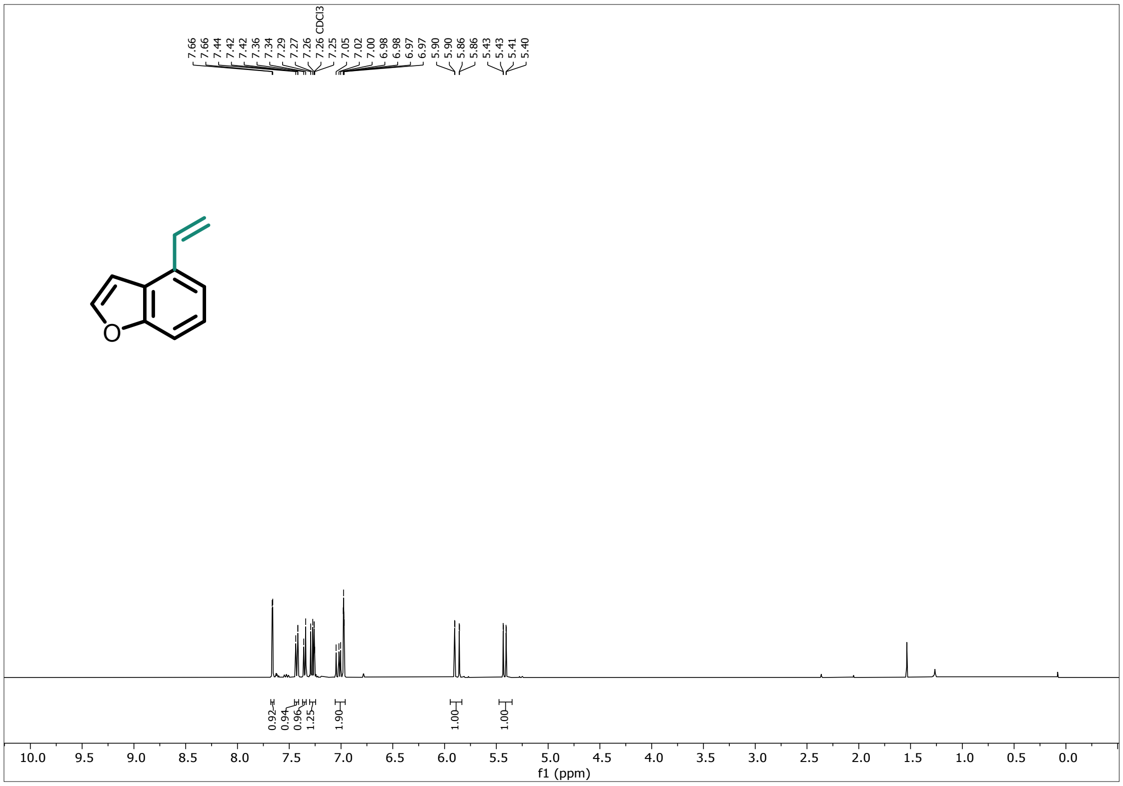

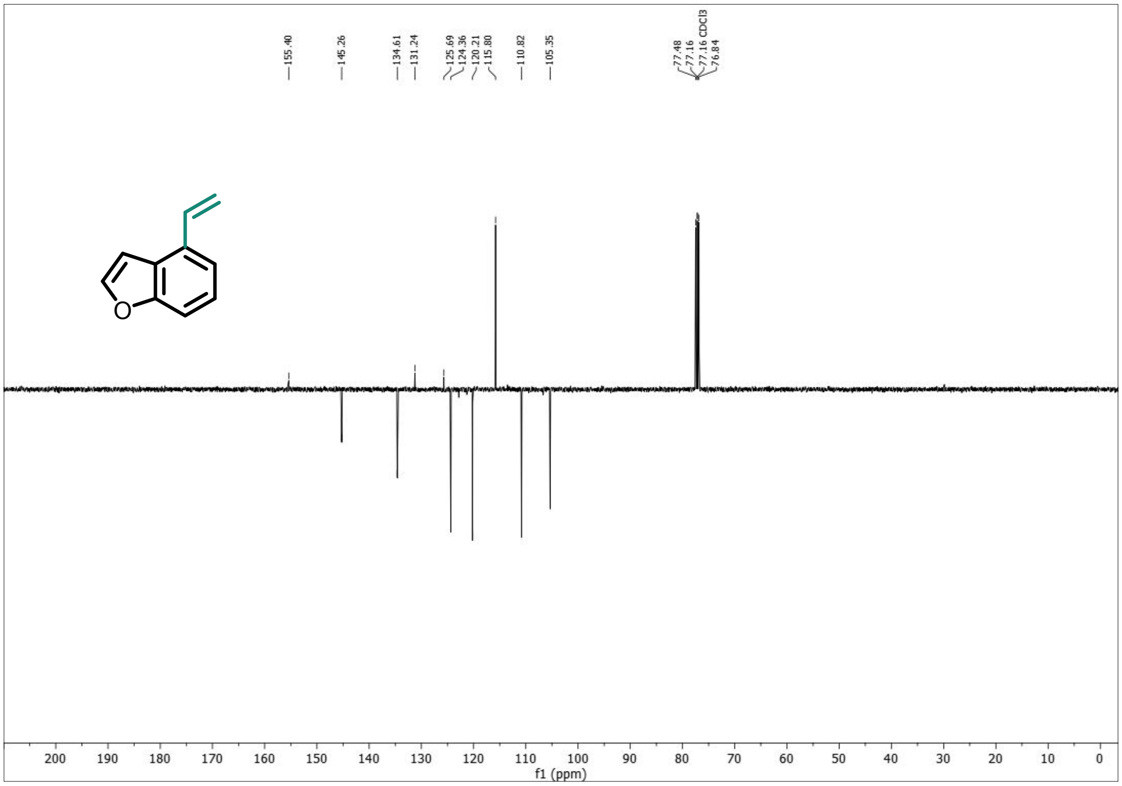

Figure S6: ^{1}H-NMR (400 MHz, $CDCl_3$) and ^{13}C{1H} NMR (101 MHz, $CDCl_3$) of 4-vinylbenzofuran (21).

5-Vinylbenzo[*b*]thiophene (22)

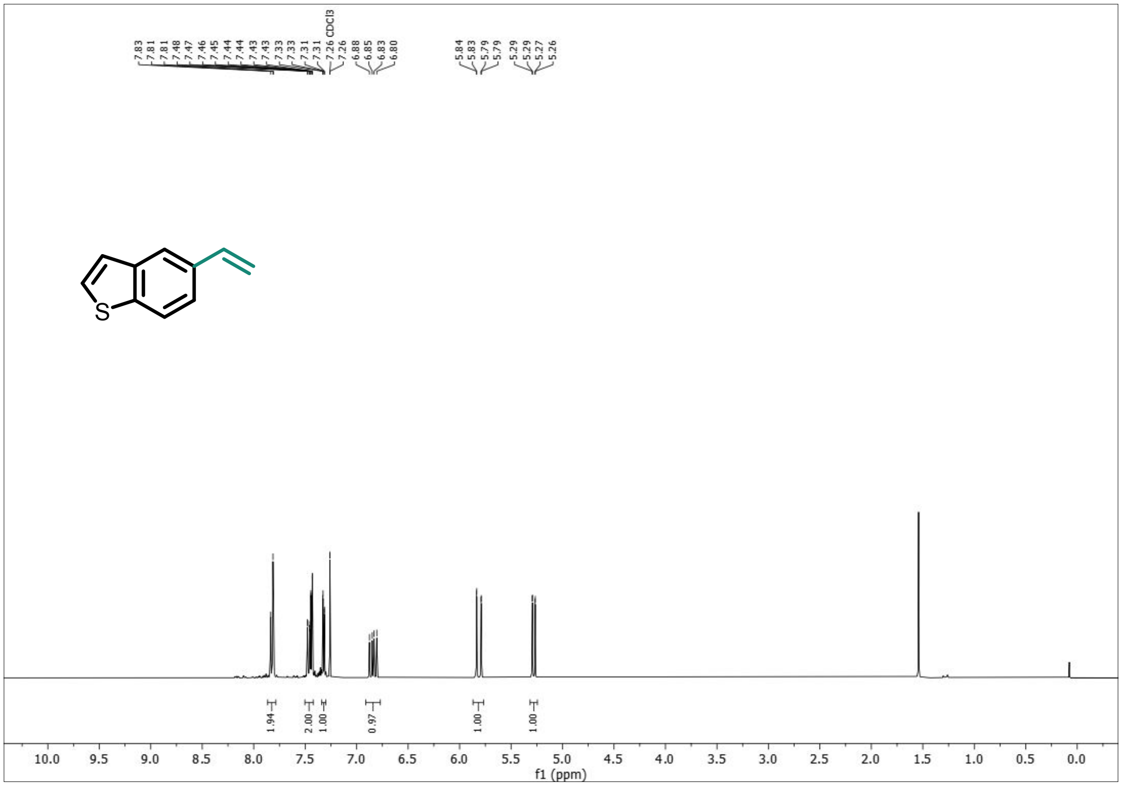

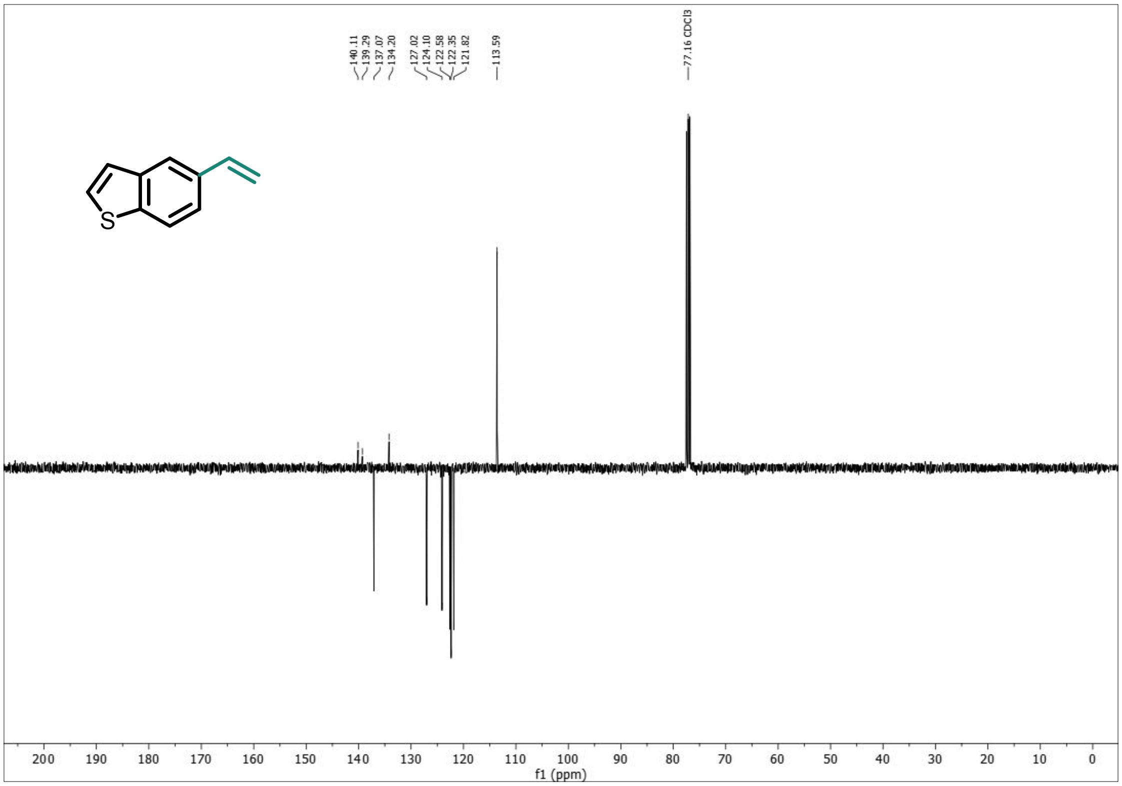

Figure S7: ^{1}H-NMR (400 MHz, $CDCl_3$) and $^{13}C\{1H\}$ NMR (101 MHz, $CDCl_3$) of 5-vinyl-benzo[b]thiophene (22).

2-Vinyl-6-fluoronaphthalene (23)

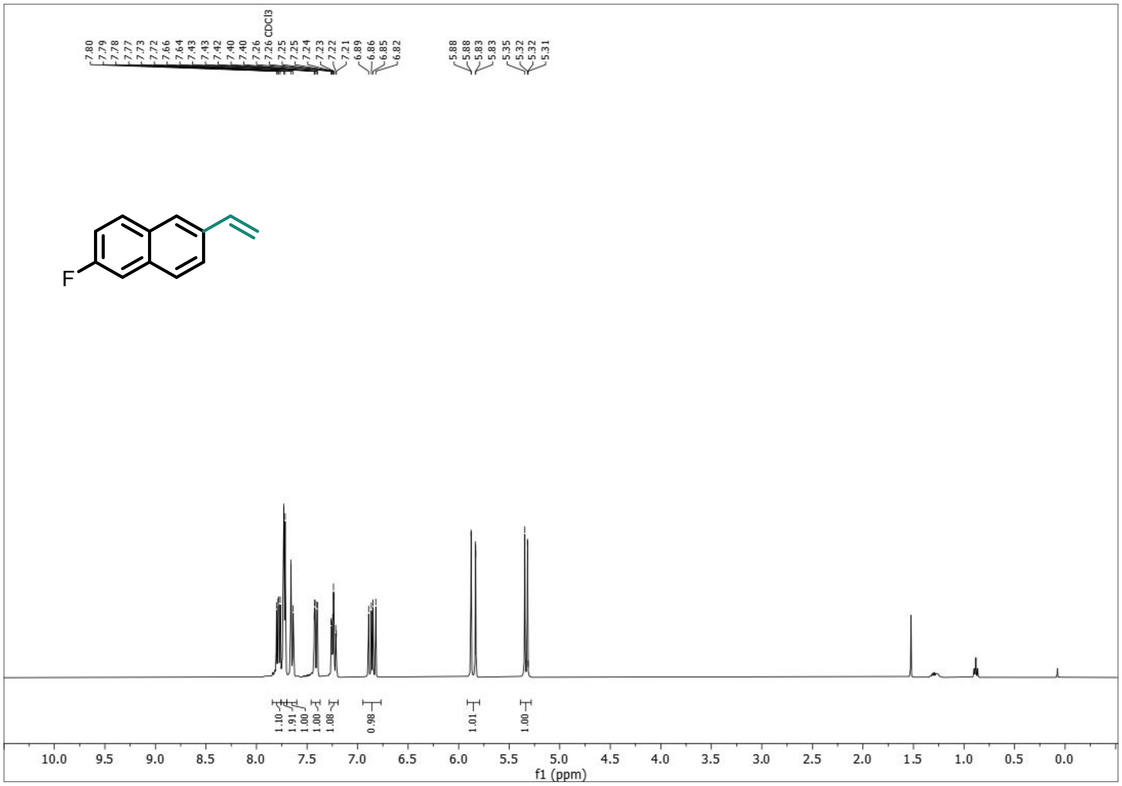

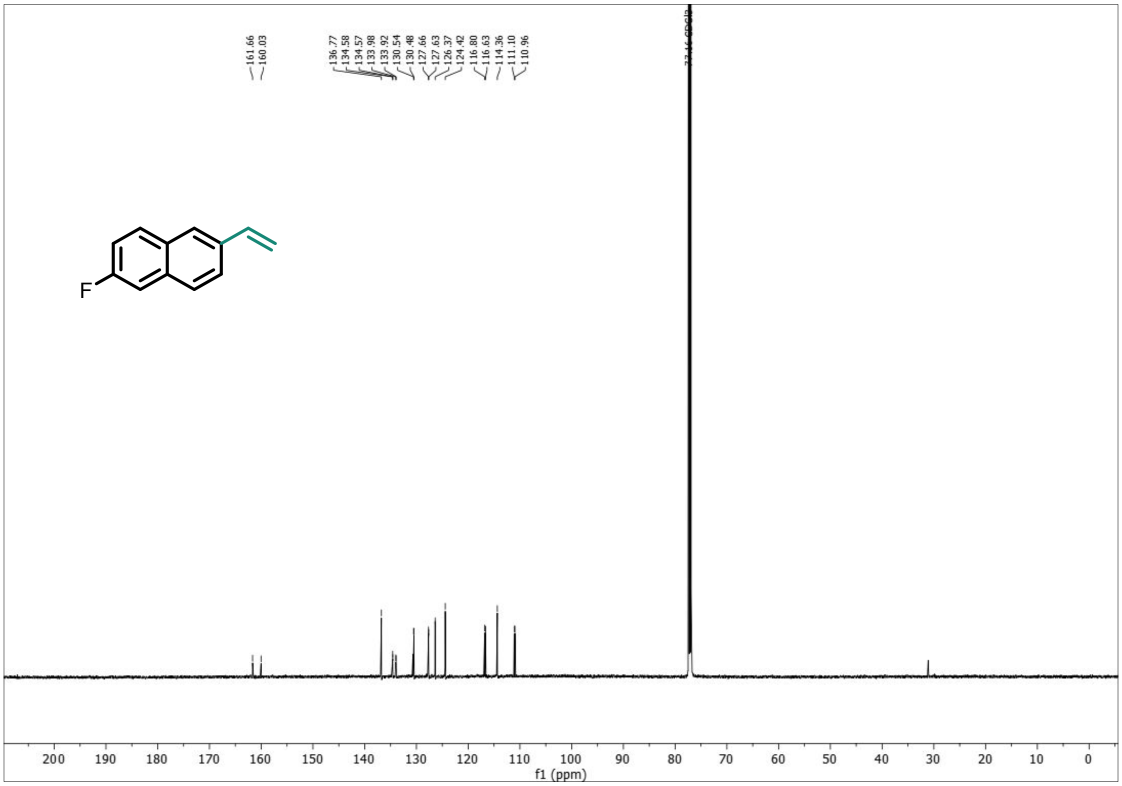

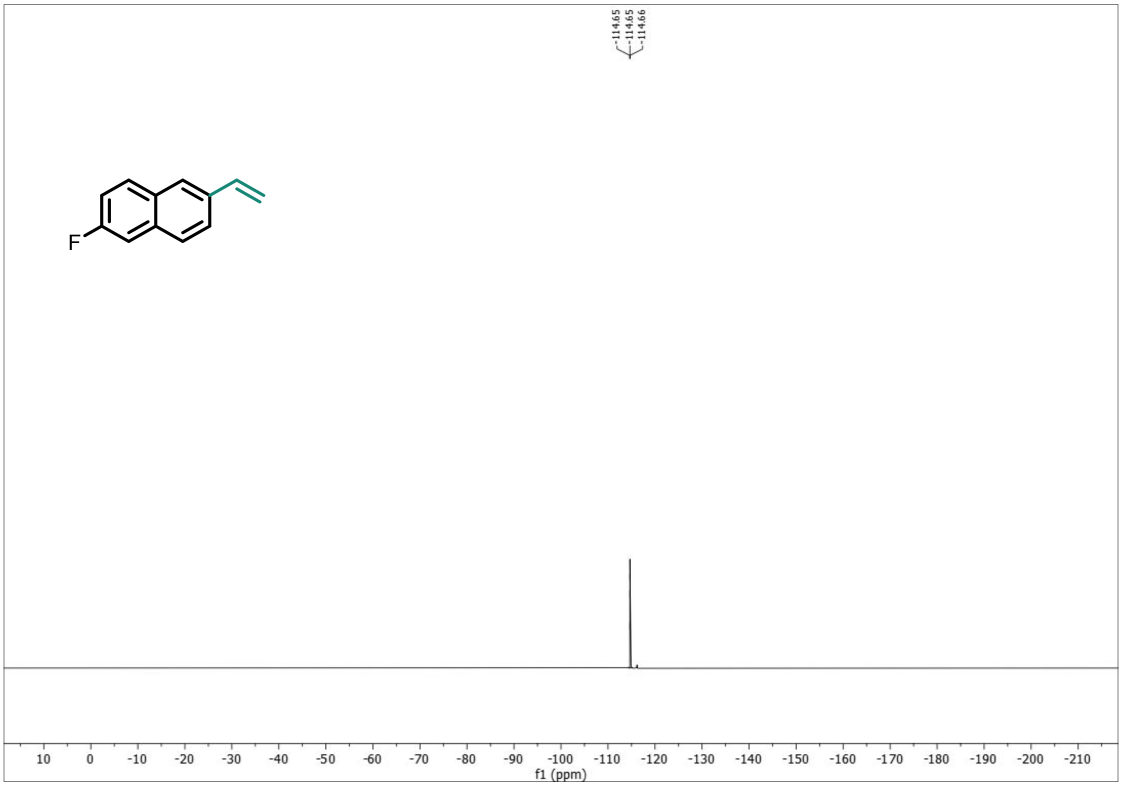

Figure S8: ^{1}H-NMR (400 MHz, $CDCl_3$) $^{13}C\{1H\}$ NMR (101 MHz, $CDCl_3$) and ^{19}F-NMR (376 MHz, $CDCl_3$) of 2-vinyl-6-fluoronapthalene (23).

NMR-Spectra of volatile compounds for quantification

For quantification only the vinylic protons (not overlapping with other peaks of the crude mixture) of the corresponding product and the aromatic protons of the internal standard were integrated and used for the calculation of the yield.

Styrene (3)

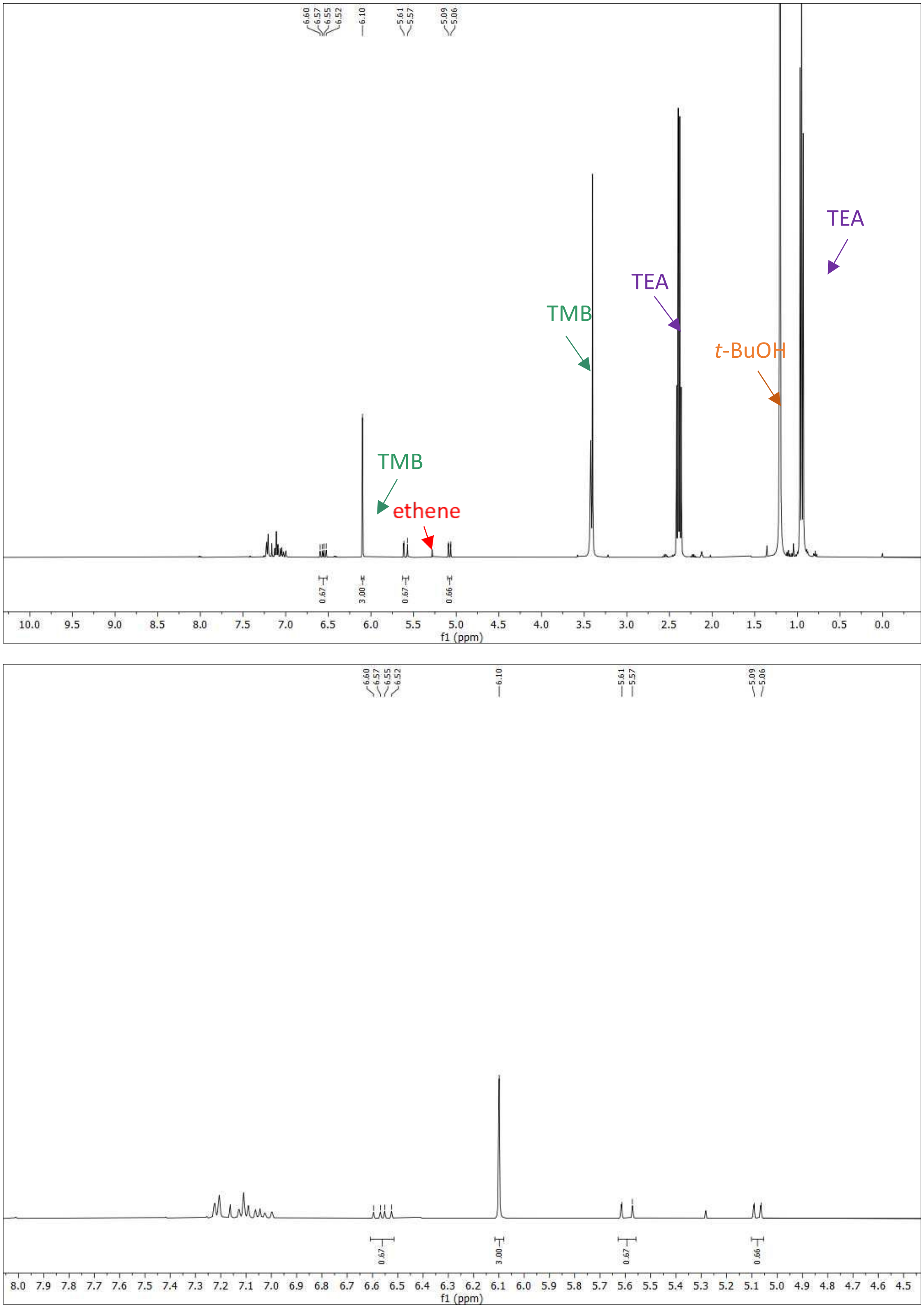

Figure S9: ^{1}H-NMR (400 MHz, C_7D_8) used for quantification of styrene (3) when X=Br.

Styrene (3)

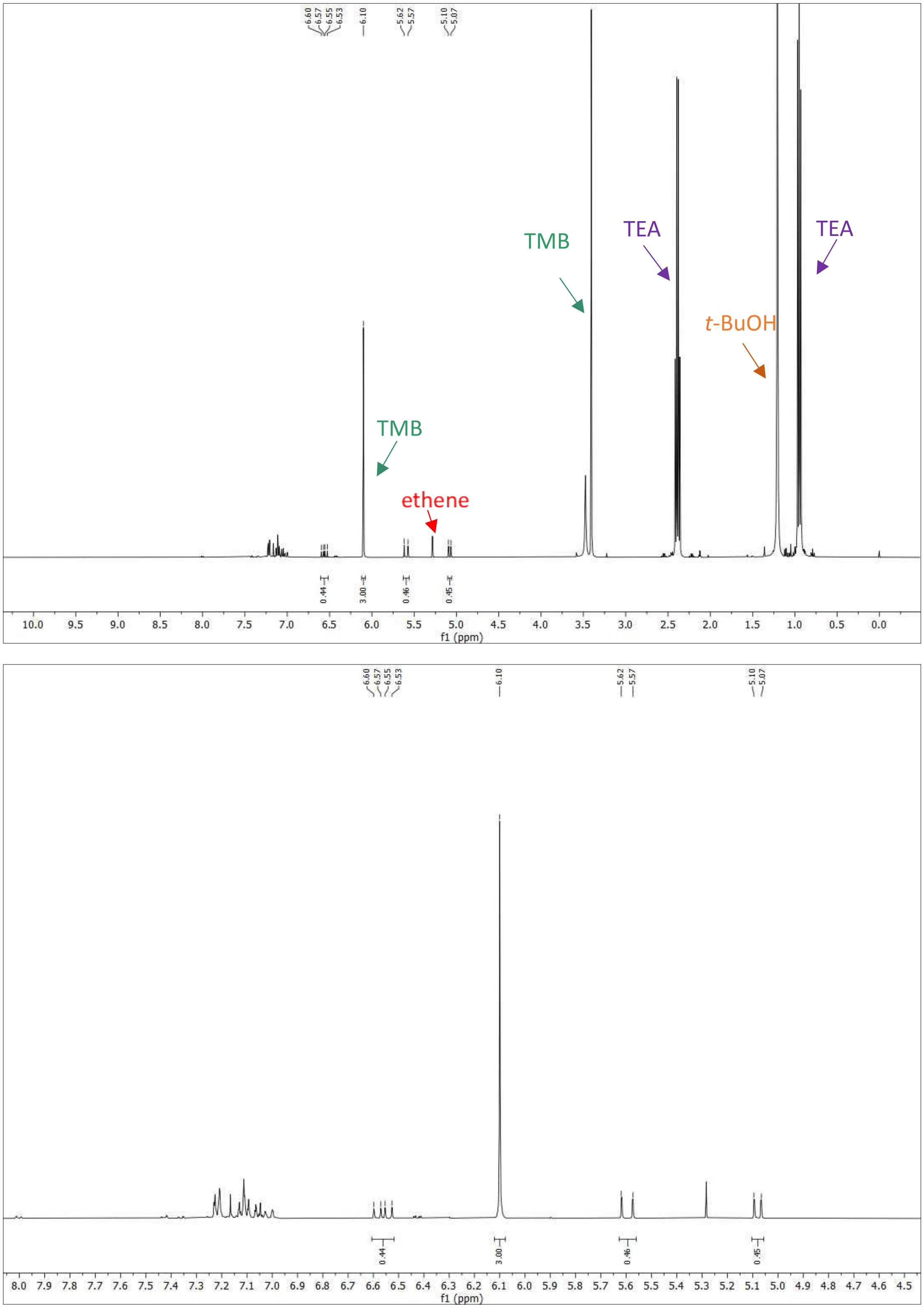

Figure S10: ^{1}H-NMR (400 MHz, C_7D_8) used for quantification of styrene (3) when X=I.

1-(Trifluoromethyl)-3-vinylbenzene (4)

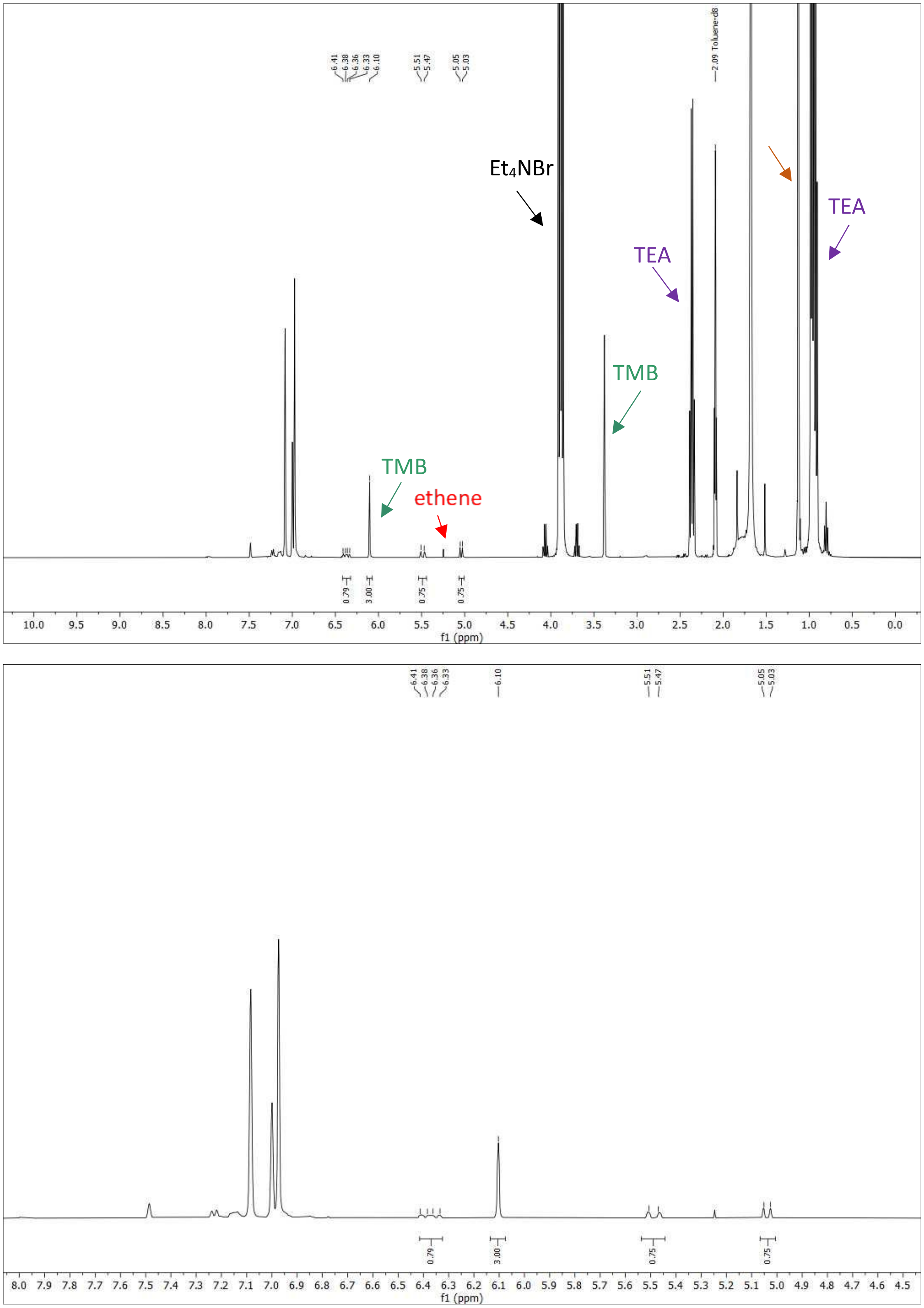

Figure S11: ^{1}H-NMR (400 MHz, C_7D_8) used for quantification of 1-(trifluoromethyl)-3-vinylbenzene (4).

1-(Trifluoromethyl)-4-vinylbenzene (5)

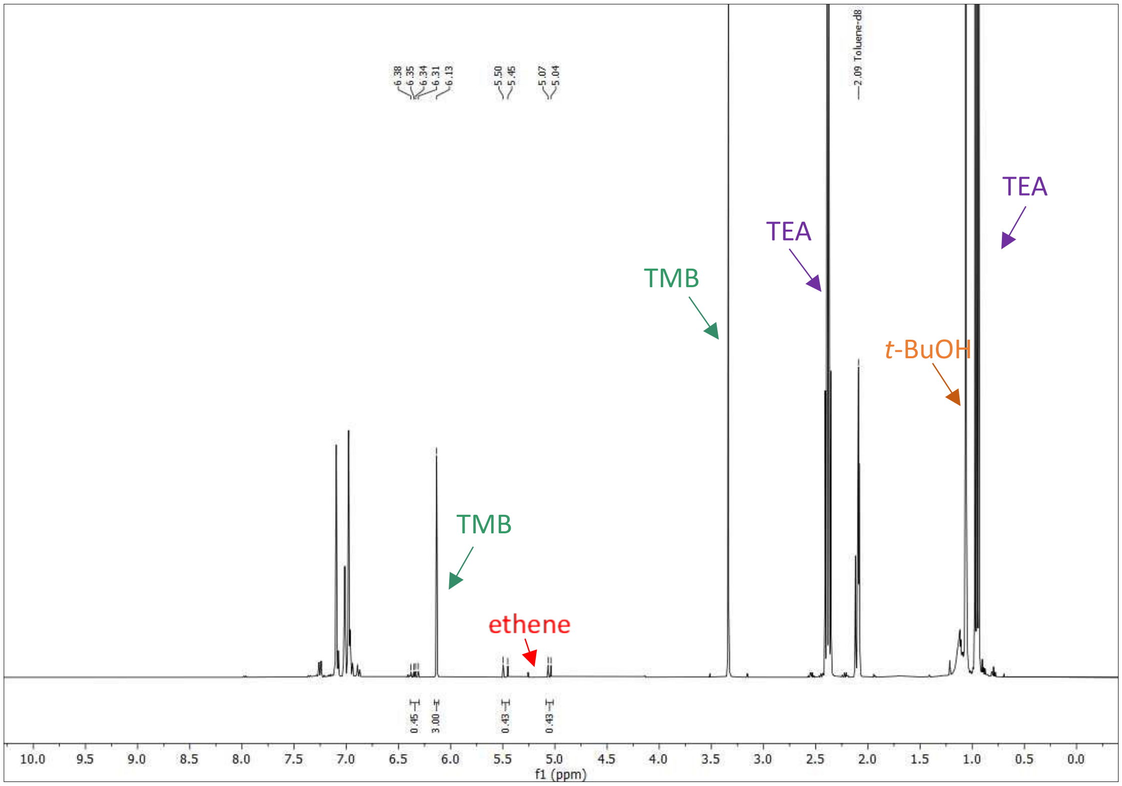

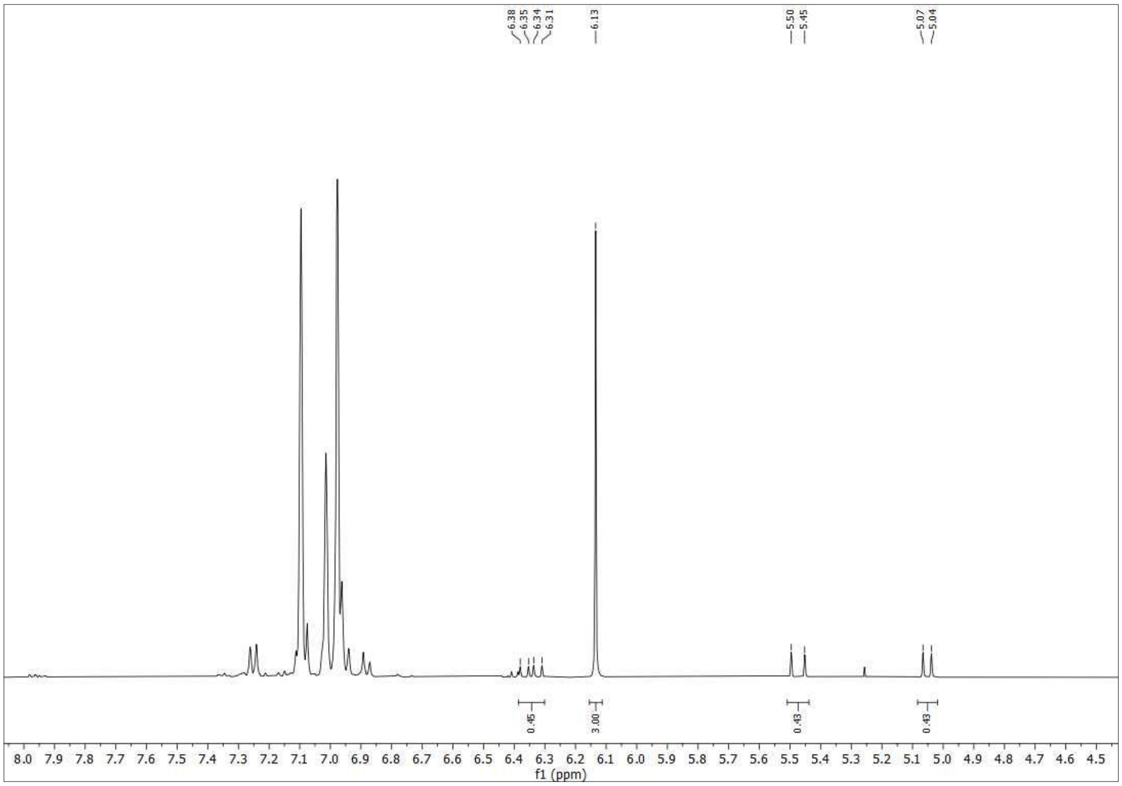

Figure S12: ^{1}H-NMR (400 MHz, C_7D_8) used for quantification of 1-(trifluoromethyl)-4-vinylbenzene (5).

1-(Trifluoromethyl)-2-vinylbenzene (6)

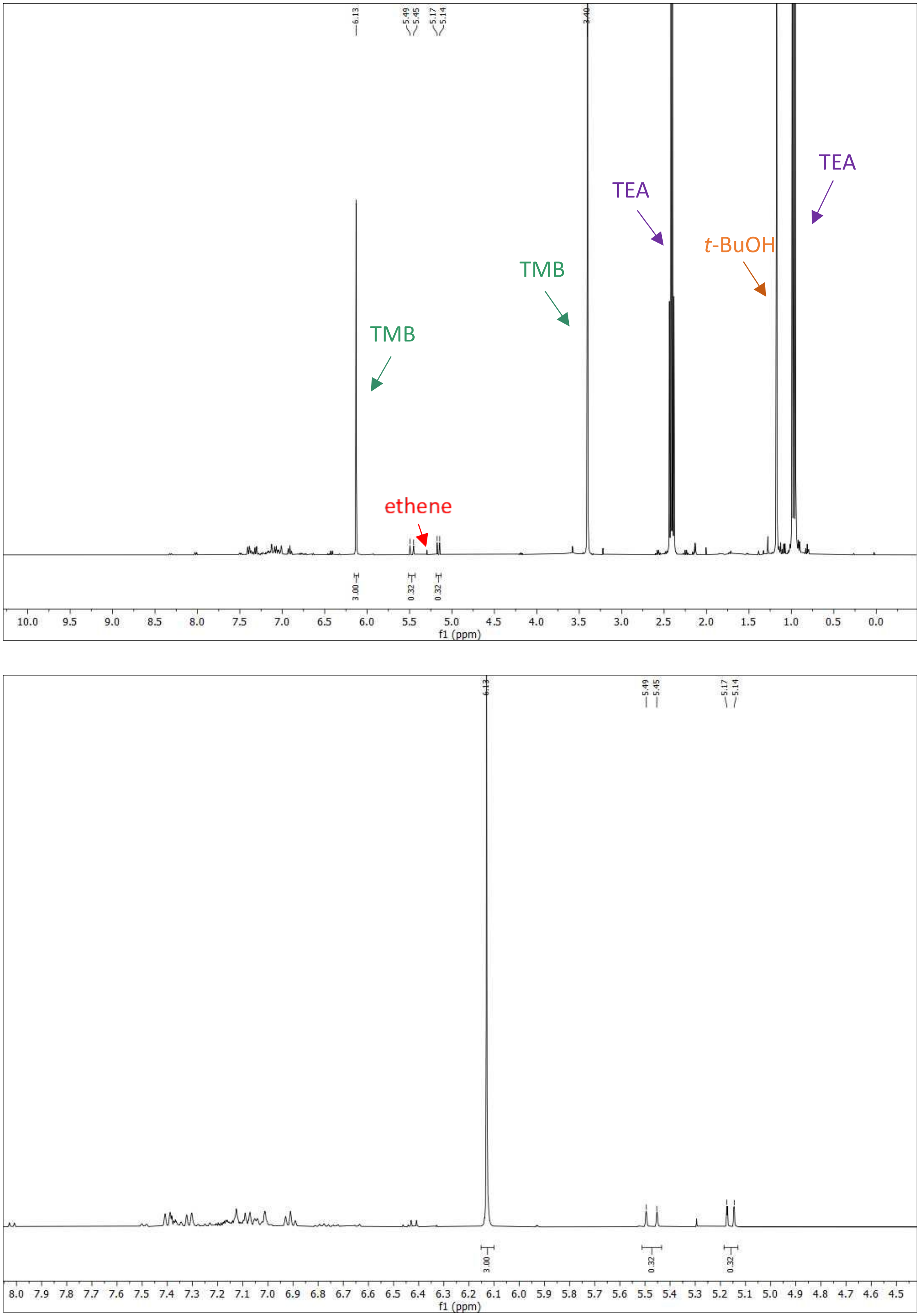

Figure S13: ^{1}H-NMR (400 MHz, C_7D_8) used for quantification of 1-(trifluoromethyl)-2-vinylbenzene (6).

4-vinylbenzoic acid (7)

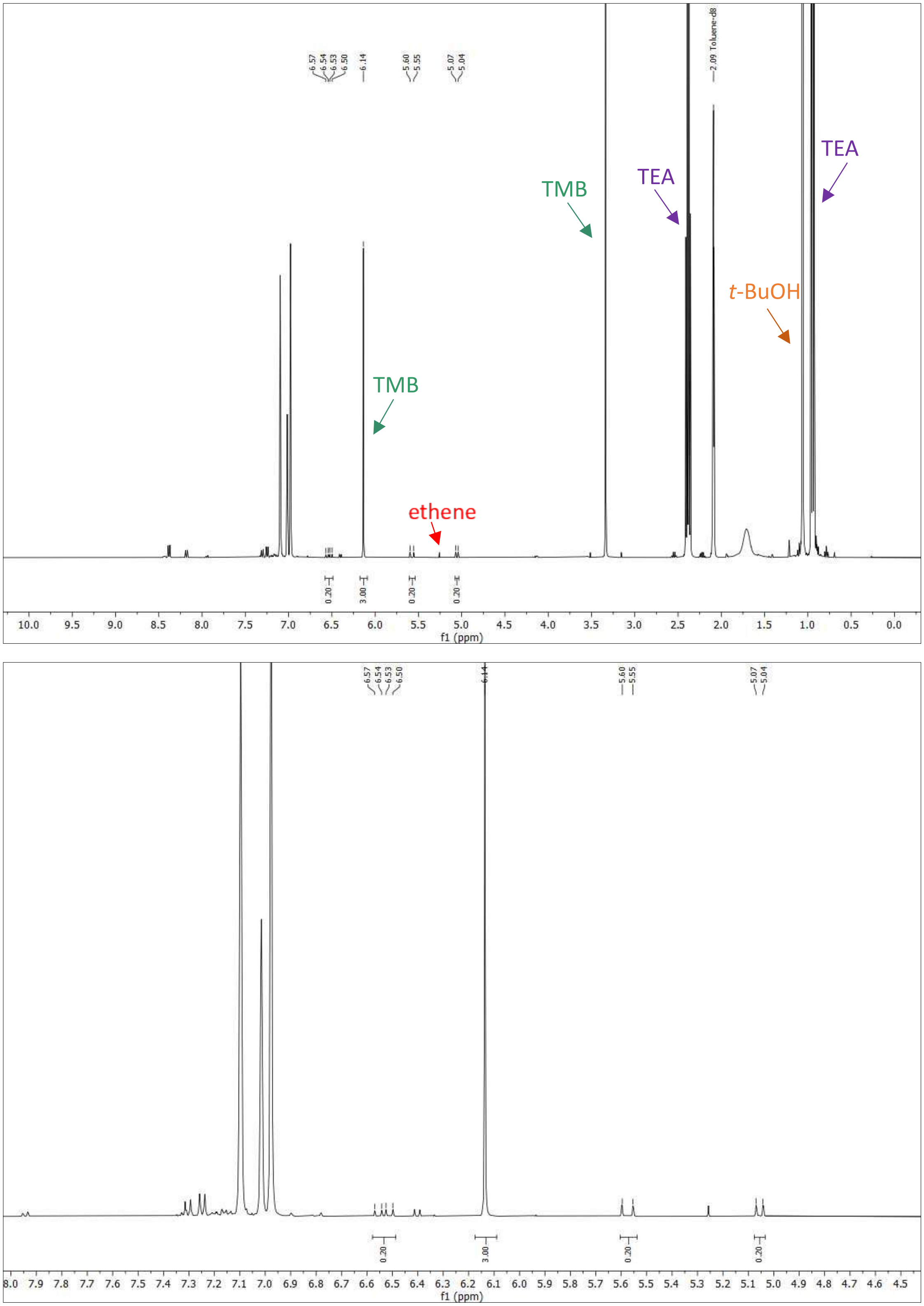

Figure S14: ¹H-NMR (400 MHz, C_7D_8) used for quantification of 4-vinylbenzoic acid (7).

1-Methoxy-3-vinylbenzene (8)

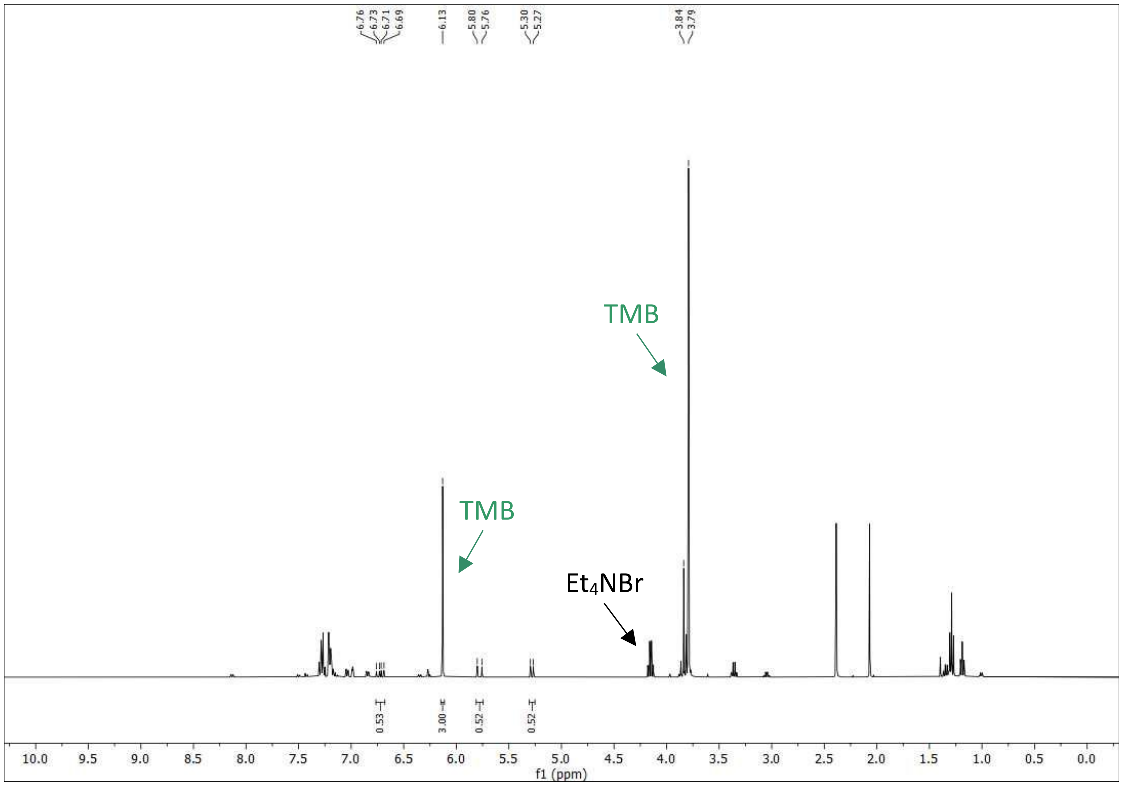

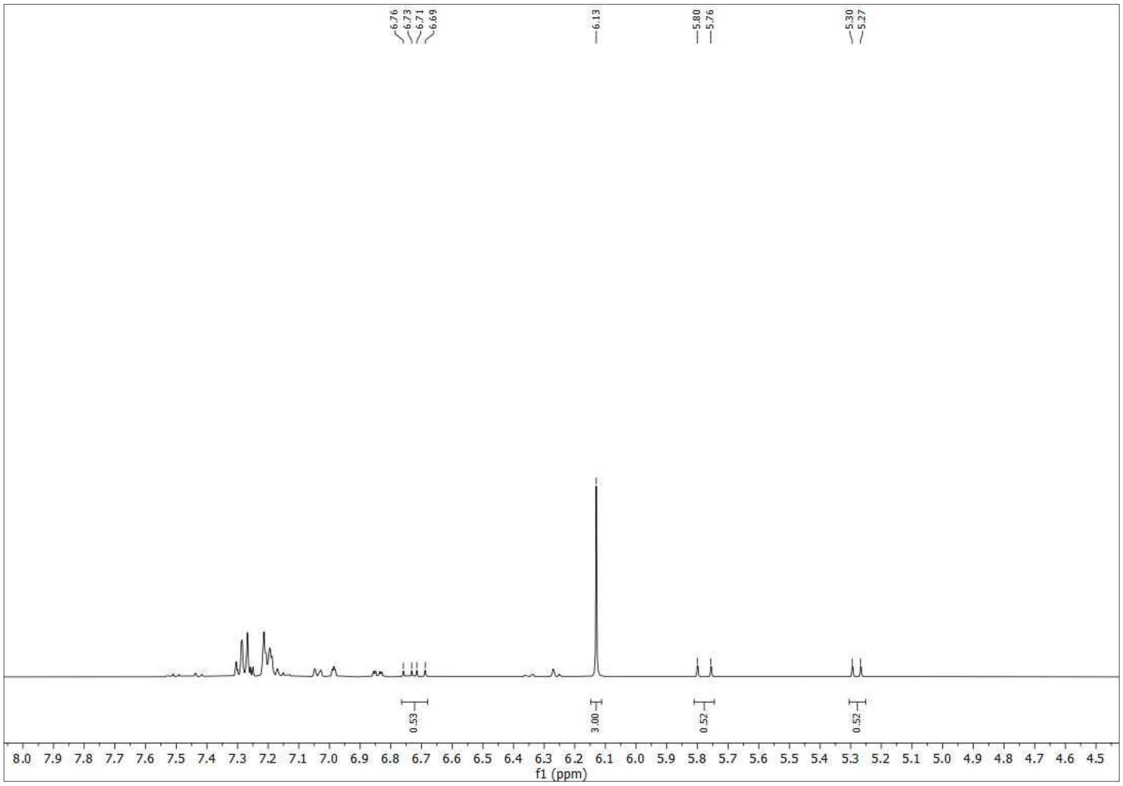

Figure S15: ^{1}H-NMR (400 MHz, C_7D_8) used for quantification of 1-methoxy-3-vinylbenzene (8).

1-Methoxy-4-vinylbenzene (9)

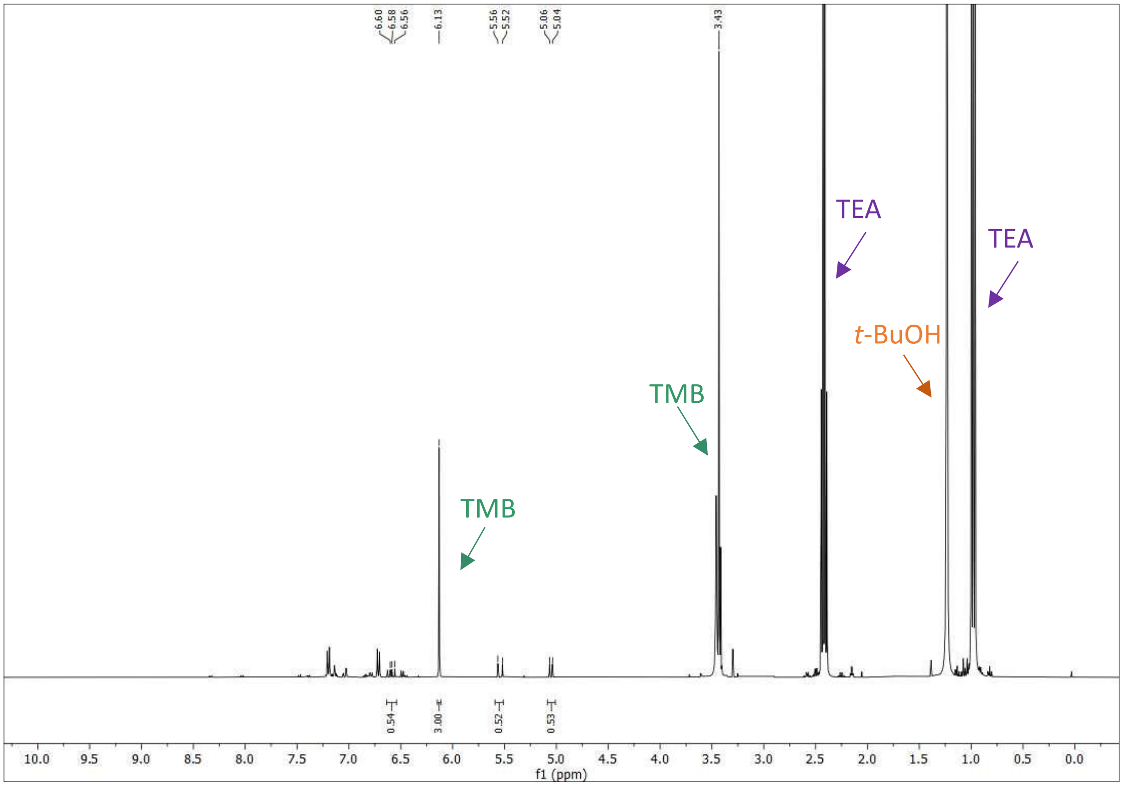

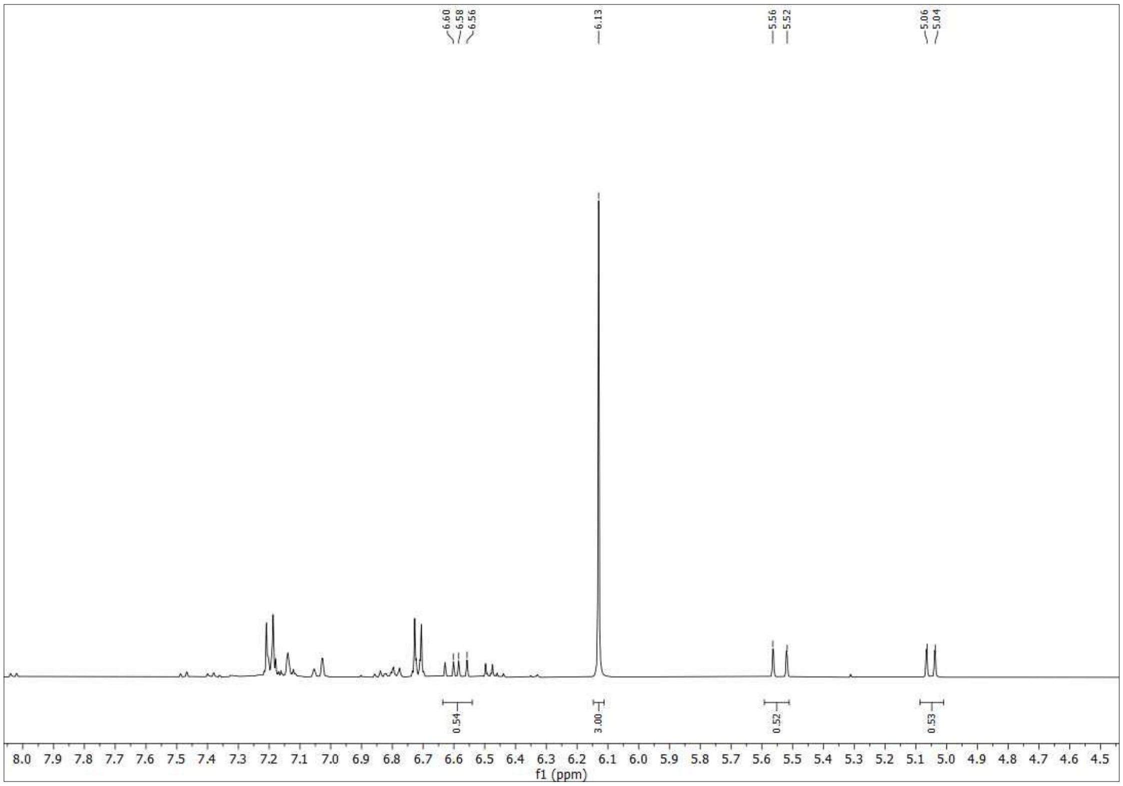

Figure S16: ^{1}H-NMR (400 MHz, C_7D_8) used for quantification of 1-methoxy-4-vinylbenzene (9).

1-Methoxy-2-vinylbenzene (10)

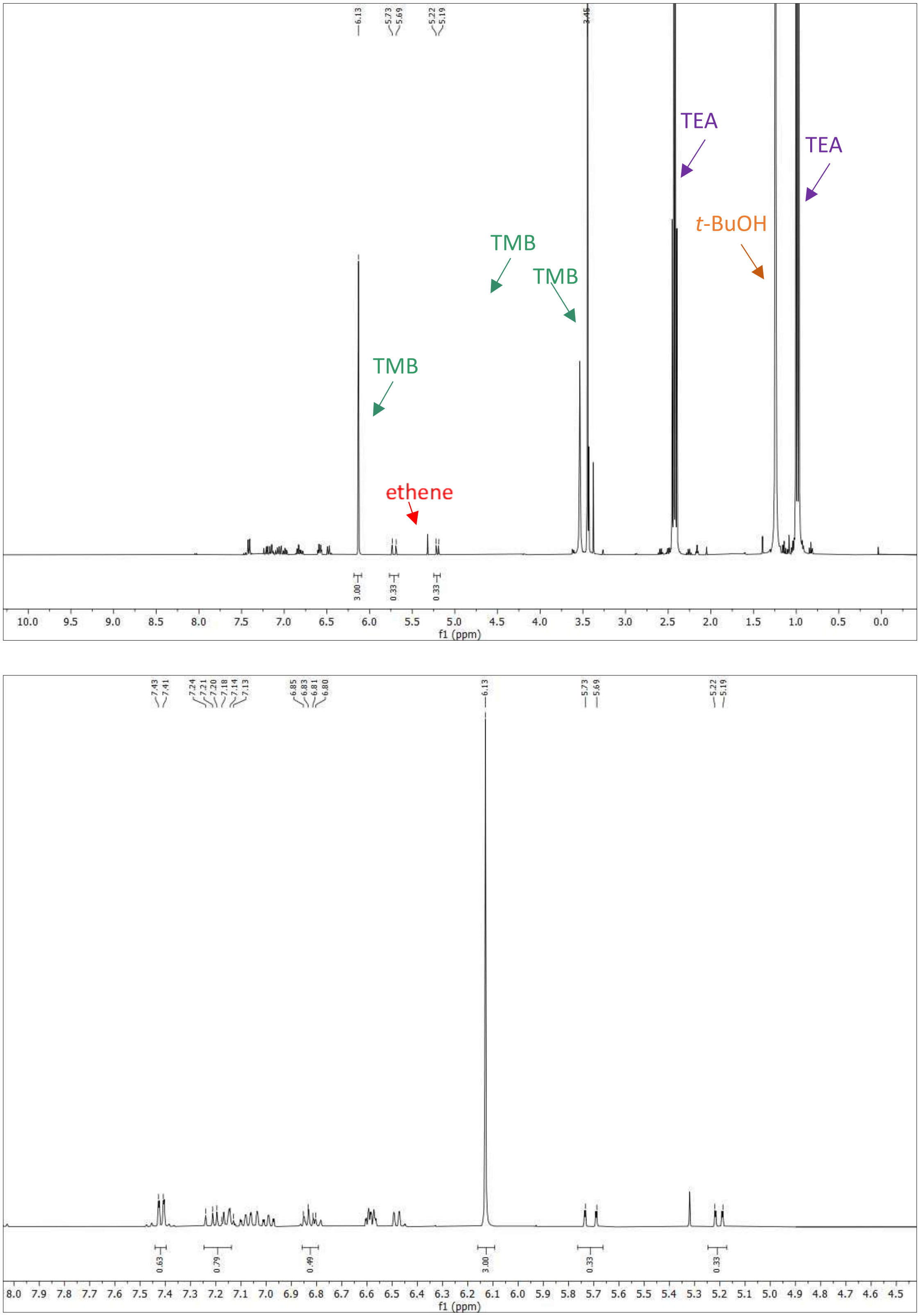

Figure S17: ^{1}H-NMR (400 MHz, C_7D_8) used for quantification of 1-methoxy-2-vinylbenzene (10).

N,N-Dimethyl-4-vinylaniline (11)

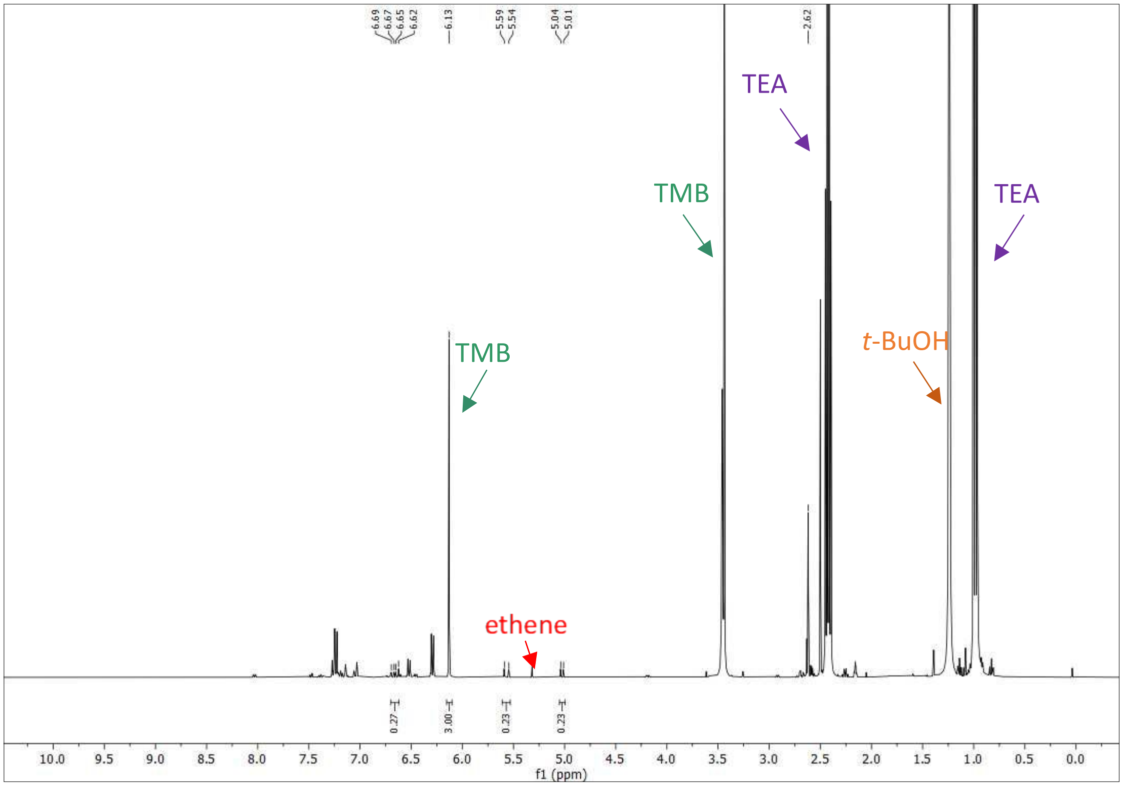

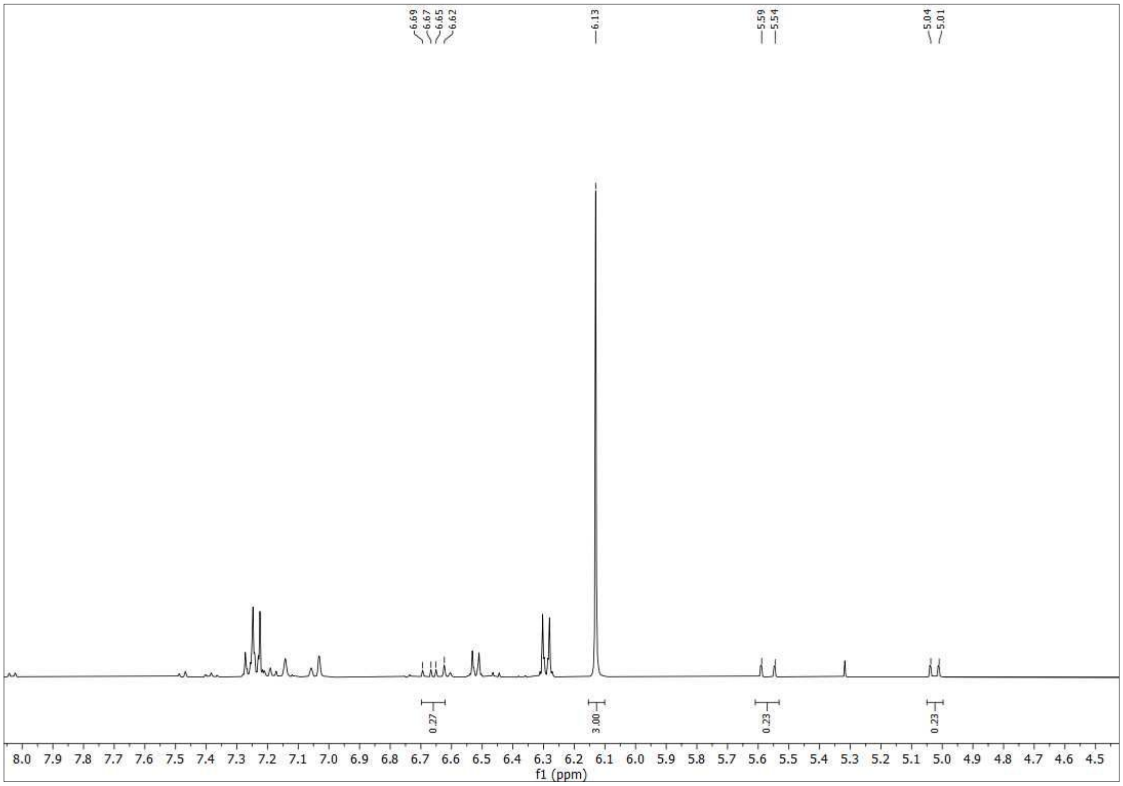

Figure S18: ^{1}H-NMR (400 MHz, C_7D_8) used for quantification of N,N-dimethyl-4-vinylaniline (11).

1-Ethyl-3-vinylbenzene (12)

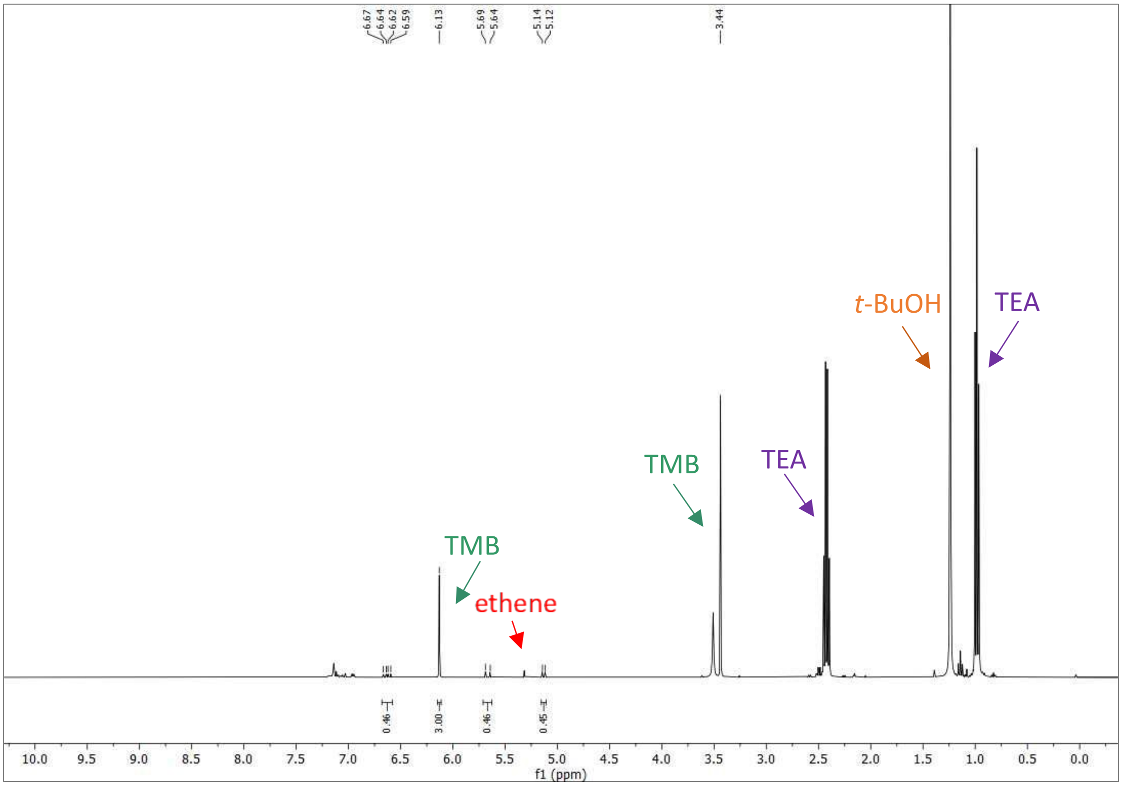

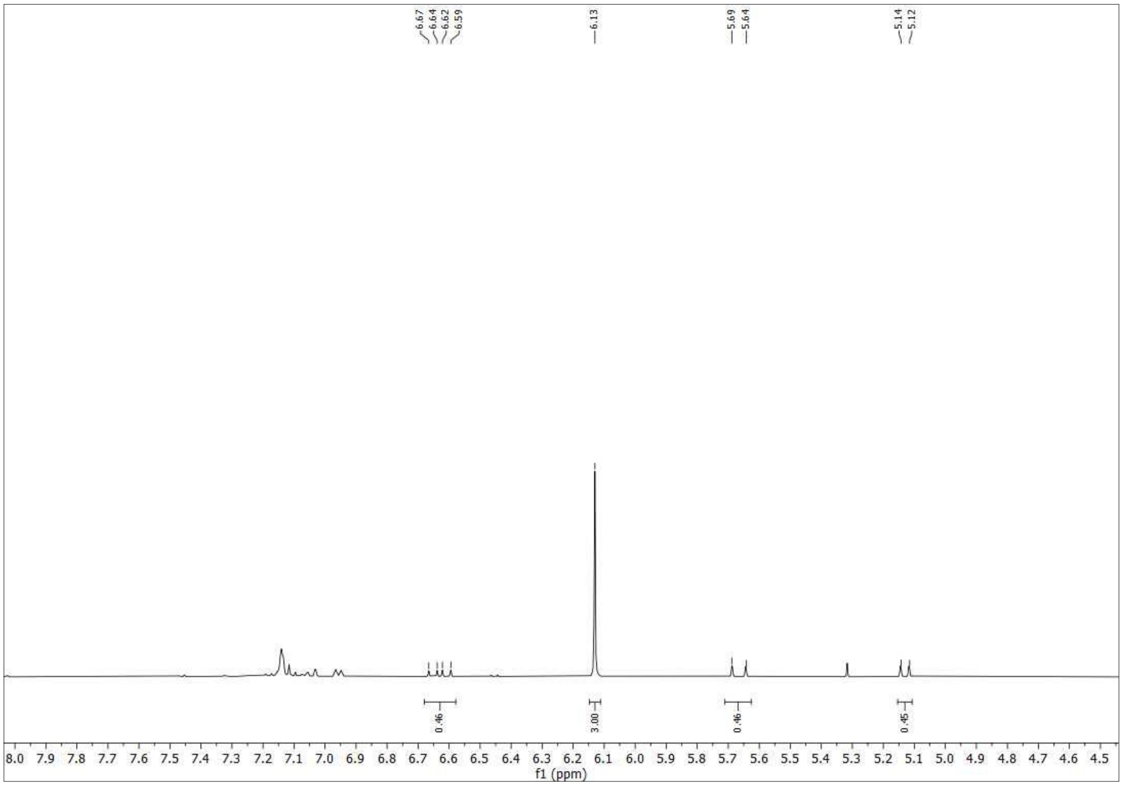

Figure S19: ^{1}H-NMR (400 MHz, C_7D_8) used for quantification of 1-ethyl-3-vinylbenzene (12).

1-Ethyl-4-vinylbenzene (13)

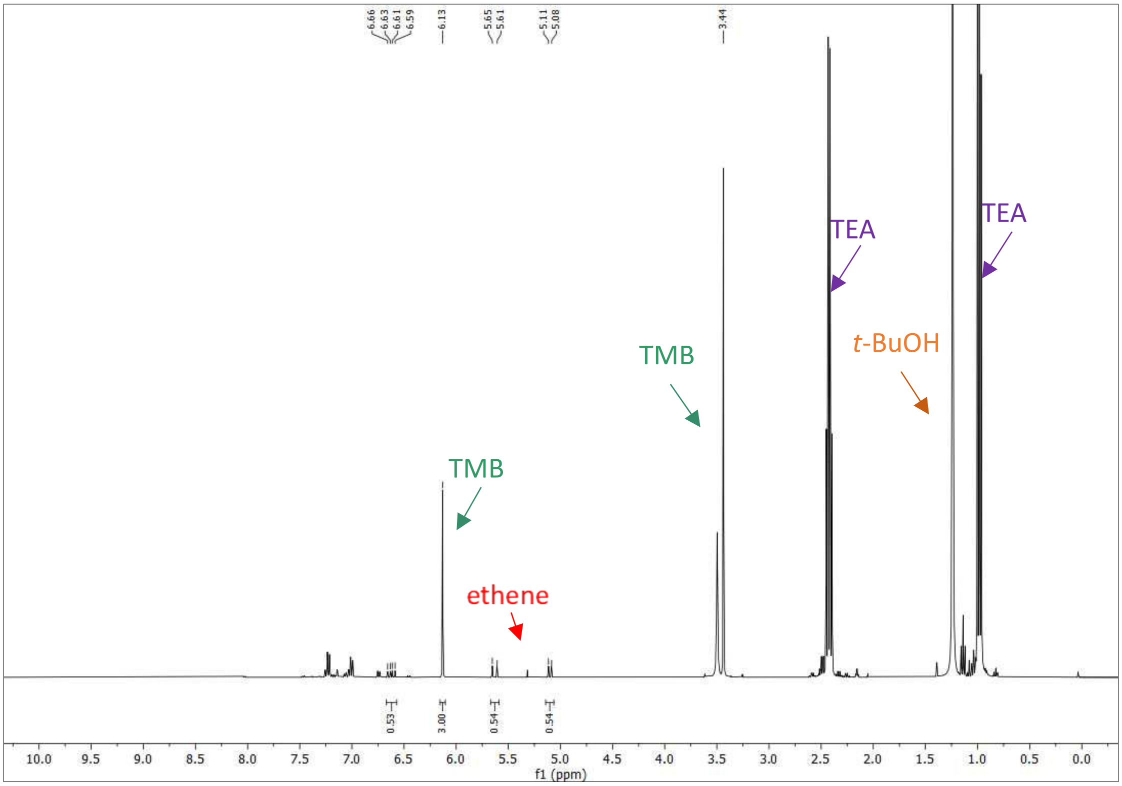

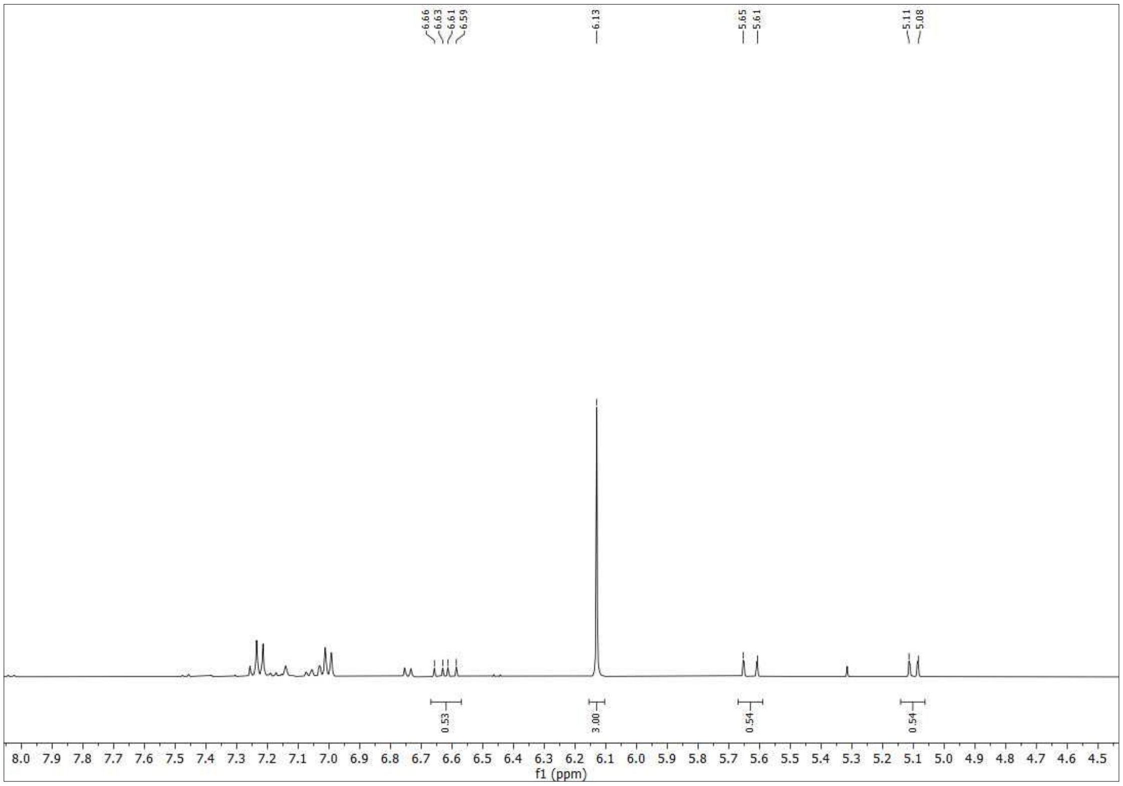

Figure S20: ^{1}H-NMR (400 MHz, C_7D_8) used for quantification of 1-ethyl-4-vinylbenzene (13).

1-Ethyl-2-vinylbenzene (14)

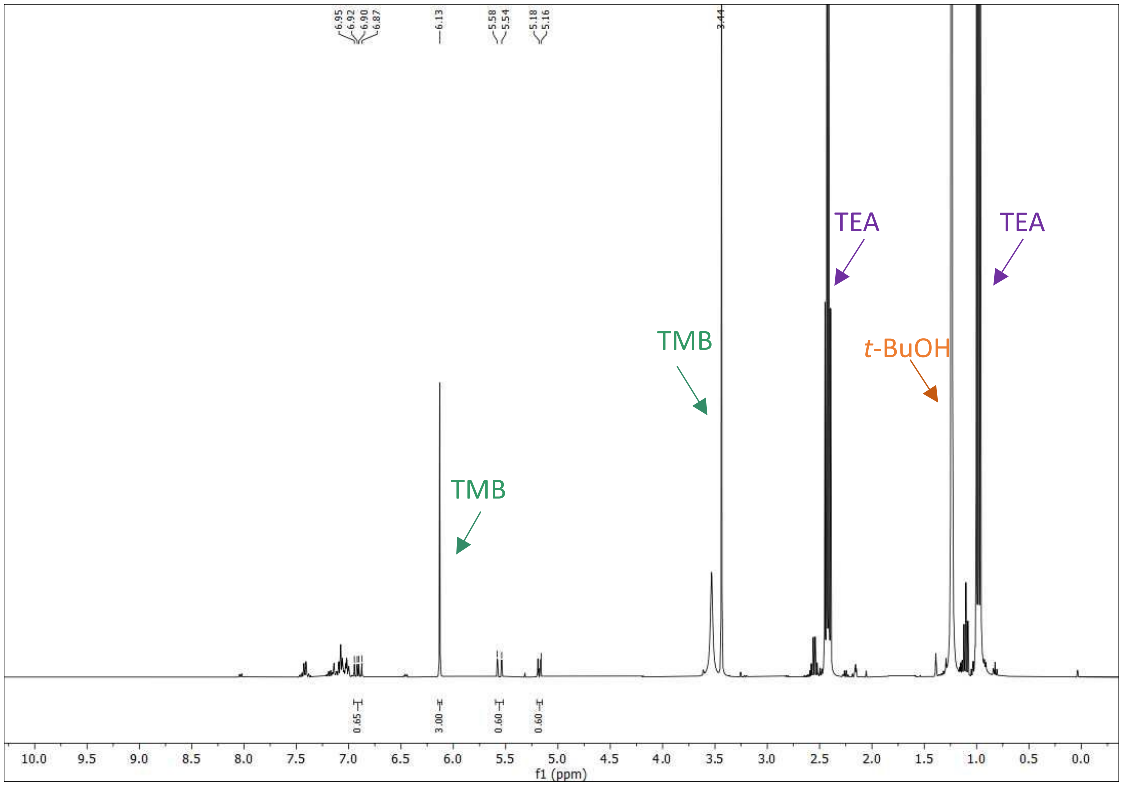

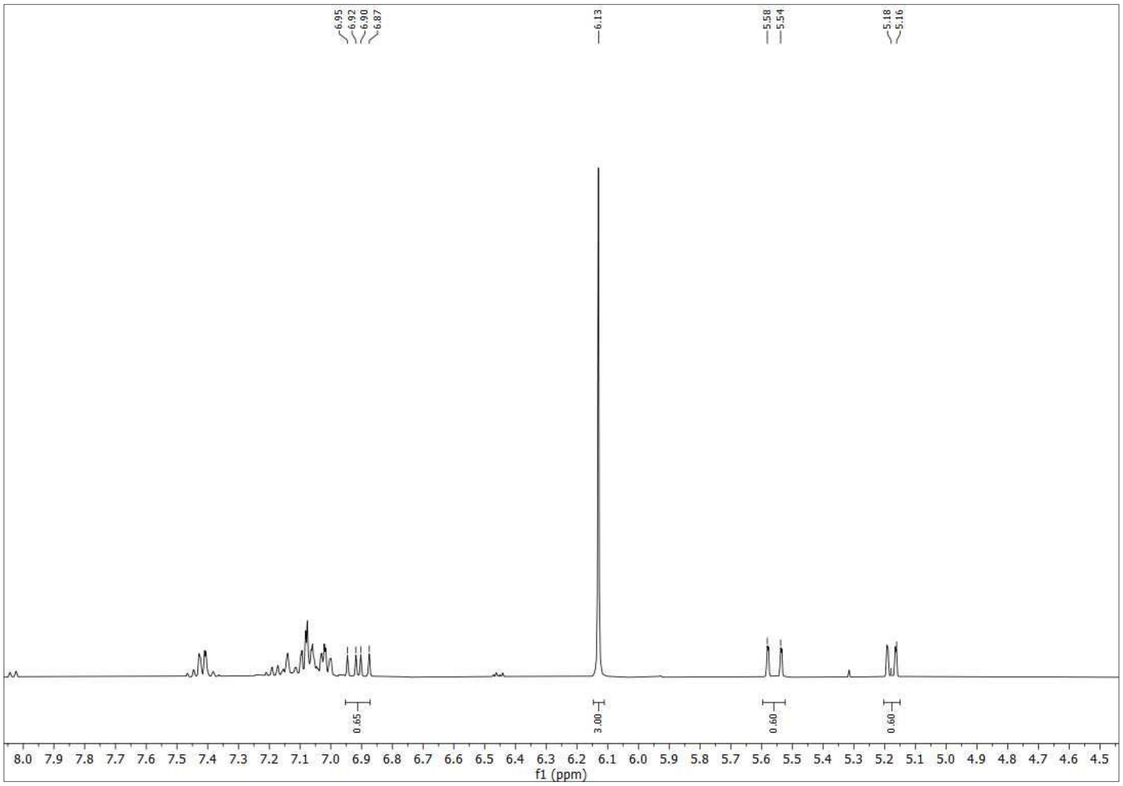

Figure S21: ^{1}H-NMR (400 MHz, C_7D_8) used for quantification of 1-ethyl-2-vinylbenzene (14).

tert-Butyl (4-vinylphenyl)carbamate (15)

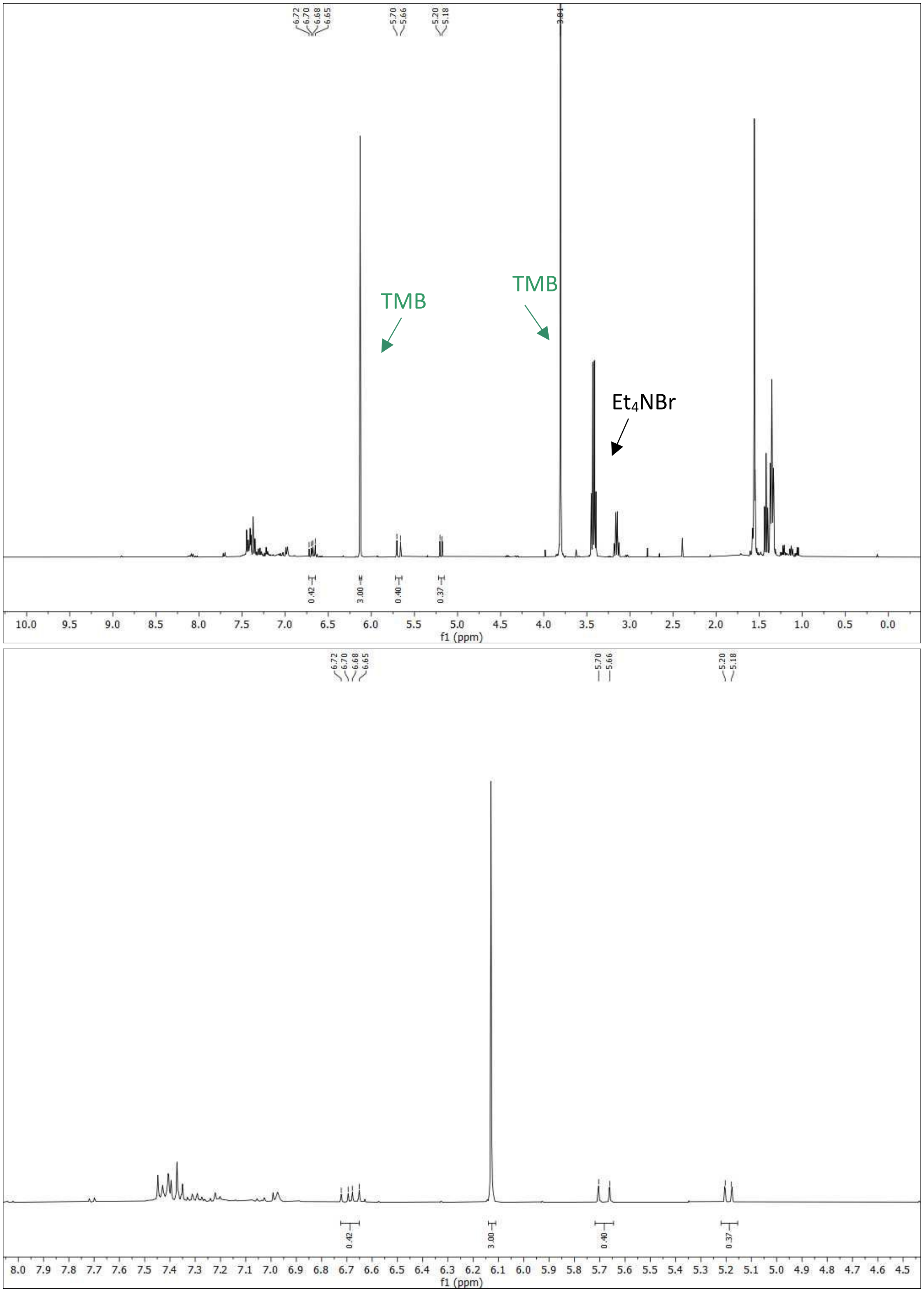

Figure S22: ^{1}H-NMR (400 MHz, C_7D_8) used for quantification of tert-butyl (4-vinylphenyl)carbamate (15).

4-Vinylphenol (16)

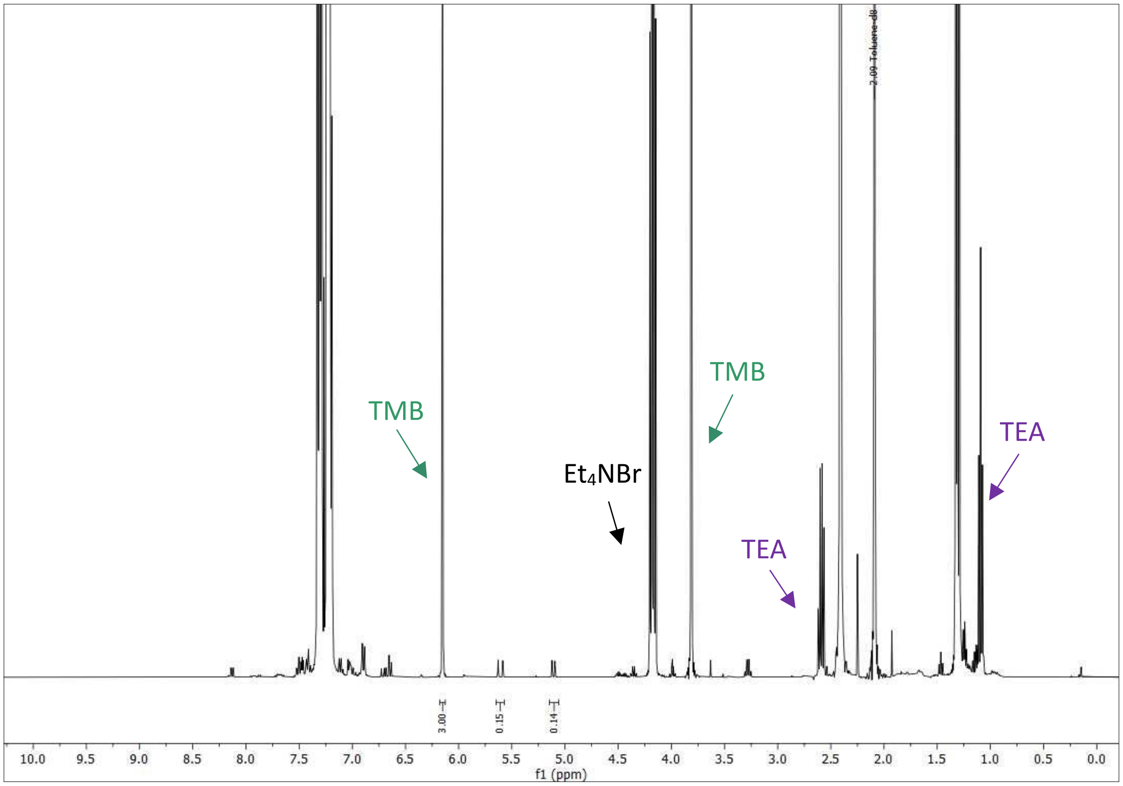

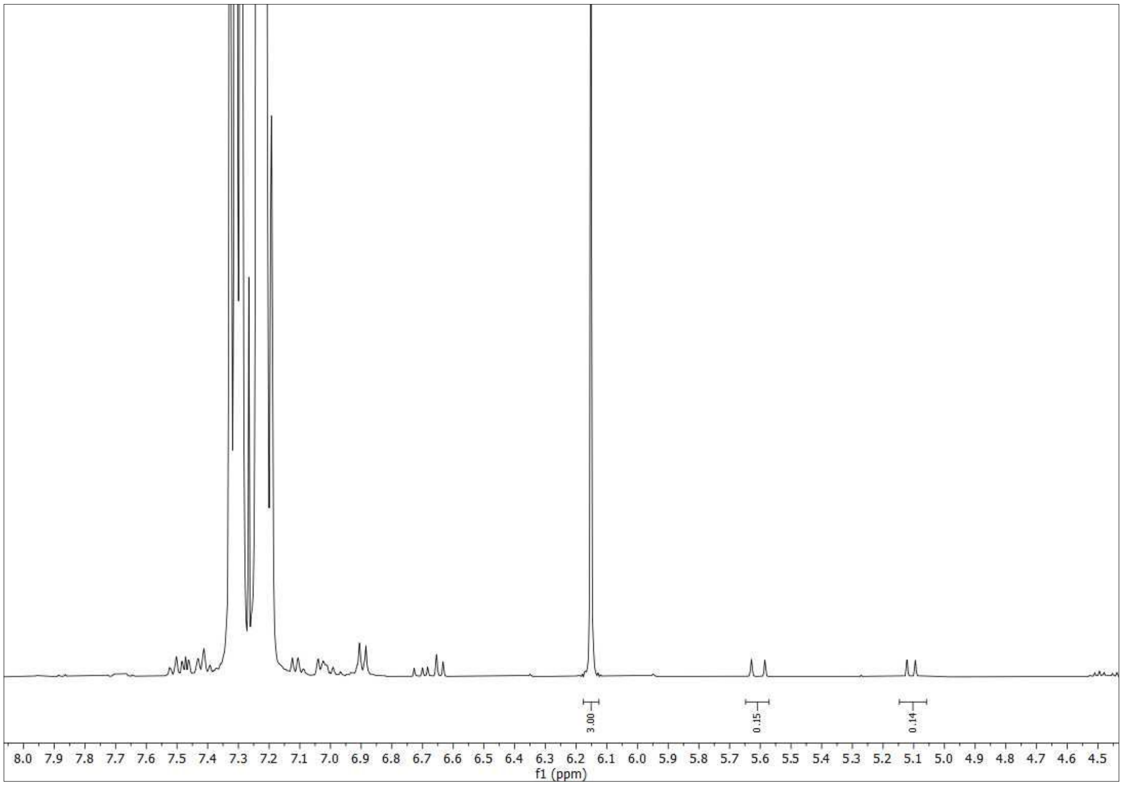

Figure S23: ^{1}H-NMR (400 MHz, C_7D_8) used for quantification of 4-vinylphenol (16).

3-Vinylpyridine (20)

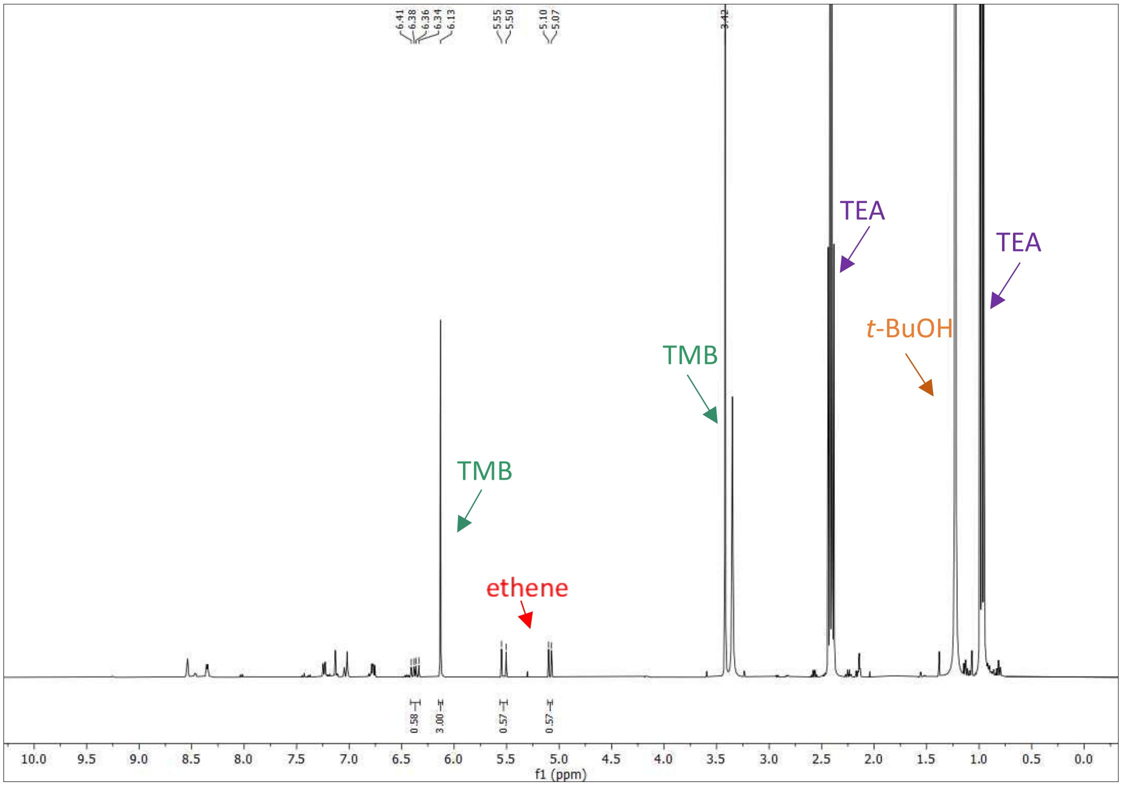

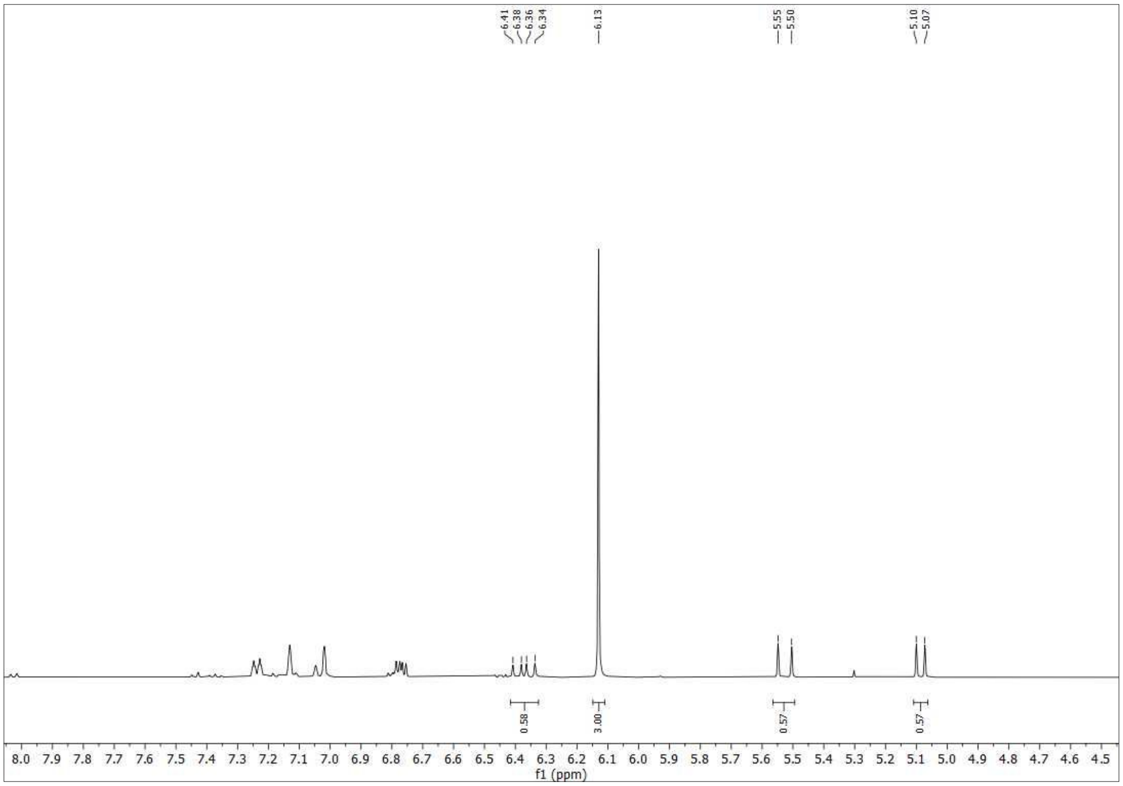

Figure S24: ^{1}H-NMR (400 MHz, C_7D_8) used for quantification of 3-vinylpyridine (20).

References

(1) An, N.; Ainembabazi, D.; Reid, C.; Samudrala, K.; Wilson, K.; Lee, A. F.; Voutchkova-Kostal, A. Microwave-Assisted Decarbonylation of Biomass-Derived Aldehydes Using Pd-Doped Hydrotalcites. *ChemSusChem.* **2020**, *13* (2), 312-320. DOI: 10.1002/cssc.201901934.

(2) Richmond, E.; Moran, J. Ligand Control of E/Z Selectivity in Nickel-Catalyzed Transfer Hydrogenative Alkyne Semireduction. *J. Org. Chem.* **2015**, *80* (13), 6922-6929. DOI: 10.1021/acs.joc.5b01047.

(3) Jiang, Y.; Fang, W.-Y.; Rakesh, K. P.; Qin, H.-L. Copper-Catalyzed Mild Desulfonylation of Vinyl Sulfonyl Molecules. *Org. Chem. Front.* **2020**, *7* (13), 1696-1702. DOI: 10.1039/D0QO00468E.

(4) Aukland, M. H.; Talbot, F. J. T.; Fernández-Salas, J. A.; Ball, M.; Pulis, A. P.; Procter, D. J. An Interrupted Pummerer/Nickel-Catalysed Cross-Coupling Sequence. *Angew. Chem., Int. Ed.* **2018**, *57* (31), 9785-9789. DOI: 10.1002/anie.201805396.

(5) Liu, K.-J.; Jiang, S.; Lu, L.-H.; Tang, L.-L.; Tang, S.-S.; Tang, H.-S.; Tang, Z.; He, W.-M.; Xu, X. Bis(methoxypropyl) Ether-Promoted Oxidation of Aromatic Alcohols into Aromatic Carboxylic Acids and Aromatic Ketones with O_2 Under Metal- and Base-Free Conditions. *Green Chem*. **2018**, *20* (13), 3038-3043. DOI: 10.1039/C8GC00223A.

(6) Wang, C.; Gong, S.; Liang, Z.; Sun, Y.; Cheng, R.; Yang, B.; Liu, Y.; Yang, J.; Sun, F. Ligand-Promoted Iridium-Catalyzed Transfer Hydrogenation of Terminal Alkynes with Ethanol and Its Application. *ACS Omega*. **2019**, *4* (14), 16045-16051. DOI: 10.1021/acsomega.9b02191.

(7) Gordillo, Á.; de Jesús, E.; López-Mardomingo, C. Consecutive Palladium-Catalyzed Hiyama–Heck Reactions in Aqueous Media Under Ligand-Free Conditions. *ChemComm*. **2007**, (39), 4056-4058. DOI: 10.1039/B707583A

(8) Zhang, X.; Jiang, Y.; Fei, H. UiO-Type Metal–Organic Frameworks with NHC or Metal–NHC Functionalities for N-Methylation Using CO_2 as The Carbon Source. *ChemComm*. **2019**, *55* (79), 11928-11931. DOI: 10.1039/C9CC06659D.

(9) Denmark, S. E.; Butler, C. R. Vinylation of Aryl Bromides Using an Inexpensive Vinylpolysiloxane. *Org. Lett*. **2006**, *8* (1), 63-66. DOI: 10.1021/ol052517r.

(10) Falk, A.; Cavalieri, A.; Nichol, G. S.; Vogt, D.; Schmalz, H.-G. Enantioselective Nickel-Catalyzed Hydrocyanation Using Chiral Phosphine-Phosphite Ligands: Recent Improvements and Insights. *Adv. Synth. Catal*. **2015**, *357* (14-15), 3317-3320. DOI: 10.1002/adsc.201500644.

(11) Lafaye, K.; Nicolas, L.; Guérinot, A.; Reymond, S.; Cossy, J. Lewis Basicity Modulation of N-Heterocycles: A Key for Successful Cross-Metathesis. *Org. Lett*. **2014**, *16* (19), 4972-4975. DOI: 10.1021/ol502016h.

(12) Akram, M. O.; Shinde, P. S.; Chintawar, C. C.; Patil, N. T. Gold(I)-Catalyzed Cross-Coupling Reactions of Aryldiazonium Salts with Organostannanes. *Org. Biomol*. *Chem.* **2018**, *16* (16), 2865-2869. DOI: 10.1039/C8OB00630J.
